# The genomic landscape of metastatic histologic special types of invasive breast cancer

**DOI:** 10.1038/s41523-020-00195-4

**Published:** 2020-10-14

**Authors:** Fresia Pareja, Lorenzo Ferrando, Simon S. K. Lee, Francisco Beca, Pier Selenica, David N. Brown, Amir Farmanbar, Arnaud Da Cruz Paula, Mahsa Vahdatinia, Hong Zhang, Gabriele Zoppoli, Hannah Y. Wen, Edi Brogi, Mark E. Robson, Pedram Razavi, Sarat Chandarlapaty, Britta Weigelt, Jorge S. Reis-Filho

**Affiliations:** 1 Department of Pathology, Memorial Sloan Kettering Cancer Center, New York, NY USA; 2 Department of Internal Medicine, University of Genova, Genova, Italy; 3 Department of Pathology, Stanford University School of Medicine, Stanford, CA USA; 4 Department of Surgery, Memorial Sloan Kettering Cancer Center, New York, NY USA; 5 IRCCS Ospedale Policlinico San Martino Genova, Genova, Italy; 6 Department of Medicine, Memorial Sloan Kettering Cancer Center, New York, NY USA

**Keywords:** Breast cancer, Breast cancer

## Abstract

Histologic special types of breast cancer (BC) account for ~20% of BCs. Large sequencing studies of metastatic BC have focused on invasive ductal carcinomas of no special type (IDC-NSTs). We sought to define the repertoire of somatic genetic alterations of metastatic histologic special types of BC. We reanalyzed targeted capture sequencing data of 309 special types of BC, including metastatic and primary invasive lobular carcinomas (ILCs; *n* = 132 and *n* = 127, respectively), mixed mucinous (*n* = 5 metastatic and *n* = 14 primary), micropapillary (*n* = 12 metastatic and *n* = 8 primary), and metaplastic BCs (*n* = 6 metastatic and *n* = 5 primary), and compared metastatic histologic special types of BC to metastatic IDC-NSTs matched according to clinicopathologic characteristics and to primary special type BCs. The genomic profiles of metastatic and primary special types of BC were similar. Important differences, however, were noted: metastatic ILCs harbored a higher frequency of genetic alterations in *TP53*, *ESR1*, *FAT1*, *RFWD2*, and *NF1* than primary ILCs, and in *CDH1*, *PIK3CA*, *ERBB2*, *TBX3*, *NCOR1*, and *RFWD2* than metastatic IDC-NSTs. Metastatic ILCs displayed a higher mutational burden, and more frequently dominant APOBEC mutational signatures than primary ILCs and matched metastatic IDC-NSTs. *ESR1* and *NCOR* mutations were frequently detected in metastatic mixed mucinous BCs, whereas *PIK3CA* and *TP53* were the most frequently altered genes in metastatic micropapillary and metaplastic BCs, respectively. Taken together, primary and metastatic BCs histologic special types have remarkably similar repertoires of somatic genetic alterations. Metastatic ILCs more frequently harbor APOBEC mutational signatures than primary ILCs and metastatic IDC-NSTs.

## Introduction

Breast cancer (BC) is heterogeneous and comprises various entities with divergent phenotype, biology, and clinical presentation^1,2^. There are over 20 histologic special types of BC recognized by The World Health Organization (WHO), accounting for ~20% of all BCs^3^. Large sequencing studies have focused on invasive ductal carcinoma of no special type (IDC-NSTs), the most common histologic form of BC^4–9^, and data on the genomic landscape of histologic special types, particularly in the metastatic setting, are scarce. These studies have shown that although the repertoire of somatic genetic alterations found in metastatic BCs is remarkably similar to that of primary tumors, *TP53*, *ESR1*, *ARID1A*, *ERBB2*, *GATA3*, *KMT2C*, *NCOR1*, *NF1*, and *RB1* have been found to be significantly more frequently mutated in metastatic disease^7,10,11^. In addition, estrogen receptor (ER)-positive metastatic BCs have been shown to more frequently display the APOBEC mutagenesis and homologous recombination DNA repair deficiency (HRD) processes than primary ER-positive disease^7,10^.

Massively parallel sequencing studies by our group and others have revealed that some histologic special types of BC are underpinned by highly recurrent or even pathognomonic genetic alterations, including *ETV6-NTRK3* fusion gene in secretory carcinoma, and *MYB/MYBL1* rearrangements or *MYB* amplification in adenoid cystic carcinoma^2,12–14^. Furthermore, other primary special types of BC, albeit not driven by pathognomonic fusion genes or somatic mutations, have been found to harbor repertoires of genetic alterations that differ from those of primary IDC-NST^15–23^. In addition to *CDH1* mutations, primary invasive lobular carcinomas (ILCs) have been shown to display an enrichment in mutations affecting *PIK3CA*, *PTEN*, *TBX3*, *FOXA1, AKT1*, *ARID1A*, *ERBB2*, and *ERBB3* (refs ^21,23^), primary mucinous BCs harbor a lower frequency 1q gains, 16q losses, and *PIK3CA* and *TP53* mutations than ER-positive/HER2-negative IDC-NSTs matched by clinical characteristics^15,24^, micropapillary BCs display a repertoire of genetic alterations comparable to that of common forms of BCs, with frequent mutations in *PIK3CA*, *TP53*, *GATA3*, and *MAP2K4* (ref. ^25^), and metaplastic BCs, compared to triple-negative IDC-NSTs, more frequently harbor mutations affecting genes of the PI3K/AKT/mTOR and canonical Wnt pathways^22,26^.

Here, through the reanalysis of targeted sequencing data generated with an FDA-approved multigene sequencing assay^11^, we sought to define the repertoire of somatic genetic alterations of metastatic ILCs, mixed mucinous, micropapillary, and metaplastic BCs, and determine whether the landscape of somatic mutations and copy number alterations (CNAs) of metastatic special types of BC is distinct from that of their primary counterparts or of metastatic IDC-NSTs.

## Results

### Clinicopathologic characteristics

We reanalyzed the sequencing data corresponding to 309 samples of histologic special types of BC reported by Razavi et al.^11^, comprising 154 and 155 primary and metastatic BCs, respectively (Supplementary Tables 1–2). A total of 127 primary and 132 metastatic ILCs, 14 primary and five metastatic mixed mucinous BCs, 8 primary and 12 metastatic micropapillary BCs, and 5 primary and 6 metastatic metaplastic BCs were included in this study. Most primary and metastatic ILCs (95 and 81%), mixed mucinous BCs (79 and 100%), and micropapillary BCs (63 and 75%) were ER-positive/HER2-negative, whereas 80 and 83% of primary and metastatic metaplastic BCs were of triple-negative phenotype, respectively (Supplementary Tables 1, 2). We observed an enrichment of HER2-positive (7%) and ER-negative/HER2-negative (12%) phenotypes in metastatic ILCs, as compared to primary ILCs (2%, each), whereas primary ILCs were more frequently of ER-positive/HER2-negative (95%) phenotype than metastatic ILCs (81%; *P* = 1.6 × 10^−3^; Supplementary Tables 1, 2).

### Repertoire of somatic genetic alterations in primary and metastatic ILCs

**Fig. 1:**
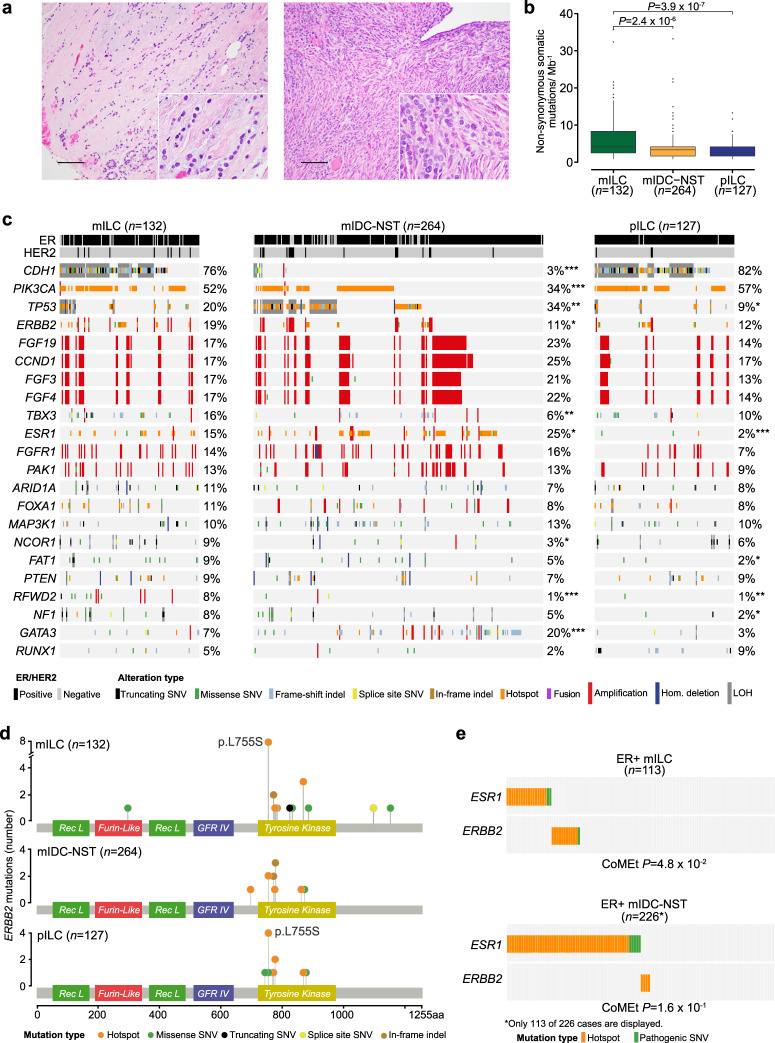
Repertoire of genetic alterations in primary and metastatic invasive lobular carcinomas of the breast. **a** Representative photomicrographs of a H&E-stained primary breast invasive lobular carcinoma (pILC; left) and a metastatic lobular carcinoma (mILC) involving ovarian stroma (right). Scale bars, 50 µm. **b** Boxplots depicting the non-synonymous tumor mutation burden of mILCs (*n* = 132), metastatic invasive ductal carcinomas of no special type matched by age, menopausal status, and estrogen receptor (ER)/HER2 status (mIDC-NSTs; *n* = 264), and pILCs (*n* = 127). Mann–Whitney *U* test, two-tailed. **c** Comparison of the cancer genes most frequently affected by non-synonymous somatic mutations, amplifications, or homozygous deletions in mILCs (*n* = 132), metastatic age-, menopausal status-, and ER/HER2 status-matched mIDC-NSTs (*n* = 264) and pILCs (*n* = 127). Cases are shown in columns and genes in rows. Mutation types are color-coded according to the legend. ER/HER2 status are shown on phenobars (top). *P < 0.05, **P < 0.01, ***P < 0.001; Fisher’s exact test, two-tailed. **d** Schematic representation of the protein domains of *ERBB2* and the somatic mutations in metastatic mILCs (*n* = 132), mIDC-NSTs matched by clinicopathologic characteristics (*n* = 264) and pILCs (*n* = 127). Mutations are color-coded according to the legend, and their frequency is represented by the height of each lollipop (*y*-axis). **e** Mutual exclusivity analysis of *ESR1* and *ERBB2* hotspot, and oncogenic/likely oncogenic mutations in ER-positive mILCs (*n* = 113) and mIDC-NSTs (*n* = 226). Hom. homozygous, Indel insertion/deletion, LOH loss of heterozygosity, SNV single nucleotide variant.

The ILCs included in this study were of classical type, characterized by a uniform population of small to medium-sized tumors cells with a dyshesive growth pattern, usually arranged in strands and single files (Fig. 1a). We compared the repertoire of somatic genetic alterations between primary and metastatic ILCs and observed that the non-synonymous tumor mutation burden (TMB) of metastatic ILCs (median = 4.2, 95% CI = 0.8–20.5) was significantly higher than that of primary ILCs (median 2.5, 95% CI = 0.8–8.2, *P* = 3.9 × 10^−7^, Mann–Whitney *U* test; Fig. 1b). The genes most frequently altered in metastatic ILCs (*n* = 132) overlapped with those reported in primary tumors, including *CDH1* (76%), *PIK3CA* (52%), *TP53* (20%), *ERBB2* (19%), *FGF19*, *CCND1*, *FGF3*, *FGF4* (each, 17%), and *TBX3* (16%). Other frequently altered genes in metastatic ILCs included *ARID1A* and *FOXA1* (11%, each), *MAP3K1* (10%), and *PTEN* (9%; Fig. 1c). As compared to primary ILCs (*n* = 127), metastatic ILCs (*n* = 132) more frequently harbored genetic alterations affecting *TP53* (20% vs 9%, respectively; *P* = 1.3 × 10^−2^), *ESR1* (15% vs 2%, respectively; *P* = 3 × 10^−4^), *FAT1* (9% vs 2%, respectively; *P* = 1.1 × 10^−2^), *RFWD2* (8% vs 1%, respectively; *P* = 5.4 × 10^−3^), and *NF1* (8% vs 2%, respectively; *P* = 1.9 × 10^−2^; Fig. 1c, and Supplementary Tables 3, 4). We also observed that *ERBB2* was numerically more frequently altered in metastatic ILCs than in primary ILCs (19% vs 12%, respectively; *P* = 1.2 × 10^−1^; Fig. 1c): 12% (16/132) of metastatic ILCs harbored *ERBB2* mutations, 5% (6/132) *ERBB2* gene amplification, and 2% (3/132) harbored both *ERBB2* mutations and gene amplification. In 10% (13/132) of cases the *ERBB2* mutations were hotspot mutations in the kinase domain (Fig. 1c).

Next, we compared the mutational repertoire of metastatic ILCs (*n* = 132) to that of metastatic IDC-NSTs matched by age, menopausal status, and ER/HER2 status at a 1:2 ratio (*n* = 264). The non-synonymous TMB of metastatic ILCs (median = 4.2, 95% CI = 0.8–20.5) was significantly higher than that of metastatic IDC-NSTs matched by clinicopathologic characteristics (median = 3.3, 95% CI = 0.8–12.8; *P* = 2.4 × 10^−6^; Mann–Whitney *U* test; Fig. 1b). Compared to age, menopausal, and ER/HER2 status-matched metastatic IDC-NSTs (*n* = 264), metastatic ILCs (*n* = 132) harbored a significantly higher frequency of genetic alterations affecting *CDH1* (76% vs 3%, respectively; *P* = 9.8 × 10^−54^), *PIK3CA* (52% vs 34%, respectively; *P* = 7 × 10^−4^), *ERBB2* (19% vs 11%, respectively; *P* = 2.8 × 10^−2^), *TBX3* (16% vs 6%, respectively; *P* = 3.6 × 10^−3^), *NCOR* (9% vs 3%, respectively; *P* = 1 × 10^−2^), *RFWD2* (8% vs 1%, respectively; *P* = 6 × 10^−4^), and a significantly lower frequency of genetic alterations affecting *TP53* (20% vs 34%, respectively; *P* = 3.3 × 10^−3^), *ESR1* (15% vs 25%, respectively; *P* = 2.8 × 10^−2^), and *GATA3* (7% vs 20%, respectively; *P* = 4 × 10^−4^) among others (Fig. 1c, and Supplementary Tables 3, 4).

Most *ERBB2* mutations identified in metastatic ILCs (*n* = 59%), primary ILCs (67%), and metastatic IDC-NSTs (45%) affected hotspot loci (Fig. 1d). Notably, the L755S *ERBB2* hotspot mutation was the most frequent in both metastatic (8/22; 36%) and primary ILCs (4/12; 33%; Fig. 1d), as previously reported^23^. This mutation, however, accounted for only 17% (2/12) of the *ERBB2* mutations detected in metastatic IDC-NSTs matched by clinicopathologic characteristics (Fig. 1d). Of note, we did not identify differences in pre-biopsy therapy of patients with metastatic ILCs harboring *ERBB2* L755S mutations, that could account for the observed enrichment (Supplementary Table 5).

Given the role of *ESR1* mutations and *ERBB2* mutations in endocrine therapy resistance in ER-positive metastatic BCs^11,27–31^, we sought to investigate their mutual exclusivity in ER-positive metastatic ILCs (*n* = 113) and IDC-NSTs (*n* = 226). We observed that hotspot mutations or pathogenic mutations affecting *ESR1* and *ERBB2* were mutually exclusive in metastatic ILCs (*P* = 4.8 × 10^−2^; CoMEt; Fig. 1e). These findings are consistent with those reported by Razavi et al.^11^, where *ESR1* and *ERBB2* mutations were found to be mutually exclusive in ER-positive/HER2-negative BCs regardless of their histologic subtype. Hence, akin to common cancer types of BC, *ESR1*, and *ERBB2* mutations are present in a mutually exclusive manner in metastatic ILCs, and may constitute mechanisms of resistance to endocrine therapy^11,27–31^. To define the repertoire of somatic genetic alterations present in ILCs, we combined the primary and metastatic ILCs of this study in one cohort (*n* = 259), and compared them to combined primary and metastatic IDC-NSTs, matched to the ILCs according to age, menopausal status, ER/HER2 status, and sample type at a 2:1 ratio (*n* = 518). This analysis revealed differences consistent with our findings when primary and metastatic ILCs were compared to IDC-NST separately. Combined primary and metastatic ILCs (*n* = 259) displayed a higher non-synonymous TMB (*P* = 1.8 × 10^−7^) than combined primary and metastatic IDC-NSTs (*n* = 518; Supplementary Fig. 1a). In addition, as compared to combined primary and metastatic IDC-NSTs, combined primary and metastatic ILCs harbored a higher frequency in genetic alterations affecting *CDH1* (79% vs 3%; *P* = 1.0 × 10^−116^), *PIK3CA* (54% vs 37%; *P* = 5.3 × 10^−6^), *ERBB2* (15% vs 8%, *P* = 1.6 × 10^−3^), *TBX3* (13% vs 6%; *P* = 1.4 × 10^−3^), *ARID1A* (10% vs 5%; *P* = 3.4 × 10^−2^), *NCOR1* (8% vs 4%, *P* = 4 × 10^−2^), *RUNX1* (7% vs 3%: *P* = 2.7 × 10^−2^), and *RFWD2* (5% vs 1%, *P* = 7 × 10^−4^), and a lower frequency of genetic alterations affecting *TP53* (14% vs 33%; *P* = 2.6 × 10^−8^), *ESR1* (9% vs 15%, *P* = 2.2 × 10^−2^), and *GATA3* (5% vs 19%; *P* = 1.2 × 10^−8^; Supplementary Fig. 2)

### Repertoire of somatic genetic alterations in primary and metastatic mixed mucinous BCs

**Fig. 2:**
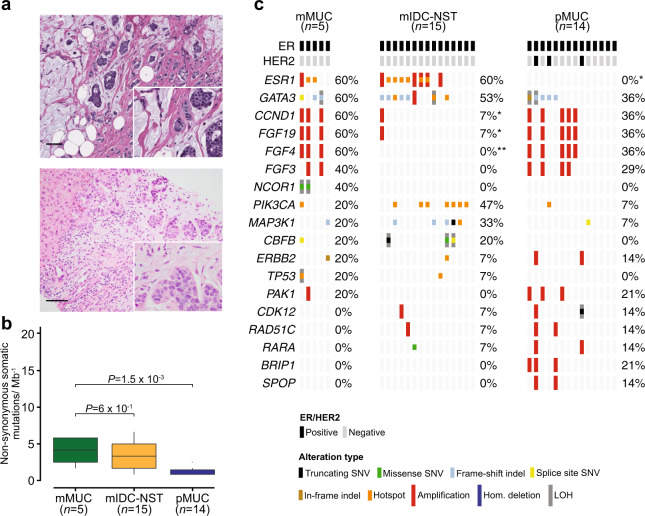
Repertoire of genetic alterations in primary and metastatic mixed mucinous breast cancers. **a** Representative photomicrographs of a H&E-stained primary mixed mucinous breast cancer (pMUC; top), and a metastatic mixed mucinous breast cancer (mMUC) involving liver (bottom). Scale bars in **a**, 100 μm (top) and 50 μm (bottom). **b** Boxplots depicting the non-synonymous tumor mutation burden in mMUCs (*n* = 5), metastatic invasive ductal carcinoma of no special type matched by age, menopausal status, and estrogen receptor (ER)/HER2 status (mIDC-NST; *n* = 15), and pMUCs (*n* = 14). Mann–Whitney *U* test, two-tailed. **c** Comparison of the cancer genes most frequently affected by non-synonymous somatic mutations, amplifications, or homozygous deletions in mMUCs (*n* = 5), in age-, menopausal status-, and ER/HER2 receptor status-matched mIDC-NSTs (*n* = 15), and in pMUCs (*n* = 14). Cases are shown in columns and genes in rows. Mutation types are color-coded according to the legend. ER/HER2 status are shown on phenobars (top). *P < 0.05; Fisher’s exact test, two-tailed. Indel insertion/deletion, LOH loss of heterozygosity, SNV single nucleotide variant.

The mixed mucinous BCs analyzed in this study were characterized by areas of tumor cells floating in lakes of mucin admixed with areas of IDC-NST (Fig. 2a, b). Metastatic mixed mucinous BCs harbored a significantly higher non-synonymous TMB (median = 4.2, 95% CI = 1.7–5.8) than primary mixed mucinous BCs (median = 0.8, 95% CI = 0.8–2.2, *P* = 1.5 × 10^−3^, Mann–Whitney *U* test), but comparable to that of metastatic IDC-NSTs matched according to clinical features (median = 3.3, 95% CI = 0.8–6.4, *P* = 6 × 10^−1^; Fig. 2b). The repertoire of genetic alterations of metastatic mixed mucinous BCs (*n* = 5) in this study was similar to that of primary mucinous/mixed BCs^15,16,32^. Although based on a small number of cases, this analysis revealed that the genes recurrently altered in metastatic mixed mucinous BC and not altered in primary mixed mucinous BCs of this study, and pure/ mixed mucinous BCs reported by our group and others^15,16,32^ included *ESR1* (60% vs 0%, respectively; *P* = 1 × 10^−2^) and *NCOR* (40% vs 0%, respectively; *P* = 6 × 10^−2^; Fig. 2c). In agreement with previous studies^33^, compared to metastatic IDC-NSTs matched by clinical features, metastatic mixed mucinous BCs harbored a higher frequency of 11q13.3 amplification (60% vs 7%, respectively; *P* = 3.2 × 10^−2^; Fig. 2c).

### Repertoire of somatic genetic alterations in primary and metastatic micropapillary BCs

**Fig. 3:**
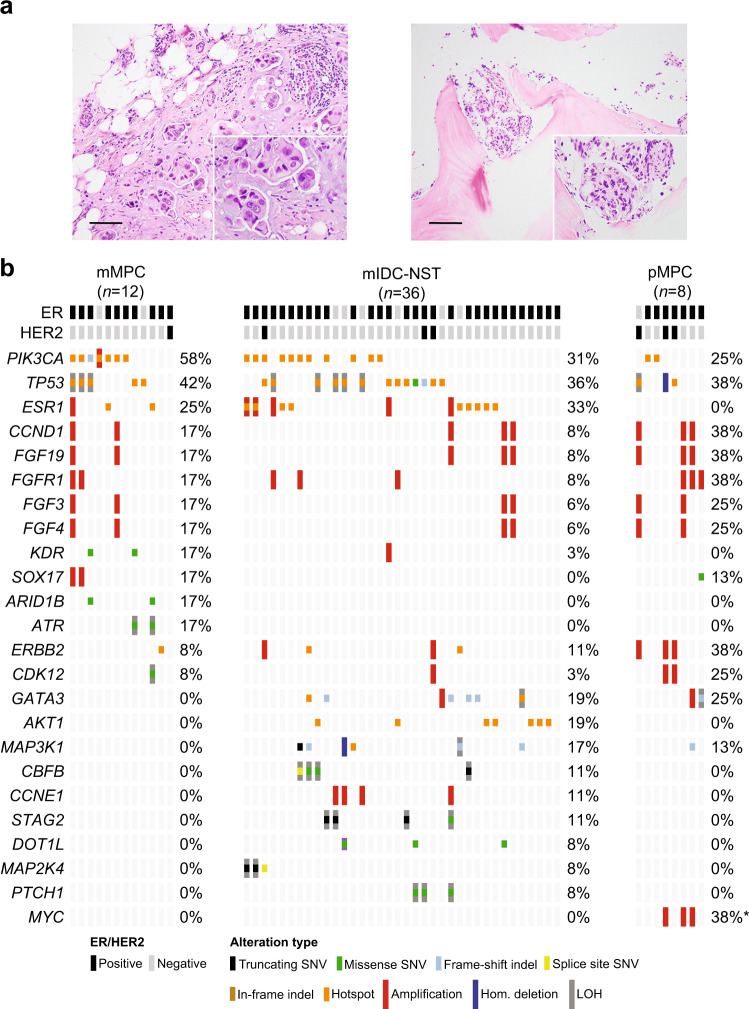
Repertoire of genetic alterations in primary and metastatic micropapillary breast cancers. **a** Representative photomicrographs of a H&E-stained primary micropapillary breast cancer (pMPC; left) and of a metastatic micropapillary BC (mMPC) involving bone (right). Scale bars, 50 µm. **b** Comparison of the cancer genes most frequently affected by non-synonymous somatic mutations, amplifications or homozygous deletions in mMPCs (*n* = 12), in age-, menopausal status-, and estrogen receptor (ER)/HER2 receptor status-matched metastatic invasive ductal carcinoma of no special type (mIDC-NST; *n* = 36), and pMPC (*n* = 8). Cases are shown in columns and genes in rows. Genetic alterations are color-coded according to the legend. ER/HER2 status are shown on phenobars (top). *P < 0.05, Fisher’s exact test; two-tailed. Indel insertion/deletion, LOH loss of heterozygosity, SNV single nucleotide variant.

The micropapillary BCs included in this study were characterized by morula-like clusters of tumor cells without a fibrovascular core within pseudo-vascular spaces (Fig. 3a). We observed no significant differences in the non-synonymous TMB of metastatic micropapillary BCs (median = 1.2, 95% CI = 0.8–4) compared to that of primary micropapillary BCs (median = 1.2, 95% CI = 0.8–4; *P* = 1.3 × 10^−1^) or to that IDC-NSTs matched by clinical features (median = 3.3, 95% CI = 0.8–11.7; *P* = 3.9 × 10^−1^, Supplementary Fig. 1b). In a way akin to IDC-NSTs, the most frequently altered genes in metastatic and primary micropapillary BCs were *PIK3CA* (58 and 25%) and *TP53* (42 and 38%). Recurrent alterations in *ESR1* (25%), *KDR*, *ARID1B*, and *ATR* (17%, each) were restricted to metastatic micropapillary BCs (Fig. 3b). On the other hand, *MYC* gene amplification was more frequent in primary than in metastatic micropapillary BCs (38% vs 0%, respectively; *P* = 4.9 × 10^−2^; Fig. 3b).

### Repertoire of somatic genetic alterations in primary and metastatic metaplastic BCs

**Fig. 4:**
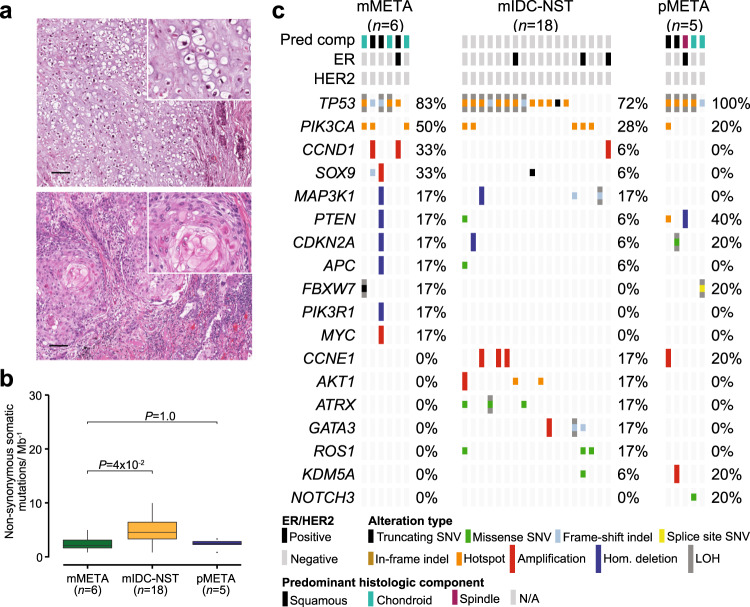
Repertoire of genetic alterations in primary and metastatic metaplastic breast cancers. **a** Representative photomicrographs of a H&E-stained primary metaplastic breast cancer (pMETA) with predominant chondroid differentiation (top), and of a metastatic metaplastic breast cancer (mMETA) with predominant squamous differentiation involving lung (bottom). Scale bars, 100 μm. **b** Boxplots depicting the non-synonymous mutation burden of metastatic metaplastic BCs (mMETA; *n* = 6), metastatic invasive ductal carcinomas of no special type (mIDC-NSTs; *n* = 18) matched by age, menopausal status, and estrogen receptor (ER)/HER2 status, and primary metaplastic BCs (pMETA; *n* = 5). Mann–Whitney *U* test, two-tailed. **c** Comparison of the cancer genes most frequently affected by non-synonymous somatic mutations, amplifications, or homozygous deletions in mMETA (*n* = 6), age, menopausal status, and ER/HER2 receptor status-matched metastatic invasive ductal carcinomas of no special type (mIDC-NSTs; *n* = 18), and primary metaplastic BCs (pMETA; *n* = 5). Cases are shown in columns and genes in rows. Genetic alterations are color-coded according to the legend. ER/HER2 status and predominant histologic component are shown on phenobars (top). No significant different mutation frequencies were found using Fisher’s exact test. Indel insertion/deletion, LOH loss of heterozygosity, SNV single nucleotide variant.

The metaplastic BCs analyzed in this study were histologically heterogeneous. Out of the six metastatic metaplastic BCs, three displayed predominantly chondroid and three predominantly squamous differentiation, whereas the five primary metaplastic BCs exhibited predominantly squamous (*n* = 2), chondroid (*n* = 2), or spindle (*n* = 1) differentiation (Fig. 4a). The non-synonymous TMB of metastatic metaplastic BCs (median = 2.1, 95% CI = 0.9–4.8) was significantly lower than that of metastatic IDC-NSTs matched by age, menopausal status, and ER/HER2 status (median = 4.6, 95% CI = 0.8–17.7; *P* = 4 × 10^−2^, Mann–Whitney *U* test), but comparable to that of primary metaplastic BCs (median = 2.5, 95% CI = 1–3.2; *P* = 1.0; Mann–Whitney *U* test; Fig. 4b). *TP53* was the most frequently altered gene in metastatic (83%) and primary (100%) metaplastic BCs. Genes altered in more than one sample in metastatic metaplastic BCs, and not altered in primary metaplastic BCs, included *CCND1* and *SOX9* (33% in metastatic vs 0% in primary, each, Fig. 4c). Akin to primary metaplastic BCs^22^, metastatic metaplastic BCs harbored mutations affecting Notch pathway genes, such as *FBXW7*, and PI3K/AKT pathway genes, such as *PTEN* and *PIK3R1* (Fig. 4c). No statistically significant differences were observed in metastatic metaplastic BCs compared to primary metaplastic BCs or to metastatic IDC-NSTs matched by clinical features (Fig. 4c), potentially due to the small sample size of metaplastic BCs included in the study. We have previously reported that *PIK3CA* mutations are enriched in primary metaplastic BCs with predominant squamous or spindle cell differentiation, and absent or remarkably rare in primary metaplastic BCs with chondroid differentiation^22^. In line with these findings, we identified one primary metaplastic BC with squamous differentiation harboring a *PIK3CA* C420R hotspot mutation, whereas the primary chondroid metaplastic BCs studied here were *PIK3CA* wild type. In contrast, we observed *PIK3CA* mutations in metastatic metaplastic BCs with squamous (1/3) or chondroid (2/3) differentiation (Fig. 4b). Taken together, these findings suggest that *PIK3CA* mutations, albeit rare in primary metaplastic BCs with chondroid differentiation, may occur a subset of these tumors in the metastatic setting. Due to the limited number of metaplastic BCs studied here, these findings should be considered hypothesis generating.

### Comparative analysis of gene CNAs between special types of BC and IDC-NSTs

We observed no significant differences in the frequency of gains/losses and amplifications/homozygous deletions in metastatic ILCs, mixed mucinous, micropapillary, and metaplastic BCs, when compared to primary tumors of their respective histologic type, or to metastatic IDC-NSTs matched by clinicopathologic characteristics (Supplementary Fig. 3a,b,e–j). Nonetheless, the fraction of the genome altered (FGA) of metastatic ILCs was found to be significantly higher than that of primary ILCs (*P* = 9.7 × 10^−3^, Mann–Whitney *U* test; Supplementary Fig. 4a). Compared to metastatic IDC-NSTs matched by clinicopathologic characteristics, however, metastatic ILCs displayed a significantly lower FGA (*P* = 2.5 × 10^−5^, Mann–Whitney *U* test; Supplementary Fig. 4a). Despite a lower FGA in the cohort of combined primary and metastatic ILCs compared to the combined primary and metastatic IDC-NSTs (*P* = 4.8 × 10^−9^), no differences in the frequency of amplifications and homozygous deletions between the two groups were identified (Supplementary Figs 3d and 4b). Nonetheless, we observed that the combined cohort of primary and metastatic ILCs harbored a higher frequency of 1q gains and 16q losses than combined primary and metastatic IDC-NSTs matched by clinicopathologic characteristics and sample type (Supplementary Fig. 3c). No statistically significant differences were observed in the FGA of metastatic mixed mucinous, micropapillary, or metaplastic BCs compared to their primary counterparts, or to age-, menopausal status-, and ER/HER2 status-matched metastatic IDC-NSTs (Supplementary Fig. 4c–e), potentially due to the small sample size of special types of BC other than ILCs analyzed in the study.

### Mutational signatures in special types of BC

**Fig. 5:**
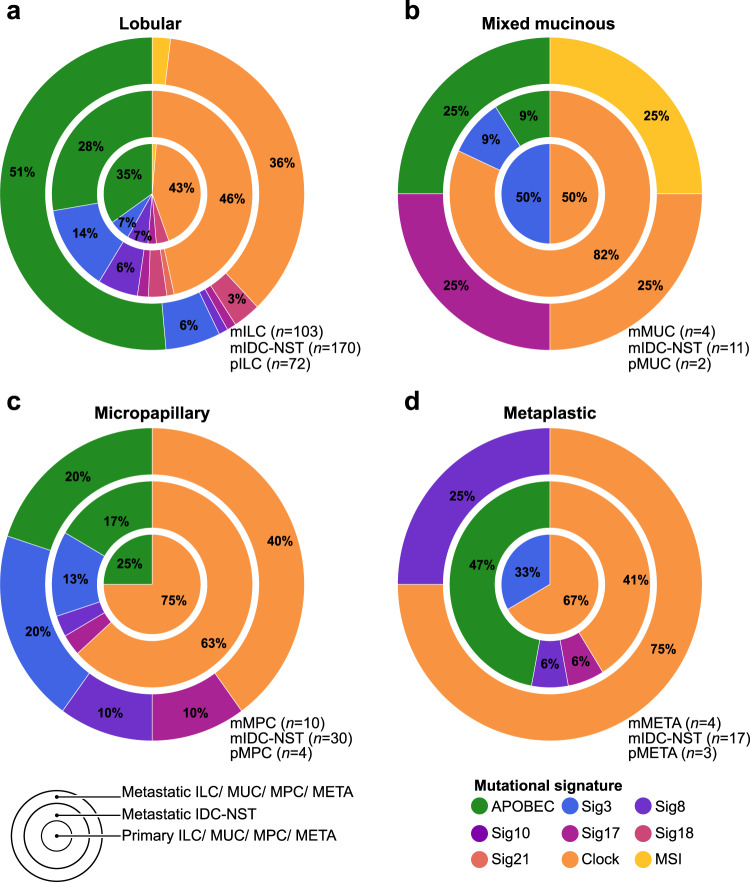
Mutational signatures in primary and metastatic histologic special types of breast cancer. Proportion of dominant mutational signatures in **a** metastatic invasive lobular carcinomas (mILC; *n* = 103), metastatic invasive ductal carcinomas of no special type matched (mIDC-NSTs) by age, menopausal status, and estrogen receptor (ER)/HER2 status (*n* = 170) and in primary ILCs (pILCs; *n* = 72); **b** in metastatic mixed mucinous breast cancer (BC; mMUC; *n* = 4), metastatic IDC-NSTs matched by clinicopathologic characteristics (*n* = 11), and primary mixed mucinous BCs (pMUC; *n* = 2); **c** in metastatic micropapillary BCs (mMPCs; *n* = 10), metastatic IDC-NSTs matched by clinicopathologic characteristics (*n* = 30), and primary micropapillary BCs (pMPC; *n* = 4), and **d** in metaplastic BCs (mMETA; *n* = 4), metastatic IDC-NSTs matched by clinicopathologic characteristics (*n* = 17), and primary metaplastic BCs (pMETA; *n* = 3) with sufficient number of mutations for appropriate mutational signature inference.

There is evidence to suggest that the mutational processes underpinning metastatic BCs may differ from those of primary BCs^7,10^. Hence, we sought to determine whether the mutational signatures of metastatic forms of special histologic subtypes of BC would differ from those of primary tumors and from common forms of BC. We inferred the dominant mutational signatures using SigMA^34^, using all synonymous and non-synonymous somatic mutations in those cases with at least five single nucleotide variants (SNVs) for an accurate signature inference (*n* = 202), as previously described^35^. We observed a significant enrichment for APOBEC mutational signatures 2 and 13 in metastatic ILCs (51%, 53/103) compared to primary ILCs (35%, 25/72, *P* = 3.2 × 10^−2^; Fisher’s exact test), and to metastatic IDC-NSTs matched by clinicopathologic features (28%, 47/170; *P* = 9.7 × 10^−5^, Fisher’s exact test; Fig. 5a and Supplementary Fig. 5a).

In addition, combined primary and metastatic ILCs compared to combined primary and metastatic IDC-NSTs displayed a significant enrichment in APOBEC mutational signatures (45% vs 22%; *P* = 2.1 × 10^−7^; Supplementary Fig. 5b,f). Consistent with the association of APOBEC processes with hypermutation, metastatic ILCs with a dominant APOBEC mutational signature (median = 8.3, 95% CI = 1.7–29.1) had a higher non-synonymous TMB than mILCs with a dominant non-APOBEC mutational signature (median = 4.2, 95% CI = 0.8–8.9; *P* = 5.5 × 10^−6^; Supplementary Fig. 6a). Of note, no differences in the percentage of tumor-infiltrating lymphocytes (TILs) were observed in metastatic ILCs with a dominant APOBEC mutational signature (*n* = 25; median = 10%, 95% CI = 1–35%) compared to metastatic ILC with a dominant mutational signature other than APOBEC (*n* = 21; median = 5%; 95% CI = 2–20%; *P* = 7.8 × 10^−1^), or to primary ILCs with a dominant APOBEC signature (*n* = 20; median = 10%; 95% CI = 1–55%; *P* = 8 × 10^−1^; Supplementary Fig. 6b). Similarly, no differences in the extent of TIL infiltration were observed between primary ILCs with a dominant APOBEC signature and those with a dominant mutational signature other than APOBEC (*n* = 33; median = 7%; 95% CI = 1–40%; *P* = 7 × 10^−1^; Supplementary Fig. 6b). In contrast, a dominant APOBEC mutational signature was present in 25% (1/4) and 20% (2/10) of metastatic mixed mucinous and micropapillary BCs, respectively, and in none of the metastatic metaplastic BCs (Figs 5b–d and Supplementary Fig. 5c–e). Of note, although one metastatic mixed mucinous BC displayed a dominant microsatellite instability mutational signature (Fig. 5b), it did not harbor genetic alterations in any of the core mismatch repair genes (Supplementary Table 4) and displayed a retained expression of MLH1, MSH2, MSH6, and PMS2 by immunohistochemical analysis (Supplementary Fig. 7a–e). Homologous recombination deficiency-related signature 3 was found to be dominant in 6% (6/103) of metastatic ILCs and in 20% (2/10) of metastatic micropapillary BCs (Fig. 5a–d). Nonetheless, none of these cases were found to harbor biallelic inactivation of HRD related genes^36^ included in the Memorial Sloan Kettering-Integrated Mutation Profiling of Actionable Targets (MSK-IMPACT) sequencing assay panel (Supplementary Table 4). Mutational signatures ascribed to aging were the most frequent dominant signatures in most of primary and metastatic micropapillary BCs (75 and 40%) and metaplastic BCs (67 and 75%), and were present in 1/4 metastatic and 1/2 primary mixed mucinous carcinomas (Fig. 5a–d).

## Discussion

Here, through the reanalysis of targeted sequencing data of primary and metastatic forms of histologic special types of BC, we have demonstrated that the repertoire of somatic genetic alterations in metastatic forms of histologic special subtypes of BC is generally similar to that of their primary counterparts. Notable differences were, however, were observed, such as an enrichment for genetic alterations affecting *ESR1*, mainly as hotspot mutations, in metastatic ILCs and metastatic mixed mucinous carcinomas. We also observed a higher frequency of *ERBB2* mutations in metastatic ILCs compared to primary ILCs, in agreement with previous studies of metastatic and relapsed ILCs^37,38^, and compared to age, menopausal status, and ER/HER2 status-matched metastatic IDC-NSTs. In addition, the spectrum of *ERBB2* mutations differed between metastatic ILCs and metastatic IDC-NSTs matched by clinical characteristics, given that 36% of *ERBB2* mutations targeted the L755 hotspot locus in metastatic ILCs, whereas this mutation accounted for only 17% in *ERBB2* mutations in metastatic IDC-NSTs. The basis for the apparent enrichment for L755 *ERBB2* mutations in metastatic ILCs warrants further investigation. The L755S mutation, however, has been shown to confer resistance to the tyrosine kinase inhibitor lapatinib, but not to the irreversible inhibitor neratinib^39,40^. In addition to the known *ERRB2* hotspot mutations, we detected the *ERRB2* X1097 splice mutation, whose biological impact and clinical significance remain to be determined. In agreement with previous studies^39,41^, all but two (89%) *ERBB2* mutated metastatic ILCs were HER2-negative by immunohistochemistry and/or fluorescence in situ hybridization (FISH), highlighting the need of molecularly stratified clinical trials in the metastatic setting.

The enrichment for genetic alterations affecting *TP53* and *RFWD2*, a ubiquitin ligase that targets p53 for degradation^42^, observed in metastatic ILCs compared to primary tumors might be reflective of the advanced stage of these patients. *FAT1*, a tumor suppressor that confers resistance to CDK4/6 inhibitors when inactivated^43^, was also found to be altered more frequently in metastatic ILCs. In addition, *NF1* genetic alterations were more frequent in metastatic ILCs than in primary ILCs, in agreement with Sokol et al.^37^, who reported on the presence of genetic alterations targeting *NF1* arising in the setting of relapse on endocrine therapy, indicating that these alterations likely constitute a mechanism of endocrine resistance^37^. In our study, although the number of *NF1*-mutant metastatic ILCs was insufficient for a formal mutual exclusivity analysis with *ESR1* ligand-binding domain mutations, we observed that none of the metastatic ILCs with *NF1* biallelic inactivation harbored *ESR1* mutations. These findings provide further evidence supporting the notion that *NF1* mutations may constitute a mechanism of resistance to endocrine therapy^37^.

We observed a higher mutational burden in metastatic ILCs than in primary ILCs and in age-, menopausal status-, and ER/HER2 status-matched metastatic IDC-NSTs, in agreement with the study by Sokol et al.^37^. Consistent with previous studies reporting that metastatic ER-positive BCs in general have an enrichment for the APOBEC mutagenesis process^7,10^, we observed an enrichment in APOBEC mutational signatures in metastatic ILCs as compared to primary ILCs and metastatic IDC-NSTs matched to the metastatic ILCs according to clinicopathologic features. Of note, APOBEC genes are not part of the MSK-IMPACT panel, and genetic alterations affecting APOBEC genes were not investigated. APOBEC processes have been implicated in tumor hypermutation^44^ and likely play a role in resistance to endocrine therapy. Further studies to determine the role of APOBEC signatures in the clinical behavior of metastatic ILCs and the potential utility of the detection of APOBEC mutagenesis, as a biomarker of resistance to endocrine therapy are warranted.

Our study has limitations, including the small sample size of the metastatic mixed mucinous, micropapillary, and metaplastic BCs, owing to their rarity, which may limit the identification of statistically significant differences in the comparisons performed. Hence, the negative conclusions related to these histologic special types need to be interpreted with caution, as we cannot rule out type II or β errors. Furthermore, our study is based on the reanalysis of targeted sequencing data, and we cannot rule out differences between primary and metastatic special histologic types of BC outside of the genes captured by MSK-IMPACT. Hence, whole-exome and/or whole-genome analyses of metastatic special types of BCs are warranted. Moreover, the primary and metastatic special histologic subtypes of BC were not matched lesions from the same patients.

Notwithstanding these limitations, our study indicates that the repertoire of genetic alterations in primary and metastatic forms of special histologic types of BC is remarkably similar; however, key differences exist, such as higher mutational burden and an enrichment for the APOBEC mutational processes in metastatic ILCs. Our findings also suggest that *ERBB2* and *ESR1* mutations should be considered as potential mechanisms of resistance to endocrine therapy and druggable targets in clinically HER2-negative metastatic ILCs.

## Methods

### Cases and study population

The study was approved by Memorial Sloan Kettering Cancer Center Institutional Review Board as part of the project whose findings were initially published by Razavi et al.^11^. Informed consent was provided in the original study by Razavi et al.^11^. Targeted massively parallel sequencing data of primary and metastatic BCs were obtained from the study by Razavi et al.^11^. All cases had been previously subjected to targeted capture massively parallel sequencing using the MSK-IMPACT sequencing assay from the study by Razavi et al.^11^ (Supplementary Table 2). Following the criteria put forward by the WHO^3^, 309 BCs were classified as of one of the special histologic types included in this study: 259 were classified as classic ILCs (*n* = 127 metastatic and *n* = 132 primary), 19 as mixed (i.e., >50% but <90% mucinous component) mucinous carcinomas (*n* = 5 metastatic and *n* = 14 primary), 20 as pure micropapillary carcinomas (*n* = 12 metastatic and *n* = 8 primary), and 11 as metaplastic BCs (*n* = 6 metastatic and *n* = 5 primary, Supplementary Table 2). The initial diagnosis of a given special histologic type of BC was retrieved from Razavi et al.^11^, and cases for which the histologic material of the sample subjected to sequencing was available (*n* = 265) were reviewed centrally by a board-certified breast pathologist (F.P.) for diagnosis confirmation. Pleomorphic ILCs (metastatic, *n* = 6; primary, *n* = 8) were excluded from further analyses. ER and HER2 status had been assessed by immunohistochemistry and/or FISH, as previously described^11^, following the American Society of Clinical Oncology/College of American Pathologists guidelines^45,46^.

### Comparison with common forms of breast cancer

For the comparison of non-synonymous TMB, FGA, frequency of non-synonymous somatic mutations, and CNAs, metastatic BCs of special histologic subtype were compared to those of IDC-NSTs included in the same study^11^, matched by age (20-year intervals), menopausal status, and ER/HER2 status and to those of their primary counterparts. Metastatic ILCs were matched to metastatic IDC-NSTs from the study by Razavi et al.^11^ previously subjected to MSK-IMPACT at a 1:2 ratio, whereas mixed mucinous BCs, micropapillary BCs, and metaplastic BCs were matched to IDC-NSTs at a 1:3 ratio. No statistically significant differences were observed in the therapy received prior to tumor sampling between the metastatic BCs of special histologic types and metastatic IDC-NSTs matched by clinicopathologic characteristics in the cohorts analyzed in this study (Supplementary Table 6). Lollipop plots were produced using MutationMapper on cBioPortal^47^ (http://www.cbioportal.org), manually curated and mutation types were color-coded as follows: splice-site SNV (yellow), missense SNV (green), truncating SNV (black), in-frame insertion/deletion (brown), and hotspot mutation (orange).

### Targeted massively parallel sequencing analysis

All samples included in this study were subjected to targeted sequencing using the FDA-approved MSK-IMPACT assay^48^, as part of the study by Razavi et al.^11^. Non-synonymous somatic mutations, amplifications, and homozygous deletions were retrieved from the original study^11^. The raw MSK-IMPACT sequencing data (i.e., FASTQ files) were reprocessed using our validated bioinformatics pipeline, as previously described^49,50^, for the inference of copy number gains and losses, and loss of heterozygosity of genes targeted by somatic mutations and mutational signatures. Mutations affecting hotspot codons were annotated as described. Non-synonymous TMB was calculated as the number of non-synonymous mutations divided by the total genomic region assessed by MSK-IMPACT, per megabase. The FGA, defined as the number of base pairs which are not copy neutral divided by the size of genome assayed, was retrieved from the original study by Razavi et al.^11^. Mutational signatures were defined using SigMA^34^ using all synonymous and non-synonymous somatic mutations of cases with at least five SNVs, as previously reported^35^. Tumor purity was inferred using FACETS^51^. The median tumor purity of special histologic type BCs analyzed in study was 0.43 (95% CI = 0.30–0.87). Of note, the tumor purity of metastatic BCs of special histologic type (median = 0.47; 95% CI = 0.30–0.88) was higher than that of primary tumors (median = 0.39; 95% CI = 0.27–0.86; *P* = 1.4 × 10^−2^). As expected, the tumor purity of metastatic ILCs (median = 0.48; 95% CI = 0.30–0.88) was higher than that of primary ILCs (median = 0.38; 95% CI = 0.28–0.86; *P* = 1.1 × 10^−3^), whereas no differences were observed in the comparisons between metastatic and primary BCs of other histologic types analyzed in this study.

### Assessment of TILs infiltration

Histologic assessment of TILs infiltration in primary and metastatic ILCs with a sufficient number of SNVs (≥5) for accurate assessment of mutational signatures by SigMA, and available hematoxylin and eosin (H&E) slides was performed. The assessment of TILs infiltration was conducted following the guidelines described by the International TIL working group^52^. In brief, following the examination of one representative section, the intratumoral stromal area covered by mononuclear cells, including lymphocytes and plasma cells, was recorded.

### Immunohistochemistry

Immunohistochemical analyses for MLH1, MSH2, MSH6, and PMS2 were performed in a Bond-3 automated stainer platform (Leica Biosystems, Wetzlar, Germany). In brief, following antigen retrieval (ER2, Leica) for 30–40 min, tissue sections were incubated with monoclonal antibodies against MLH1 (clone ES05; Leica Biosystems; dilution 1:500), MSH2 (clone G219–1129; Cell Marque, Rocklin, CA; dilution 1:750), MSH6 (clone EP49; Dako, Glostrup, Denmark; dilution 1:500), or PMS2 (clone A16.4; BD Biosciences, Franklin Lakes, NJ; dilution 1:500) for 30 min. A polymer-based kit was employed as secondary reagent (Leica Biosystems). Assessment of the MLH2, MSH2, MSH6, and PMS2 expression was conducted by a board-certified pathologist (F.P.) following the current standard practice.

### Statistical analysis

Statistical analyses were conducted using R v3.1.2. Fisher’s exact tests were employed for comparisons between categorical variables, and Mann–Whitney *U* test were used for continuous variables. All tests were two-sided and *P* values < 0.05 were considered statistically significant. We performed multiple testing correction using the Benjamini–Hochberg procedure to control for the false discovery rate (*q* values; Supplementary Table 3). To assess the mutual exclusivity between *ERBB2* and *ESR1* mutations (hotspot mutations and non-hotspot pathogenic mutations) in ER-positive metastatic ILC and IDC-NST using CoMEt^53^.

### Reporting summary

Further information on experimental design is available in the Nature Research Reporting Summary linked to this paper.

## Supplementary information

Supplementary materials

Reporting Summary Checklist FLAT

## Data Availability

The data generated and analyzed during this study are described in the following data record: 10.6084/m9.figshare.12855149^54^. Histologic images supporting Figs. 1–4, and Supplementary Fig. 7 are not publicly available, but can be requested from the corresponding author, F.P. MSK-IMPACT sequencing data supporting Figs. 1–5, Supplementary Figs. 1–6, and Supplementary Tables 3–5 are publicly available in cBioPortal at the following accession: https://identifiers.org/cbioportal:breast_msk_2018^55^. Clinical data supporting Supplementary Tables 1, 2, 5 and 6 are available in the original publication by Razavi et al.^11^.
