# Supplementary material for: The genomic landscape of metastatic histologic special types of invasive breast cancer
Source: NPJ Breast Cancer. 2020 Oct 14;6:53. doi: 10.1038/s41523-020-00195-4 (PMC7560857; doi:10.1038/s41523-020-00195-4)
Supplement: Supplementary file 1 — Supplementary materials [file 41523_2020_195_MOESM1_ESM.pdf]

SUPPLEMENTARY MATERIALS

The genomic landscape of metastatic histologic special types of invasive breast cancer

Pareja et al.

Supplementary Figures 1-7

Supplementary Tables 1-6

Supplementary Figure 1

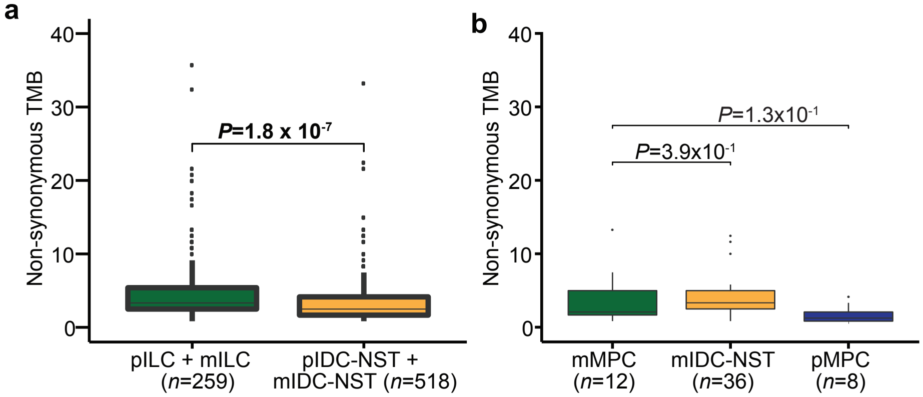

Supplementary Figure 1. Non-synonymous tumor mutation burden in primary and metastatic breast cancers of special histologic subtypes

(**a-b**) Boxplots depicting (**a**) the non-synonymous tumor mutation burden (TMB) in combined primary and metastatic invasive lobular carcinomas (pILC + mILC, n=259), compared to combined primary and metastatic invasive ductal carcinomas of no special type matched by age, menopausal status, estrogen receptor (ER)/HER2 status and sample type (pIDC-NST + mIDC-NST; n=518) and (**e**) the non-synonymous TMB metastatic micropapillary carcinomas (mMPC, n=12), metastatic IDC-NSTs matched by clinicopathologic features (mIDC-NST; n=36) and primary micropapillary carcinomas (pMPC; n=8). Mann-Whitney *U* test, two-sided

Supplementary Figure 2

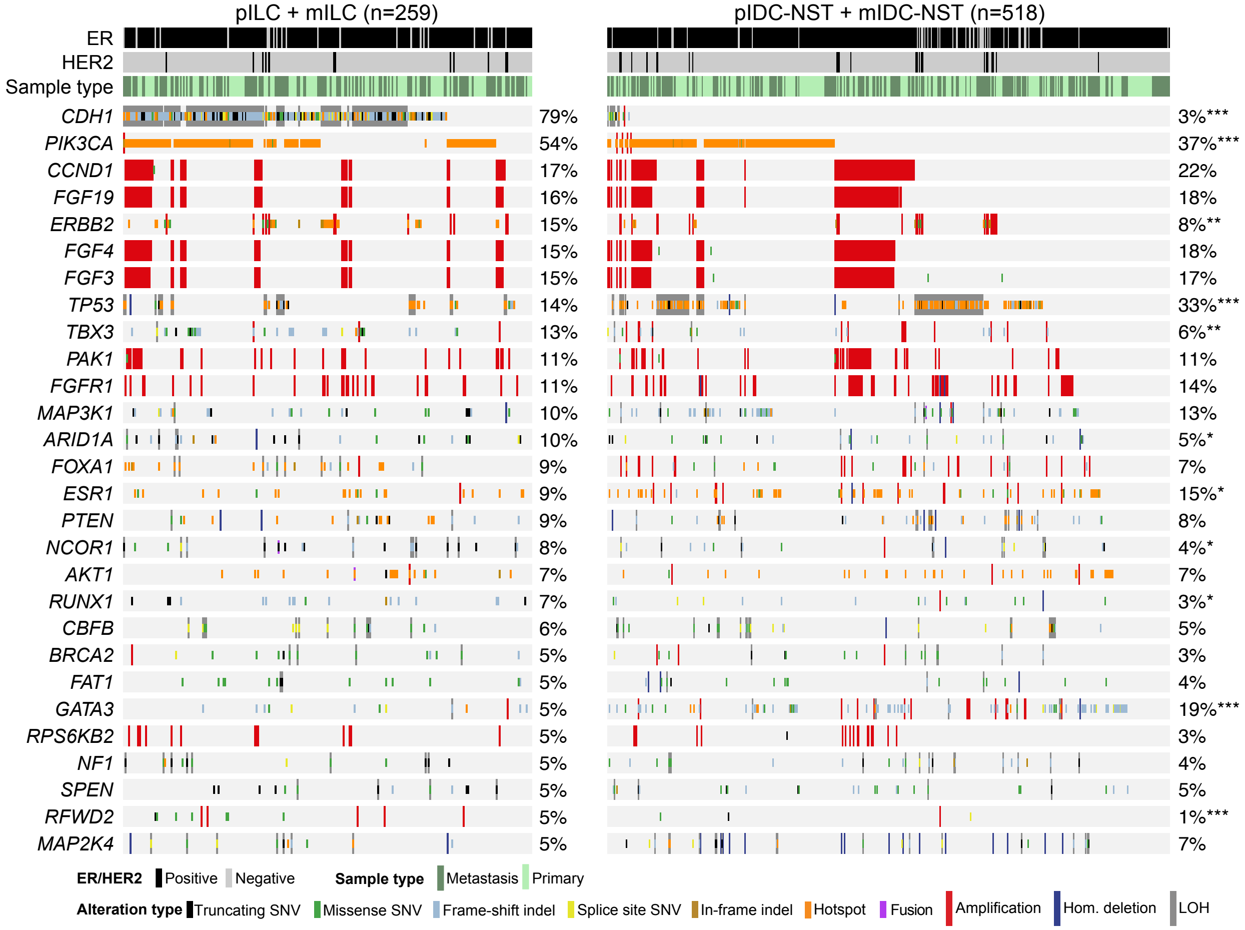

Supplementary Figure 2. Repertoire of somatic genetic alterations affecting key cancer genes in combined primary and metastatic invasive lobular carcinomas and in matched combined primary and metastatic invasive ductal carcinomas of no special type. Comparison of the key cancer genes most frequently affected by non-synonymous somatic mutations, amplifications or homozygous deletions in combined primary and metastatic invasive lobular carcinomas (pILC + mILC; n=259), and in metastatic invasive ductal carcinomas of no special type matched by clinicopathologic characteristics ad sample type (pIDC-NST + mIDC-NST; n=518). Cases are shown in columns and genes in rows. Mutation types are color-coded according to the legend. ER/HER2 status are shown in the respective phenobars (top). *, *P*<0.05, **, *P*<0.01, ***, *P*<0.001; Fisher's exact test, two-tailed.

Supplementary Figure 3

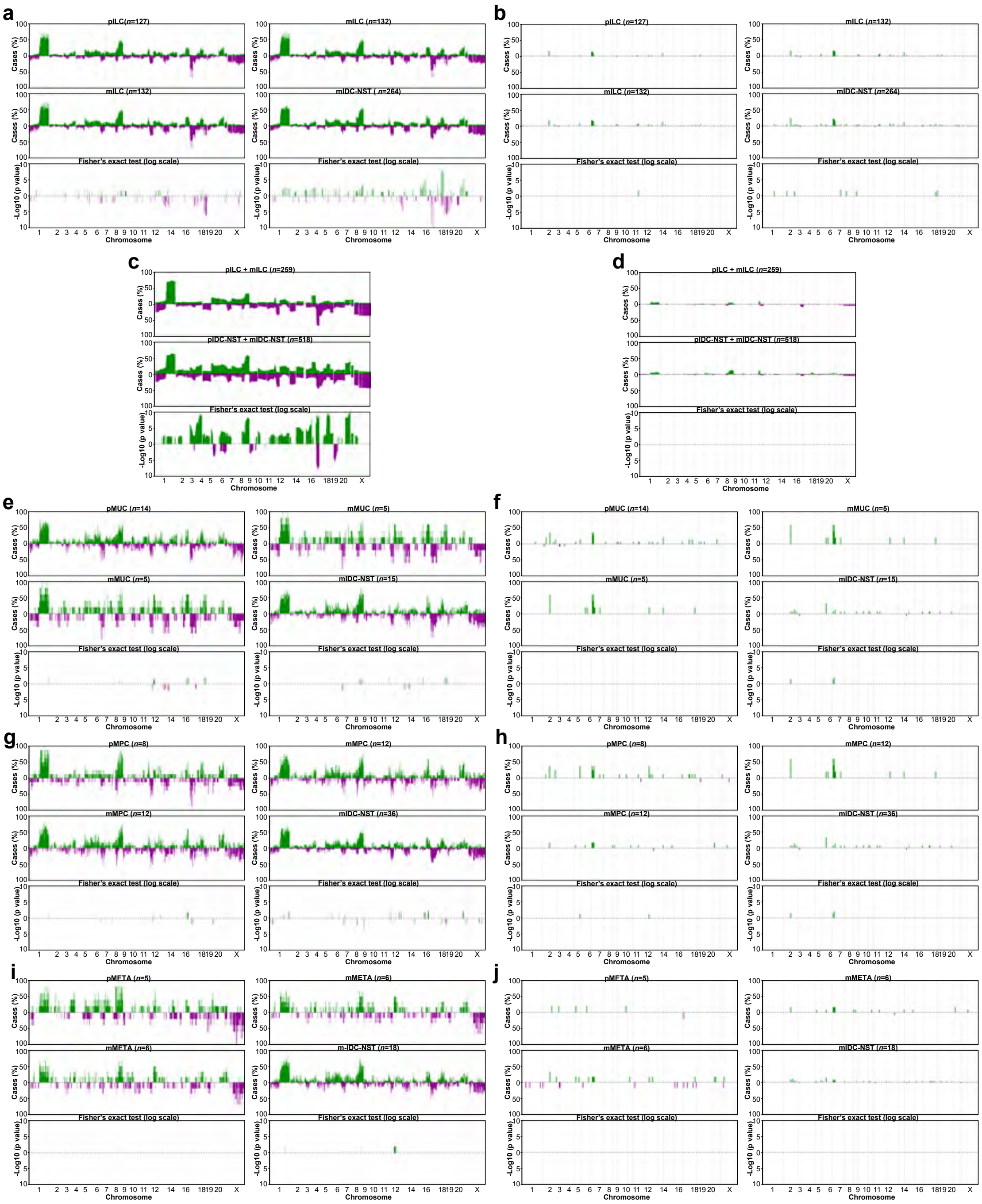

Supplementary Figure 3. Frequency of copy number alterations in primary and metastatic special histologic subtypes of breast cancer.

Frequency plots and Fisher's exact test comparisons corrected for multiple testing of copy number (**a**) gains and losses and (**b**) amplifications and homozygous deletions between metastatic lobular

carcinomas (mILC; n=132) compared to primary invasive lobular carcinomas (pILC; n=127; left), and to metastatic invasive ductal carcinomas of no special type (mIDC-NST) matched by age, menopausal status and estrogen receptor (ER)/HER2 status (n=264; right). (**c**) Gains and losses and (**d**) amplifications and homozygous deletions in combined primary and metastatic lobular carcinomas (pILC + mILC; n=259) and primary and metastatic IDC-NSTs matched by clinicopathologic characteristics and sample type (pIDC-NST + mIDC-NST; n=518). (**e**) Gains and losses and (**f**) amplifications and homozygous deletions between metastatic mixed mucinous breast cancers (mMUC; n=5) compared to primary mixed mucinous breast cancers (pMUC; n=14, left), and to metastatic IDC-NSTs matched by clinicopathologic characteristics (mIDC-NST; n=15, right). (**g**) Gains and losses and **(h**) amplifications and homozygous deletions of metastatic micropapillary breast cancers (mMPC; n=12) compared to primary micropapillary breast cancers (pMPC; n=8, left), and to metastatic IDC-NST matched by clinicopathologic features (mIDC-NST; n=36, right). (**i**) Gains and losses and (**j**) amplifications and homozygous deletions of metastatic metaplastic BCs (mMETA; n=6) compared to primary metaplastic BCs (pMETA; n=5, left), and to metastatic IDC-NST matched by clinicopathologic characteristics (mIDC-NST; n=18, right). Frequency of gains/amplifications (green) or losses/homozygous deletions (purple) is shown for each gene (y-axis), according to genomic position (x-axis). Inverse Log10 values of the two-sided Fisher's exact test *P*-values are plotted according to genomic location (lower panels).

Supplementary Figure 4

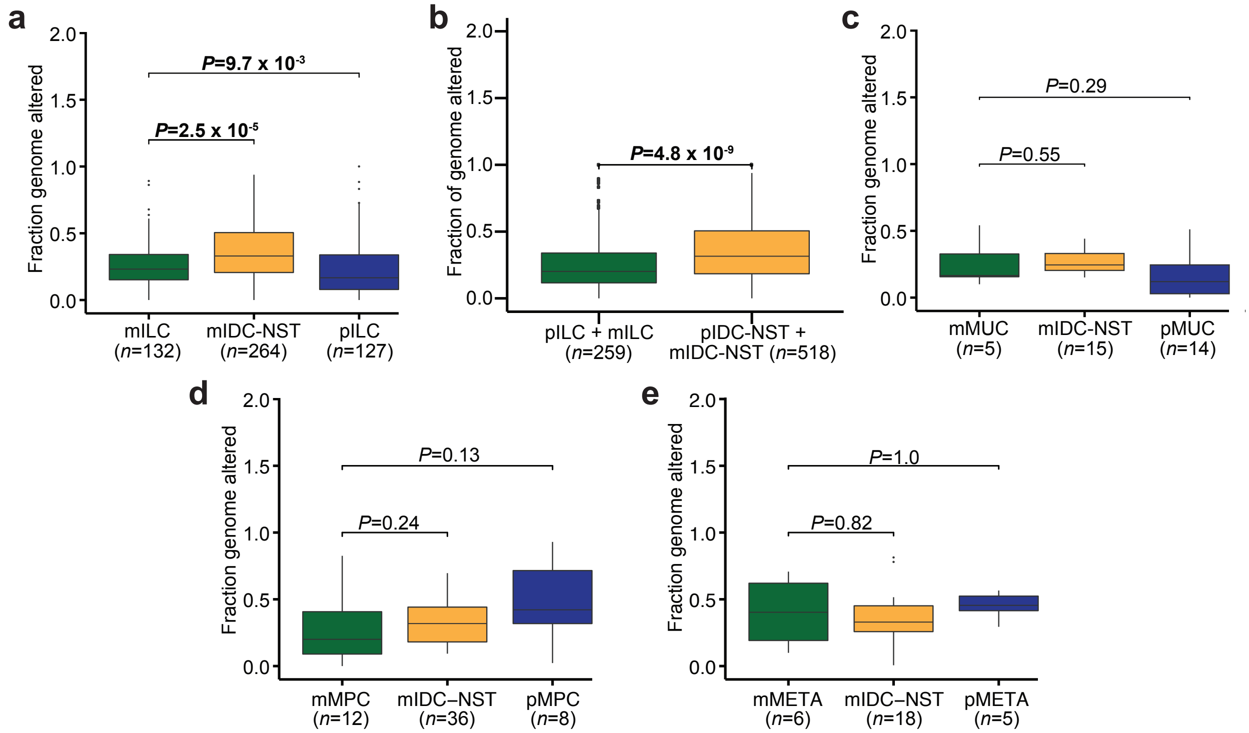

Supplementary Figure 4. Fraction of genome altered in primary and metastatic special histologic subtypes of breast cancer.

Boxplots depicting the fraction of genome altered (FGA) of (**a**) metastatic lobular carcinomas (mILC; n=132), metastatic invasive ductal carcinomas of no special type matched by clinicopathologic characteristics (mIDC-NST; n=264) and primary invasive lobular carcinomas (pILC; n=127), (**b**) combined primary and metastatic lobular carcinomas (pILC + mILC; n=259), with age-, menopausal status-, ER/HER2 status- and sample type-matched combined primary and metastatic IDC-NSTs (pIDC-NST + mIDC-NST; n=518), (**c**) metastatic mixed mucinous breast cancers (mMUC; n=5), mIDC-NSTs matched by clinicopathologic features (n=15) and primary mixed mucinous carcinomas (pMUC; n=14), (**d**) metastatic micropapillary breast cancers (mMPC; n=12), mIDC-NST matched by clinicopathologic characteristics (n=36) and primary micropapillary breast cancers (pMPC; n=8), and (**e**) metastatic metaplastic breast cancers (mMETAs; n=6), mIDC-NSTs matched by clinicopathologic features (n=18) and primary

metaplastic breast cancers (pMETA; n=5). Statistically significant *P* values (*P*<0.05) are bolded. Mann-Whitney *U* test, two-sided.

Supplementary Figure 5

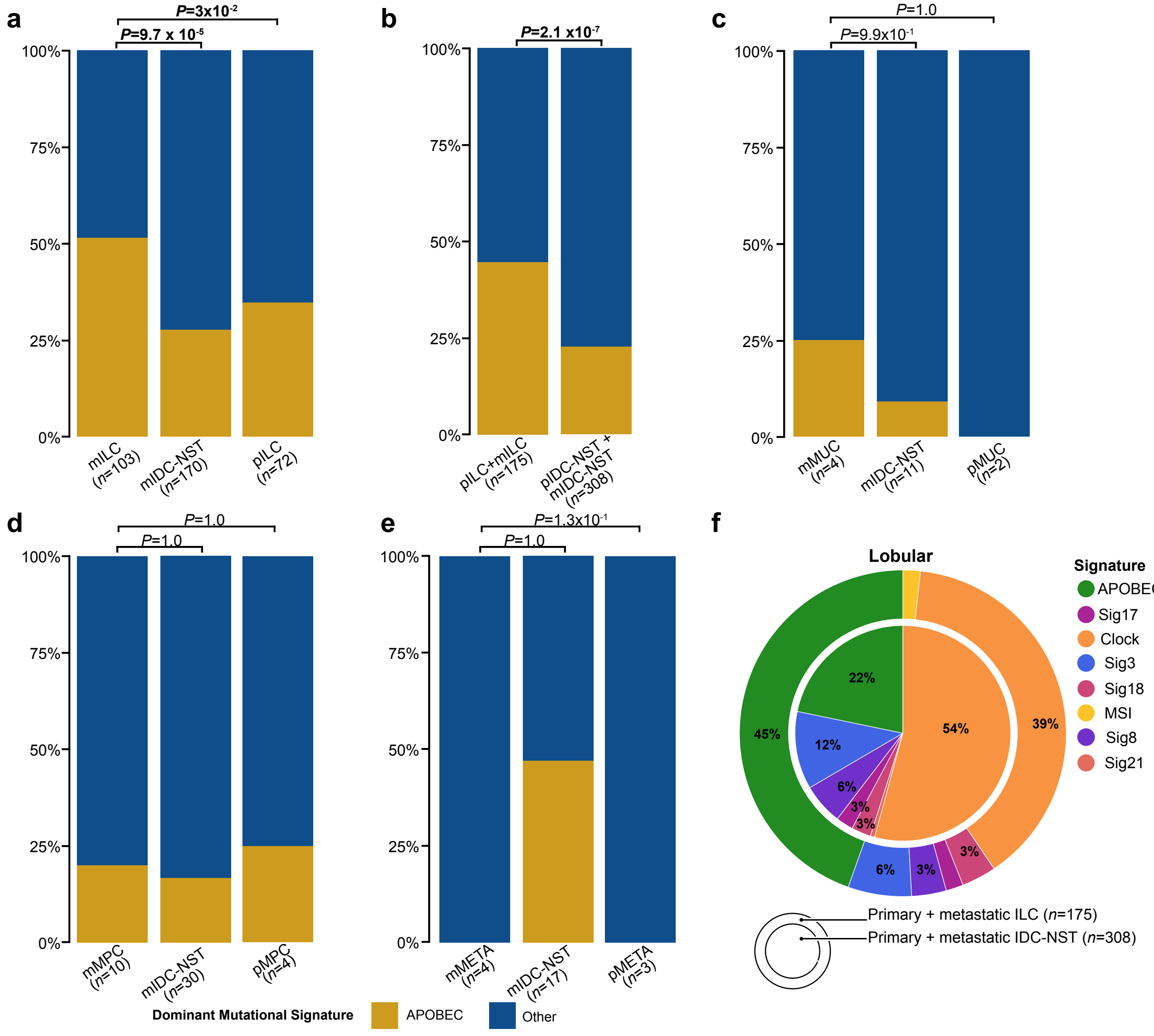

Supplementary Figure 5. Proportion of primary and metastatic breast cancers of special histologic types with a dominant APOBEC mutational signature, and mutational signatures in primary and metastatic lobular carcinomas.

Stacked bar plots displaying the proportion of cases with a dominant APOBEC mutational signature in (**a**) metastatic invasive lobular carcinomas (mILC; n=103), metastatic invasive ductal carcinomas of no special type matched by age, menopausal status and estrogen receptor (ER)/HER2 status (mIDC-NST; n=170), and primary invasive lobular carcinomas (pILC; n=72), (**b**) in combined primary and metastatic lobular carcinomas (pILC + mILC; n=175) and primary and metastatic invasive ductal carcinomas of no special type matched by clinicopathologic

characteristics and sample type (pIDC-NST + mIDC-NST; n=308), (**c**) in metastatic mixed mucinous breast cancers (mMUC; n=4), mIDC-NSTs matched by clinicopathologic features (n=11) and primary mixed mucinous breast cancers (pMUC; n=2), (**d**) in metastatic micropapillary breast cancers (mMPC; n=10), mIDC-NSTs matched by clinicopathologic characteristics (n=30) and primary micropapillary breast cancers (pMPC; n=4), and in (**e**) metastatic metaplastic breast cancers (mMETA; n=4), mIDC-NSTs matched by clinicopathologic features (n=17) and primary metaplastic breast cancers (pMETA; n=3). Two-tailed fisher's exact test was performed. (**f**) Proportion of dominant mutational signatures in pILC + mILC (n=175) and pIDC-NST + mIDC-NST (n=308).

Supplementary Figure 6

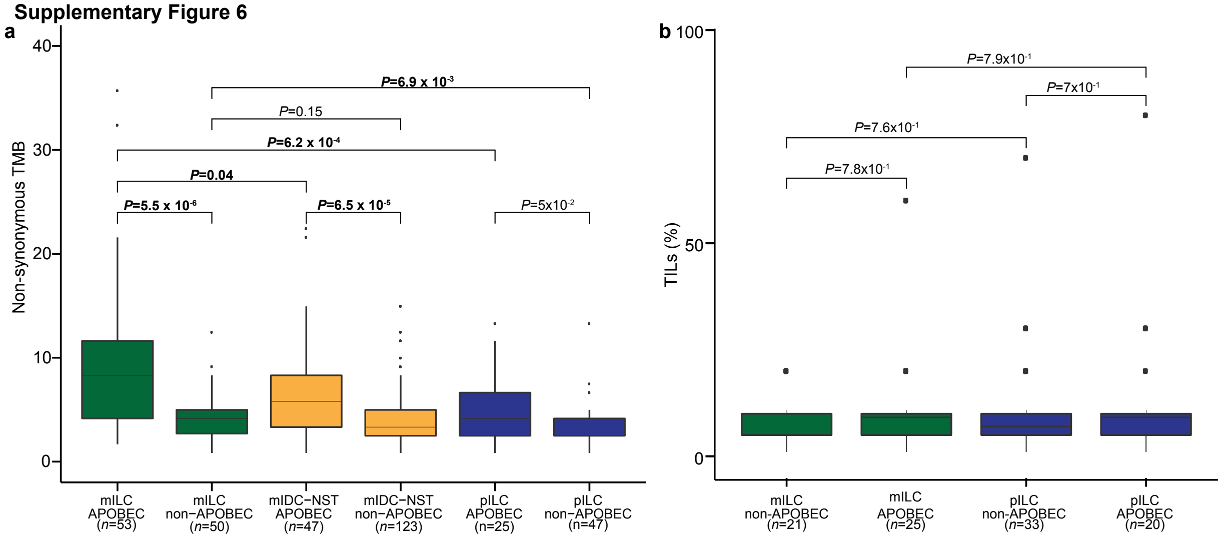

Supplementary Figure 6. Non-synonymous tumor mutation burden and extent of tumor infiltrating lymphocytes in primary and metastatic lobular carcinomas in relation to a dominant APOBEC mutagenesis signature.

(**a**) Boxplots depicting the non-synonymous tumor mutation burden (TMB) in metastatic lobular carcinomas (mILC) displaying a dominant APOBEC (n=53) or a non-APOBEC dominant mutational signature (n=50), age-, menopausal status- and estrogen receptor (ER)/HER2 status-matched metastatic invasive ductal carcinomas of no special type (mIDC-NSTs) with a dominant APOBEC (n=47) or a non-APOBEC mutational signature (n=123) and primary invasive lobular carcinomas (pILC) with a dominant APOBEC (n=25) and a non-APOBEC dominant signature (n=47). (**b**) Boxplots depicting the extent of infiltration by tumor infiltrating lymphocytes (TILs) in mILCs with a dominant mutational signature other than APOBEC (n=21), mILCs with a dominant APOBEC mutational signature (n=25), pILCs with a dominant mutational signature other than APOBEC (n=33) and pILCs with a dominant APOBEC mutational signature (n=20). Statistically significant *P* values ($P<0.05$) are bolded. Mann-Whitney *U* test, two-sided.

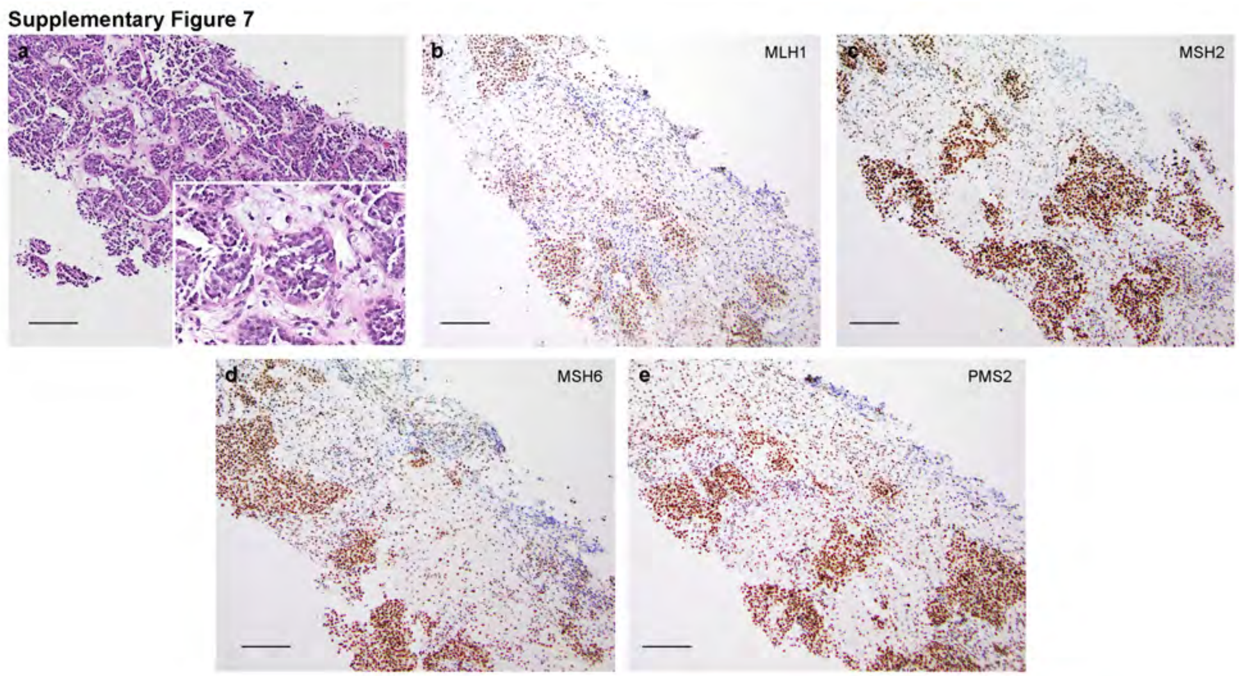

Supplementary Fig 7: Expression of mismatch repair proteins in a metastatic mixed mucinous carcinoma. Representative (**A**) hematoxylin and eosin micrographs, and (**B**) MLH1, (**C**) MSH2, (**D**) MSH6 and (**E**) PMS2 expression assessed by immunohistochemistry in case MSK-MUC-4. Scale bars, 100 μm

Supplementary Table 1. Estrogen receptor and HER2 status of the breast cancers of special histologic subtype included in this study

Estrogen receptor (ER)/ HER2 status	Invasive lobular carcinoma			Mixed mucinous breast cancer			Micropapillary breast cancer			Metaplastic breast cancer		
	Primary (n=127)	Metastasis (n=132)	Fisher's exact test *P* value	Primary (n=14)	Metastasis (n=5)	Fisher's exact test *P* value	Primary (n=8)	Metastasis (n=12)	Fisher's exact test *P* value	Primary (n=5)	Metastasis (n=6)	Fisher's exact test *P* value
ER+/HER2-	121 (95.3%)	107 (81.1%)	0.0016	11 (57.9%)	5 (100%)	0.53	5 (62.5%)	9 (75%)	0.339	1 (20%)	1 (16.7%)	1
ER+/HER2+	3 (2.4%)	6 (4.5%)		3 (15.8%)	0 (0%)		2 (25%)	1 (8.3%)		0 (0%)	0 (0%)	
ER-/HER2-	3 (2.4%)	16 (12.1%)		0 (0%)	0 (0%)		0 (0%)	2 (16.7%)		4 (80%)	5 (83.3%)	
ER-/HER2+	0 (0%)	3 (2.3%)		0 (0%)	0 (0%)		1 (12.5%)	0 (0%)		0 (0%)	0 (0%)	

Supplementary Table 2. Clinicopathologic characteristics of the 309 breast cancers of histologic special type in this study and sequencing platform used

Sample ID	Age at diagnosis	Histology	Sample type	Estrogen receptor (ER)/HER2 status	MSK-IMPACT Version
MSK-ILC-1	52	Invasive Lobular Carcinoma	Metastasis	ER+/HER2-	MSK-IMPACT-341
MSK-ILC-2	79	Invasive Lobular Carcinoma	Metastasis	ER+/HER2-	MSK-IMPACT-468
MSK-ILC-3	54	Invasive Lobular Carcinoma	Metastasis	ER+/HER2-	MSK-IMPACT-468
MSK-ILC-4	38	Invasive Lobular Carcinoma	Metastasis	ER+/HER2-	MSK-IMPACT-410
MSK-ILC-5	58	Invasive Lobular Carcinoma	Metastasis	ER+/HER2-	MSK-IMPACT-410
MSK-ILC-6	46	Invasive Lobular Carcinoma	Metastasis	ER+/HER2-	MSK-IMPACT-341
MSK-ILC-7	62	Invasive Lobular Carcinoma	Metastasis	ER+/HER2-	MSK-IMPACT-410
MSK-ILC-8	45	Invasive Lobular Carcinoma	Metastasis	ER+/HER2-	MSK-IMPACT-341
MSK-ILC-9	51	Invasive Lobular Carcinoma	Metastasis	ER-/HER2-	MSK-IMPACT-341
MSK-ILC-10	38	Invasive Lobular Carcinoma	Metastasis	ER+/HER2+	MSK-IMPACT-410
MSK-ILC-11	38	Invasive Lobular Carcinoma	Metastasis	ER+/HER2-	MSK-IMPACT-410
MSK-ILC-12	65	Invasive Lobular Carcinoma	Metastasis	ER-/HER2-	MSK-IMPACT-410
MSK-ILC-13	66	Invasive Lobular Carcinoma	Primary	ER+/HER2-	MSK-IMPACT-410
MSK-ILC-14	47	Invasive Lobular Carcinoma	Primary	ER+/HER2-	MSK-IMPACT-468
MSK-ILC-15	63	Invasive Lobular Carcinoma	Metastasis	ER+/HER2-	MSK-IMPACT-468
MSK-ILC-16	59	Invasive Lobular Carcinoma	Primary	ER+/HER2-	MSK-IMPACT-468
MSK-ILC-17	42	Invasive Lobular Carcinoma	Metastasis	ER+/HER2-	MSK-IMPACT-410
MSK-ILC-18	47	Invasive Lobular Carcinoma	Metastasis	ER+/HER2-	MSK-IMPACT-341
MSK-ILC-19	31	Invasive Lobular Carcinoma	Metastasis	ER+/HER2-	MSK-IMPACT-410
MSK-ILC-20	60	Invasive Lobular Carcinoma	Metastasis	ER+/HER2-	MSK-IMPACT-410
MSK-ILC-21	66	Invasive Lobular Carcinoma	Primary	ER+/HER2-	MSK-IMPACT-468
MSK-ILC-22	43	Invasive Lobular Carcinoma	Metastasis	ER-/HER2-	MSK-IMPACT-468
MSK-ILC-23	64	Invasive Lobular Carcinoma	Metastasis	ER+/HER2-	MSK-IMPACT-410
MSK-ILC-24	46	Invasive Lobular Carcinoma	Metastasis	ER+/HER2-	MSK-IMPACT-341
MSK-ILC-25	58	Invasive Lobular Carcinoma	Metastasis	ER+/HER2-	MSK-IMPACT-410
MSK-ILC-26	55	Invasive Lobular Carcinoma	Primary	ER+/HER2-	MSK-IMPACT-468
MSK-ILC-27	34	Invasive Lobular Carcinoma	Metastasis	ER+/HER2-	MSK-IMPACT-341
MSK-ILC-28	47	Invasive Lobular Carcinoma	Metastasis	ER+/HER2-	MSK-IMPACT-341
MSK-ILC-29	76	Invasive Lobular Carcinoma	Primary	ER+/HER2-	MSK-IMPACT-468
MSK-ILC-30	66	Invasive Lobular Carcinoma	Metastasis	ER+/HER2-	MSK-IMPACT-410
MSK-ILC-31	78	Invasive Lobular Carcinoma	Primary	ER+/HER2-	MSK-IMPACT-410
MSK-ILC-32	52	Invasive Lobular Carcinoma	Metastasis	ER+/HER2-	MSK-IMPACT-410
MSK-ILC-33	69	Invasive Lobular Carcinoma	Primary	ER+/HER2-	MSK-IMPACT-468
MSK-ILC-34	50	Invasive Lobular Carcinoma	Primary	ER+/HER2-	MSK-IMPACT-468
MSK-ILC-35	66	Invasive Lobular Carcinoma	Primary	ER+/HER2-	MSK-IMPACT-410
MSK-ILC-36	76	Invasive Lobular Carcinoma	Primary	ER+/HER2-	MSK-IMPACT-468
MSK-ILC-37	36	Invasive Lobular Carcinoma	Metastasis	ER-/HER2+	MSK-IMPACT-341
MSK-ILC-38	72	Invasive Lobular Carcinoma	Metastasis	ER+/HER2-	MSK-IMPACT-341
MSK-ILC-39	42	Invasive Lobular Carcinoma	Metastasis	ER-/HER2-	MSK-IMPACT-341
MSK-ILC-40	55	Invasive Lobular Carcinoma	Metastasis	ER+/HER2+	MSK-IMPACT-410
MSK-ILC-41	46	Invasive Lobular Carcinoma	Metastasis	ER+/HER2-	MSK-IMPACT-410
MSK-ILC-42	60	Invasive Lobular Carcinoma	Metastasis	ER+/HER2-	MSK-IMPACT-341
MSK-ILC-43	60	Invasive Lobular Carcinoma	Metastasis	ER+/HER2-	MSK-IMPACT-341
MSK-ILC-44	53	Invasive Lobular Carcinoma	Metastasis	ER+/HER2-	MSK-IMPACT-410
MSK-ILC-45	57	Invasive Lobular Carcinoma	Primary	ER+/HER2-	MSK-IMPACT-341
MSK-ILC-46	52	Invasive Lobular Carcinoma	Primary	ER+/HER2-	MSK-IMPACT-410
MSK-ILC-47	55	Invasive Lobular Carcinoma	Metastasis	ER+/HER2-	MSK-IMPACT-341
MSK-ILC-48	47	Invasive Lobular Carcinoma	Metastasis	ER+/HER2-	MSK-IMPACT-410
MSK-ILC-49	60	Invasive Lobular Carcinoma	Metastasis	ER+/HER2-	MSK-IMPACT-410
MSK-ILC-50	54	Invasive Lobular Carcinoma	Metastasis	ER+/HER2-	MSK-IMPACT-410
MSK-ILC-51	41	Invasive Lobular Carcinoma	Metastasis	ER+/HER2-	MSK-IMPACT-410
MSK-ILC-52	60	Invasive Lobular Carcinoma	Metastasis	ER+/HER2-	MSK-IMPACT-410
MSK-ILC-53	41	Invasive Lobular Carcinoma	Metastasis	ER-/HER2-	MSK-IMPACT-410
MSK-ILC-54	76	Invasive Lobular Carcinoma	Metastasis	ER+/HER2-	MSK-IMPACT-410
MSK-ILC-55	50	Invasive Lobular Carcinoma	Metastasis	ER+/HER2-	MSK-IMPACT-410

MSK-ILC-56	62	Invasive Lobular Carcinoma	Primary	ER+/HER2-	MSK-IMPACT-410
MSK-ILC-57	53	Invasive Lobular Carcinoma	Metastasis	ER-/HER2+	MSK-IMPACT-410
MSK-ILC-58	54	Invasive Lobular Carcinoma	Primary	ER+/HER2-	MSK-IMPACT-410
MSK-ILC-59	44	Invasive Lobular Carcinoma	Metastasis	ER+/HER2-	MSK-IMPACT-410
MSK-ILC-60	76	Invasive Lobular Carcinoma	Primary	ER+/HER2-	MSK-IMPACT-341
MSK-ILC-61	67	Invasive Lobular Carcinoma	Primary	ER+/HER2+	MSK-IMPACT-468
MSK-ILC-62	47	Invasive Lobular Carcinoma	Metastasis	ER+/HER2-	MSK-IMPACT-410
MSK-ILC-63	53	Invasive Lobular Carcinoma	Metastasis	ER+/HER2-	MSK-IMPACT-341
MSK-ILC-64	58	Invasive Lobular Carcinoma	Metastasis	ER-/HER2-	MSK-IMPACT-410
MSK-ILC-65	46	Invasive Lobular Carcinoma	Metastasis	ER+/HER2-	MSK-IMPACT-410
MSK-ILC-66	56	Invasive Lobular Carcinoma	Metastasis	ER+/HER2-	MSK-IMPACT-410
MSK-ILC-67	50	Invasive Lobular Carcinoma	Metastasis	ER+/HER2-	MSK-IMPACT-410
MSK-ILC-68	71	Invasive Lobular Carcinoma	Metastasis	ER+/HER2-	MSK-IMPACT-341
MSK-ILC-69	71	Invasive Lobular Carcinoma	Primary	ER+/HER2-	MSK-IMPACT-341
MSK-ILC-70	60	Invasive Lobular Carcinoma	Primary	ER+/HER2+	MSK-IMPACT-410
MSK-ILC-71	42	Invasive Lobular Carcinoma	Primary	ER+/HER2-	MSK-IMPACT-341
MSK-ILC-72	54	Invasive Lobular Carcinoma	Metastasis	ER+/HER2-	MSK-IMPACT-341
MSK-ILC-73	54	Invasive Lobular Carcinoma	Metastasis	ER+/HER2-	MSK-IMPACT-410
MSK-ILC-74	51	Invasive Lobular Carcinoma	Metastasis	ER+/HER2-	MSK-IMPACT-341
MSK-ILC-75	62	Invasive Lobular Carcinoma	Metastasis	ER+/HER2-	MSK-IMPACT-410
MSK-ILC-76	43	Invasive Lobular Carcinoma	Metastasis	ER+/HER2-	MSK-IMPACT-410
MSK-ILC-77	66	Invasive Lobular Carcinoma	Metastasis	ER+/HER2-	MSK-IMPACT-341
MSK-ILC-78	57	Invasive Lobular Carcinoma	Primary	ER+/HER2-	MSK-IMPACT-410
MSK-ILC-79	42	Invasive Lobular Carcinoma	Primary	ER+/HER2-	MSK-IMPACT-341
MSK-ILC-80	50	Invasive Lobular Carcinoma	Metastasis	ER+/HER2-	MSK-IMPACT-468
MSK-ILC-81	43	Invasive Lobular Carcinoma	Primary	ER+/HER2-	MSK-IMPACT-410
MSK-ILC-82	66	Invasive Lobular Carcinoma	Metastasis	ER+/HER2-	MSK-IMPACT-341
MSK-ILC-83	53	Invasive Lobular Carcinoma	Primary	ER+/HER2-	MSK-IMPACT-410
MSK-ILC-84	55	Invasive Lobular Carcinoma	Metastasis	ER+/HER2-	MSK-IMPACT-341
MSK-ILC-85	58	Invasive Lobular Carcinoma	Metastasis	ER+/HER2-	MSK-IMPACT-410
MSK-ILC-86	48	Invasive Lobular Carcinoma	Metastasis	ER-/HER2+	MSK-IMPACT-341
MSK-ILC-87	51	Invasive Lobular Carcinoma	Metastasis	ER+/HER2+	MSK-IMPACT-341
MSK-ILC-88	45	Invasive Lobular Carcinoma	Primary	ER+/HER2-	MSK-IMPACT-410
MSK-ILC-89	62	Invasive Lobular Carcinoma	Primary	ER+/HER2-	MSK-IMPACT-341
MSK-ILC-90	48	Invasive Lobular Carcinoma	Primary	ER+/HER2-	MSK-IMPACT-341
MSK-ILC-91	40	Invasive Lobular Carcinoma	Metastasis	ER+/HER2-	MSK-IMPACT-410
MSK-ILC-92	61	Invasive Lobular Carcinoma	Primary	ER+/HER2-	MSK-IMPACT-341
MSK-ILC-93	55	Invasive Lobular Carcinoma	Primary	ER+/HER2-	MSK-IMPACT-341
MSK-ILC-94	55	Invasive Lobular Carcinoma	Metastasis	ER+/HER2-	MSK-IMPACT-410
MSK-ILC-95	48	Invasive Lobular Carcinoma	Primary	ER+/HER2-	MSK-IMPACT-341
MSK-ILC-96	48	Invasive Lobular Carcinoma	Metastasis	ER-/HER2-	MSK-IMPACT-410
MSK-ILC-97	46	Invasive Lobular Carcinoma	Metastasis	ER-/HER2-	MSK-IMPACT-410
MSK-ILC-98	52	Invasive Lobular Carcinoma	Metastasis	ER+/HER2-	MSK-IMPACT-341
MSK-ILC-99	54	Invasive Lobular Carcinoma	Metastasis	ER+/HER2-	MSK-IMPACT-410
MSK-ILC-100	54	Invasive Lobular Carcinoma	Metastasis	ER-/HER2-	MSK-IMPACT-410
MSK-ILC-101	58	Invasive Lobular Carcinoma	Metastasis	ER+/HER2-	MSK-IMPACT-410
MSK-ILC-102	56	Invasive Lobular Carcinoma	Metastasis	ER+/HER2-	MSK-IMPACT-410
MSK-ILC-103	44	Invasive Lobular Carcinoma	Metastasis	ER+/HER2-	MSK-IMPACT-341
MSK-ILC-104	63	Invasive Lobular Carcinoma	Metastasis	ER+/HER2-	MSK-IMPACT-341
MSK-ILC-105	73	Invasive Lobular Carcinoma	Metastasis	ER+/HER2-	MSK-IMPACT-341
MSK-ILC-106	35	Invasive Lobular Carcinoma	Metastasis	ER-/HER2-	MSK-IMPACT-410
MSK-ILC-107	47	Invasive Lobular Carcinoma	Primary	ER+/HER2-	MSK-IMPACT-410
MSK-ILC-108	39	Invasive Lobular Carcinoma	Metastasis	ER+/HER2-	MSK-IMPACT-410
MSK-ILC-109	29	Invasive Lobular Carcinoma	Primary	ER+/HER2-	MSK-IMPACT-341
MSK-ILC-110	69	Invasive Lobular Carcinoma	Metastasis	ER+/HER2+	MSK-IMPACT-410
MSK-ILC-111	45	Invasive Lobular Carcinoma	Primary	ER+/HER2-	MSK-IMPACT-468
MSK-ILC-112	56	Invasive Lobular Carcinoma	Primary	ER+/HER2-	MSK-IMPACT-410
MSK-ILC-113	50	Invasive Lobular Carcinoma	Metastasis	ER+/HER2-	MSK-IMPACT-410
MSK-ILC-114	44	Invasive Lobular Carcinoma	Metastasis	ER+/HER2-	MSK-IMPACT-341
MSK-ILC-115	49	Invasive Lobular Carcinoma	Primary	ER+/HER2-	MSK-IMPACT-341

MSK-ILC-116	53	Invasive Lobular Carcinoma	Metastasis	ER+/HER2-	MSK-IMPACT-341
MSK-ILC-117	73	Invasive Lobular Carcinoma	Metastasis	ER+/HER2+	MSK-IMPACT-410
MSK-ILC-118	73	Invasive Lobular Carcinoma	Metastasis	ER+/HER2-	MSK-IMPACT-468
MSK-ILC-119	43	Invasive Lobular Carcinoma	Metastasis	ER+/HER2-	MSK-IMPACT-410
MSK-ILC-120	50	Invasive Lobular Carcinoma	Metastasis	ER+/HER2-	MSK-IMPACT-341
MSK-ILC-121	55	Invasive Lobular Carcinoma	Primary	ER+/HER2+	MSK-IMPACT-341
MSK-ILC-122	63	Invasive Lobular Carcinoma	Primary	ER+/HER2-	MSK-IMPACT-341
MSK-ILC-123	50	Invasive Lobular Carcinoma	Metastasis	ER+/HER2-	MSK-IMPACT-341
MSK-ILC-124	50	Invasive Lobular Carcinoma	Metastasis	ER+/HER2-	MSK-IMPACT-468
MSK-ILC-125	47	Invasive Lobular Carcinoma	Metastasis	ER+/HER2+	MSK-IMPACT-341
MSK-ILC-126	38	Invasive Lobular Carcinoma	Metastasis	ER+/HER2-	MSK-IMPACT-410
MSK-ILC-127	65	Invasive Lobular Carcinoma	Metastasis	ER+/HER2-	MSK-IMPACT-341
MSK-ILC-128	54	Invasive Lobular Carcinoma	Metastasis	ER+/HER2-	MSK-IMPACT-341
MSK-ILC-129	49	Invasive Lobular Carcinoma	Primary	ER+/HER2-	MSK-IMPACT-410
MSK-ILC-130	58	Invasive Lobular Carcinoma	Primary	ER+/HER2-	MSK-IMPACT-341
MSK-ILC-131	50	Invasive Lobular Carcinoma	Metastasis	ER+/HER2-	MSK-IMPACT-341
MSK-ILC-132	50	Invasive Lobular Carcinoma	Metastasis	ER+/HER2-	MSK-IMPACT-410
MSK-ILC-133	48	Invasive Lobular Carcinoma	Metastasis	ER+/HER2-	MSK-IMPACT-341
MSK-ILC-134	41	Invasive Lobular Carcinoma	Metastasis	ER+/HER2-	MSK-IMPACT-341
MSK-ILC-135	58	Invasive Lobular Carcinoma	Primary	ER+/HER2-	MSK-IMPACT-341
MSK-ILC-136	58	Invasive Lobular Carcinoma	Metastasis	ER+/HER2-	MSK-IMPACT-410
MSK-ILC-137	46	Invasive Lobular Carcinoma	Metastasis	ER+/HER2-	MSK-IMPACT-468
MSK-ILC-138	56	Invasive Lobular Carcinoma	Metastasis	ER+/HER2-	MSK-IMPACT-341
MSK-ILC-139	53	Invasive Lobular Carcinoma	Primary	ER+/HER2-	MSK-IMPACT-341
MSK-ILC-140	53	Invasive Lobular Carcinoma	Primary	ER+/HER2-	MSK-IMPACT-341
MSK-ILC-141	45	Invasive Lobular Carcinoma	Primary	ER+/HER2-	MSK-IMPACT-341
MSK-ILC-142	67	Invasive Lobular Carcinoma	Primary	ER+/HER2-	MSK-IMPACT-410
MSK-ILC-143	50	Invasive Lobular Carcinoma	Metastasis	ER-/HER2-	MSK-IMPACT-410
MSK-ILC-144	65	Invasive Lobular Carcinoma	Metastasis	ER-/HER2-	MSK-IMPACT-410
MSK-ILC-145	62	Invasive Lobular Carcinoma	Primary	ER-/HER2-	MSK-IMPACT-410
MSK-ILC-146	41	Invasive Lobular Carcinoma	Metastasis	ER+/HER2-	MSK-IMPACT-410
MSK-ILC-147	40	Invasive Lobular Carcinoma	Metastasis	ER+/HER2-	MSK-IMPACT-410
MSK-ILC-148	71	Invasive Lobular Carcinoma	Metastasis	ER+/HER2-	MSK-IMPACT-410
MSK-ILC-149	60	Invasive Lobular Carcinoma	Metastasis	ER+/HER2-	MSK-IMPACT-410
MSK-ILC-150	39	Invasive Lobular Carcinoma	Metastasis	ER+/HER2-	MSK-IMPACT-410
MSK-ILC-151	58	Invasive Lobular Carcinoma	Primary	ER+/HER2-	MSK-IMPACT-410
MSK-ILC-152	53	Invasive Lobular Carcinoma	Primary	ER+/HER2-	MSK-IMPACT-410
MSK-ILC-153	71	Invasive Lobular Carcinoma	Primary	ER+/HER2-	MSK-IMPACT-410
MSK-ILC-154	49	Invasive Lobular Carcinoma	Metastasis	ER-/HER2-	MSK-IMPACT-410
MSK-ILC-155	50	Invasive Lobular Carcinoma	Primary	ER+/HER2-	MSK-IMPACT-410
MSK-ILC-156	56	Invasive Lobular Carcinoma	Primary	ER+/HER2-	MSK-IMPACT-410
MSK-ILC-157	57	Invasive Lobular Carcinoma	Metastasis	ER+/HER2-	MSK-IMPACT-410
MSK-ILC-158	50	Invasive Lobular Carcinoma	Primary	ER+/HER2-	MSK-IMPACT-410
MSK-ILC-159	43	Invasive Lobular Carcinoma	Metastasis	ER+/HER2-	MSK-IMPACT-410
MSK-ILC-160	63	Invasive Lobular Carcinoma	Primary	ER+/HER2-	MSK-IMPACT-410
MSK-ILC-161	45	Invasive Lobular Carcinoma	Primary	ER+/HER2-	MSK-IMPACT-468
MSK-ILC-162	49	Invasive Lobular Carcinoma	Metastasis	ER+/HER2-	MSK-IMPACT-410
MSK-ILC-163	67	Invasive Lobular Carcinoma	Metastasis	ER-/HER2-	MSK-IMPACT-410
MSK-ILC-164	48	Invasive Lobular Carcinoma	Metastasis	ER+/HER2-	MSK-IMPACT-410
MSK-ILC-165	54	Invasive Lobular Carcinoma	Metastasis	ER+/HER2-	MSK-IMPACT-410
MSK-ILC-166	55	Invasive Lobular Carcinoma	Metastasis	ER+/HER2-	MSK-IMPACT-410
MSK-ILC-167	79	Invasive Lobular Carcinoma	Primary	ER+/HER2-	MSK-IMPACT-410
MSK-ILC-168	79	Invasive Lobular Carcinoma	Primary	ER+/HER2-	MSK-IMPACT-410
MSK-ILC-169	56	Invasive Lobular Carcinoma	Metastasis	ER+/HER2-	MSK-IMPACT-410
MSK-ILC-170	58	Invasive Lobular Carcinoma	Primary	ER+/HER2-	MSK-IMPACT-410
MSK-ILC-171	54	Invasive Lobular Carcinoma	Primary	ER+/HER2-	MSK-IMPACT-468
MSK-ILC-172	47	Invasive Lobular Carcinoma	Metastasis	ER+/HER2-	MSK-IMPACT-410
MSK-ILC-173	50	Invasive Lobular Carcinoma	Primary	ER+/HER2-	MSK-IMPACT-410
MSK-ILC-174	44	Invasive Lobular Carcinoma	Metastasis	ER+/HER2-	MSK-IMPACT-410
MSK-ILC-175	39	Invasive Lobular Carcinoma	Primary	ER+/HER2-	MSK-IMPACT-468

MSK-ILC-176	64	Invasive Lobular Carcinoma	Primary	ER+/HER2-	MSK-IMPACT-468
MSK-ILC-177	54	Invasive Lobular Carcinoma	Primary	ER+/HER2-	MSK-IMPACT-468
MSK-ILC-178	47	Invasive Lobular Carcinoma	Metastasis	ER+/HER2-	MSK-IMPACT-410
MSK-ILC-179	45	Invasive Lobular Carcinoma	Primary	ER+/HER2-	MSK-IMPACT-468
MSK-ILC-180	65	Invasive Lobular Carcinoma	Primary	ER+/HER2-	MSK-IMPACT-410
MSK-ILC-181	39	Invasive Lobular Carcinoma	Primary	ER+/HER2-	MSK-IMPACT-410
MSK-ILC-182	66	Invasive Lobular Carcinoma	Primary	ER+/HER2-	MSK-IMPACT-468
MSK-ILC-183	68	Invasive Lobular Carcinoma	Metastasis	ER+/HER2-	MSK-IMPACT-410
MSK-ILC-184	57	Invasive Lobular Carcinoma	Primary	ER+/HER2-	MSK-IMPACT-410
MSK-ILC-185	51	Invasive Lobular Carcinoma	Metastasis	ER-/HER2-	MSK-IMPACT-410
MSK-ILC-186	71	Invasive Lobular Carcinoma	Primary	ER+/HER2-	MSK-IMPACT-410
MSK-ILC-187	55	Invasive Lobular Carcinoma	Metastasis	ER+/HER2-	MSK-IMPACT-410
MSK-ILC-188	53	Invasive Lobular Carcinoma	Primary	ER+/HER2-	MSK-IMPACT-410
MSK-ILC-189	77	Invasive Lobular Carcinoma	Primary	ER+/HER2-	MSK-IMPACT-410
MSK-ILC-190	73	Invasive Lobular Carcinoma	Primary	ER-/HER2-	MSK-IMPACT-410
MSK-ILC-191	51	Invasive Lobular Carcinoma	Metastasis	ER+/HER2-	MSK-IMPACT-410
MSK-ILC-192	41	Invasive Lobular Carcinoma	Metastasis	ER+/HER2-	MSK-IMPACT-410
MSK-ILC-193	38	Invasive Lobular Carcinoma	Primary	ER+/HER2-	MSK-IMPACT-468
MSK-ILC-194	74	Invasive Lobular Carcinoma	Primary	ER+/HER2-	MSK-IMPACT-468
MSK-ILC-195	57	Invasive Lobular Carcinoma	Primary	ER+/HER2-	MSK-IMPACT-410
MSK-ILC-196	52	Invasive Lobular Carcinoma	Primary	ER+/HER2-	MSK-IMPACT-410
MSK-ILC-197	78	Invasive Lobular Carcinoma	Primary	ER+/HER2-	MSK-IMPACT-468
MSK-ILC-198	57	Invasive Lobular Carcinoma	Primary	ER+/HER2-	MSK-IMPACT-468
MSK-ILC-199	64	Invasive Lobular Carcinoma	Primary	ER+/HER2-	MSK-IMPACT-468
MSK-ILC-200	60	Invasive Lobular Carcinoma	Primary	ER+/HER2-	MSK-IMPACT-468
MSK-ILC-201	72	Invasive Lobular Carcinoma	Primary	ER+/HER2-	MSK-IMPACT-468
MSK-ILC-202	51	Invasive Lobular Carcinoma	Primary	ER+/HER2-	MSK-IMPACT-468
MSK-ILC-203	78	Invasive Lobular Carcinoma	Primary	ER-/HER2-	MSK-IMPACT-468
MSK-ILC-204	45	Invasive Lobular Carcinoma	Primary	ER+/HER2-	MSK-IMPACT-410
MSK-ILC-205	49	Invasive Lobular Carcinoma	Primary	ER+/HER2-	MSK-IMPACT-410
MSK-ILC-206	59	Invasive Lobular Carcinoma	Primary	ER+/HER2-	MSK-IMPACT-410
MSK-ILC-207	55	Invasive Lobular Carcinoma	Metastasis	ER+/HER2-	MSK-IMPACT-410
MSK-ILC-208	57	Invasive Lobular Carcinoma	Primary	ER+/HER2-	MSK-IMPACT-410
MSK-ILC-209	52	Invasive Lobular Carcinoma	Primary	ER+/HER2-	MSK-IMPACT-468
MSK-ILC-210	45	Invasive Lobular Carcinoma	Primary	ER+/HER2-	MSK-IMPACT-468
MSK-ILC-211	54	Invasive Lobular Carcinoma	Metastasis	ER+/HER2-	MSK-IMPACT-468
MSK-ILC-212	46	Invasive Lobular Carcinoma	Metastasis	ER+/HER2-	MSK-IMPACT-410
MSK-ILC-213	72	Invasive Lobular Carcinoma	Primary	ER+/HER2-	MSK-IMPACT-468
MSK-ILC-214	41	Invasive Lobular Carcinoma	Primary	ER+/HER2-	MSK-IMPACT-410
MSK-ILC-215	48	Invasive Lobular Carcinoma	Primary	ER+/HER2-	MSK-IMPACT-410
MSK-ILC-216	35	Invasive Lobular Carcinoma	Primary	ER+/HER2-	MSK-IMPACT-410
MSK-ILC-217	58	Invasive Lobular Carcinoma	Metastasis	ER+/HER2-	MSK-IMPACT-410
MSK-ILC-218	47	Invasive Lobular Carcinoma	Primary	ER+/HER2-	MSK-IMPACT-410
MSK-ILC-219	49	Invasive Lobular Carcinoma	Primary	ER+/HER2-	MSK-IMPACT-468
MSK-ILC-220	76	Invasive Lobular Carcinoma	Primary	ER+/HER2-	MSK-IMPACT-410
MSK-ILC-221	70	Invasive Lobular Carcinoma	Primary	ER+/HER2-	MSK-IMPACT-468
MSK-ILC-222	41	Invasive Lobular Carcinoma	Primary	ER+/HER2-	MSK-IMPACT-468
MSK-ILC-223	62	Invasive Lobular Carcinoma	Primary	ER+/HER2-	MSK-IMPACT-468
MSK-ILC-224	48	Invasive Lobular Carcinoma	Primary	ER+/HER2-	MSK-IMPACT-468
MSK-ILC-225	55	Invasive Lobular Carcinoma	Primary	ER+/HER2-	MSK-IMPACT-468
MSK-ILC-226	36	Invasive Lobular Carcinoma	Metastasis	ER+/HER2-	MSK-IMPACT-468
MSK-ILC-227	62	Invasive Lobular Carcinoma	Primary	ER+/HER2-	MSK-IMPACT-468
MSK-ILC-228	63	Invasive Lobular Carcinoma	Metastasis	ER+/HER2-	MSK-IMPACT-468
MSK-ILC-229	71	Invasive Lobular Carcinoma	Metastasis	ER+/HER2-	MSK-IMPACT-468
MSK-ILC-230	49	Invasive Lobular Carcinoma	Metastasis	ER-/HER2-	MSK-IMPACT-468
MSK-ILC-231	65	Invasive Lobular Carcinoma	Primary	ER+/HER2-	MSK-IMPACT-468
MSK-ILC-232	59	Invasive Lobular Carcinoma	Primary	ER+/HER2-	MSK-IMPACT-468
MSK-ILC-233	53	Invasive Lobular Carcinoma	Primary	ER+/HER2-	MSK-IMPACT-468
MSK-ILC-234	53	Invasive Lobular Carcinoma	Primary	ER+/HER2-	MSK-IMPACT-468
MSK-ILC-235	47	Invasive Lobular Carcinoma	Primary	ER+/HER2-	MSK-IMPACT-468

MSK-ILC-236	49	Invasive Lobular Carcinoma	Primary	ER+/HER2-	MSK-IMPACT-468
MSK-ILC-237	71	Invasive Lobular Carcinoma	Primary	ER+/HER2-	MSK-IMPACT-468
MSK-ILC-238	72	Invasive Lobular Carcinoma	Metastasis	ER+/HER2-	MSK-IMPACT-468
MSK-ILC-239	54	Invasive Lobular Carcinoma	Primary	ER+/HER2-	MSK-IMPACT-468
MSK-ILC-240	38	Invasive Lobular Carcinoma	Primary	ER+/HER2-	MSK-IMPACT-468
MSK-ILC-241	48	Invasive Lobular Carcinoma	Primary	ER+/HER2-	MSK-IMPACT-468
MSK-ILC-242	69	Invasive Lobular Carcinoma	Primary	ER+/HER2-	MSK-IMPACT-468
MSK-ILC-243	64	Invasive Lobular Carcinoma	Primary	ER+/HER2-	MSK-IMPACT-468
MSK-ILC-244	49	Invasive Lobular Carcinoma	Metastasis	ER+/HER2-	MSK-IMPACT-468
MSK-ILC-245	74	Invasive Lobular Carcinoma	Primary	ER+/HER2-	MSK-IMPACT-468
MSK-ILC-246	67	Invasive Lobular Carcinoma	Primary	ER+/HER2-	MSK-IMPACT-468
MSK-ILC-247	58	Invasive Lobular Carcinoma	Primary	ER+/HER2-	MSK-IMPACT-468
MSK-ILC-248	63	Invasive Lobular Carcinoma	Primary	ER+/HER2-	MSK-IMPACT-468
MSK-ILC-249	49	Invasive Lobular Carcinoma	Primary	ER+/HER2-	MSK-IMPACT-468
MSK-ILC-250	49	Invasive Lobular Carcinoma	Primary	ER+/HER2-	MSK-IMPACT-468
MSK-ILC-251	57	Invasive Lobular Carcinoma	Primary	ER+/HER2-	MSK-IMPACT-468
MSK-ILC-252	51	Invasive Lobular Carcinoma	Primary	ER+/HER2-	MSK-IMPACT-468
MSK-ILC-253	53	Invasive Lobular Carcinoma	Primary	ER+/HER2-	MSK-IMPACT-468
MSK-ILC-254	72	Invasive Lobular Carcinoma	Primary	ER+/HER2-	MSK-IMPACT-468
MSK-ILC-255	66	Invasive Lobular Carcinoma	Primary	ER+/HER2-	MSK-IMPACT-468
MSK-ILC-256	64	Invasive Lobular Carcinoma	Primary	ER+/HER2-	MSK-IMPACT-468
MSK-ILC-257	75	Invasive Lobular Carcinoma	Primary	ER+/HER2-	MSK-IMPACT-468
MSK-ILC-258	73	Invasive Lobular Carcinoma	Primary	ER+/HER2-	MSK-IMPACT-468
MSK-ILC-259	56	Invasive Lobular Carcinoma	Primary	ER+/HER2-	MSK-IMPACT-468
MSK-META-1	54	Metaplastic Carcinoma	Primary	ER-/HER2-	MSK-IMPACT-410
MSK-META-2	34	Metaplastic Carcinoma	Metastasis	ER-/HER2-	MSK-IMPACT-341
MSK-META-3	53	Metaplastic Carcinoma	Metastasis	ER-/HER2-	MSK-IMPACT-410
MSK-META-4	41	Metaplastic Carcinoma	Metastasis	ER+/HER2-	MSK-IMPACT-410
MSK-META-5	43	Metaplastic Carcinoma	Primary	ER-/HER2-	MSK-IMPACT-341
MSK-META-6	64	Metaplastic Carcinoma	Primary	ER+/HER2-	MSK-IMPACT-341
MSK-META-7	59	Metaplastic Carcinoma	Metastasis	ER-/HER2-	MSK-IMPACT-341
MSK-META-8	45	Metaplastic Carcinoma	Metastasis	ER-/HER2-	MSK-IMPACT-410
MSK-META-9	39	Metaplastic Carcinoma	Primary	ER-/HER2-	MSK-IMPACT-341
MSK-META-10	47	Metaplastic Carcinoma	Metastasis	ER-/HER2-	MSK-IMPACT-341
MSK-META-11	53	Metaplastic Carcinoma	Primary	ER-/HER2-	MSK-IMPACT-468
MSK-MPC-1	81	Micropapillary Carcinoma	Primary	ER+/HER2-	MSK-IMPACT-468
MSK-MPC-2	38	Micropapillary Carcinoma	Primary	ER+/HER2+	MSK-IMPACT-410
MSK-MPC-3	59	Micropapillary Carcinoma	Metastasis	ER+/HER2-	MSK-IMPACT-410
MSK-MPC-4	51	Micropapillary Carcinoma	Metastasis	ER-/HER2-	MSK-IMPACT-410
MSK-MPC-5	58	Micropapillary Carcinoma	Metastasis	ER+/HER2-	MSK-IMPACT-410
MSK-MPC-6	46	Micropapillary Carcinoma	Primary	ER+/HER2-	MSK-IMPACT-410
MSK-MPC-7	44	Micropapillary Carcinoma	Primary	ER-/HER2+	MSK-IMPACT-468
MSK-MPC-8	38	Micropapillary Carcinoma	Metastasis	ER-/HER2-	MSK-IMPACT-410
MSK-MPC-9	41	Micropapillary Carcinoma	Metastasis	ER+/HER2+	MSK-IMPACT-341
MSK-MPC-10	67	Micropapillary Carcinoma	Primary	ER+/HER2-	MSK-IMPACT-410
MSK-MPC-11	41	Micropapillary Carcinoma	Primary	ER+/HER2-	MSK-IMPACT-410
MSK-MPC-12	63	Micropapillary Carcinoma	Metastasis	ER+/HER2-	MSK-IMPACT-410
MSK-MPC-13	63	Micropapillary Carcinoma	Metastasis	ER+/HER2-	MSK-IMPACT-410
MSK-MPC-14	32	Micropapillary Carcinoma	Metastasis	ER+/HER2-	MSK-IMPACT-410
MSK-MPC-15	42	Micropapillary Carcinoma	Metastasis	ER+/HER2-	MSK-IMPACT-410
MSK-MPC-16	64	Micropapillary Carcinoma	Metastasis	ER+/HER2-	MSK-IMPACT-410
MSK-MPC-17	59	Micropapillary Carcinoma	Primary	ER+/HER2+	MSK-IMPACT-410
MSK-MPC-18	62	Micropapillary Carcinoma	Metastasis	ER+/HER2-	MSK-IMPACT-410
MSK-MPC-19	53	Micropapillary Carcinoma	Primary	ER+/HER2-	MSK-IMPACT-410
MSK-MPC-20	66	Micropapillary Carcinoma	Metastasis	ER+/HER2-	MSK-IMPACT-468
MSK-MUC-1	57	Mixed Mucinous Carcinoma	Primary	ER+/HER2-	MSK-IMPACT-410
MSK-MUC-2	71	Mixed Mucinous Carcinoma	Metastasis	ER+/HER2-	MSK-IMPACT-410
MSK-MUC-3	56	Mixed Mucinous Carcinoma	Primary	ER+/HER2-	MSK-IMPACT-468
MSK-MUC-4	36	Mixed Mucinous Carcinoma	Metastasis	ER+/HER2-	MSK-IMPACT-410
MSK-MUC-5	62	Mixed Mucinous Carcinoma	Metastasis	ER+/HER2-	MSK-IMPACT-341

MSK-MUC-6	54	Mixed Mucinous Carcinoma	Primary	ER+/HER2-	MSK-IMPACT-468
MSK-MUC-7	32	Mixed Mucinous Carcinoma	Metastasis	ER+/HER2-	MSK-IMPACT-410
MSK-MUC-8	47	Mixed Mucinous Carcinoma	Primary	ER+/HER2-	MSK-IMPACT-341
MSK-MUC-9	29	Mixed Mucinous Carcinoma	Primary	ER+/HER2+	MSK-IMPACT-341
MSK-MUC-10	50	Mixed Mucinous Carcinoma	Primary	ER+/HER2+	MSK-IMPACT-410
MSK-MUC-11	63	Mixed Mucinous Carcinoma	Primary	ER+/HER2-	MSK-IMPACT-410
MSK-MUC-12	36	Mixed Mucinous Carcinoma	Primary	ER+/HER2-	MSK-IMPACT-410
MSK-MUC-13	40	Mixed Mucinous Carcinoma	Primary	ER+/HER2-	MSK-IMPACT-468
MSK-MUC-14	46	Mixed Mucinous Carcinoma	Primary	ER+/HER2+	MSK-IMPACT-410
MSK-MUC-15	58	Mixed Mucinous Carcinoma	Metastasis	ER+/HER2-	MSK-IMPACT-410
MSK-MUC-16	32	Mixed Mucinous Carcinoma	Primary	ER+/HER2-	MSK-IMPACT-410
MSK-MUC-17	46	Mixed Mucinous Carcinoma	Primary	ER+/HER2-	MSK-IMPACT-468
MSK-MUC-18	62	Mixed Mucinous Carcinoma	Primary	ER+/HER2-	MSK-IMPACT-468
MSK-MUC-19	46	Mixed Mucinous Carcinoma	Primary	ER+/HER2-	MSK-IMPACT-468

Supplementary Table 3. Comparison of the frequency of altered genes in breast cancers of histologic special type and invasive ductal carcinomas of no special type matched by clinicopathologic characteristics subjected to targeted massively parallel sequencing (MSK-IMPACT)

Gene Name	Invasive lobular carcinoma							Mixed mucinous breast carcinoma							Micropapillary breast cancer							Metaplastic breast cancer						
	Number of cases altered (%)			Metastasis ILC vs metastasis IDC-NST		Primary ILC vs Metastasis ILC		Number of cases altered (%)			Metastasis mixed mucinous vs metastasis IDC-NST		Primary mixed mucinous vs metastasis mixed mucinous		Number of cases altered (%)			Metastasis micropapillary vs metastasis IDC-NST		Primary micropapillary vs metastasis micropapillary		Number of cases altered (%)			Metastasis metaplastic vs metastasis IDC-NST		Primary metaplastic vs metastasis metaplastic	
	Metastasis ILC (*n*=132)	Metastasis IDC-NST (*n*=264)	Primary ILC (*n*=127)	*P* value	*q* value*	*P* value	*q* value*	Metastasis mixed mucinous (*n*=5)	Metastasis IDC-NST (*n*=15)	Primary mixed mucinous (*n*=14)	*P* value	*q* value*	*P* value	*q* value*	Metastasis Micropapillary (*n*=12)	Metastasis IDC-NST (*n*=36)	Primary Micropapillary (*n*=8)	*P* value	*q* value*	*P* value	*q* value*	Metastasis Metaplastic (*n*=6)	Metastasis IDC-NST (*n*=18)	Primary Metaplastic (*n*=5)	*P* value	*q* value*	*P* value	*q* value*
ABL1	3 (2.27%)	0 (0%)	0 (0%)	0.0365	0.645172	0.2474	1	1 (20%)	0 (0%)	0 (0%)	0.25	1	0.2632	1	0 (0%)	0 (0%)	0 (0%)	1	1	1	1	0 (0%)	0 (0%)	0 (0%)	1	1	1	1
AKT1	10 (7.58%)	23 (8.71%)	9 (7.09%)	0.8475	1	1	1	0 (0%)	1 (6.67%)	1 (7.14%)	1	1	1	1	0 (0%)	7 (19.44%)	0 (0%)	0.1686	1	1	1	0 (0%)	3 (16.67%)	0 (0%)	0.5464	1	1	1
AKT2	1 (0.76%)	3 (1.14%)	0 (0%)	1	1	1	1	0 (0%)	0 (0%)	0 (0%)	1	1	1	1	0 (0%)	1 (2.78%)	0 (0%)	1	1	1	1	0 (0%)	1 (5.56%)	0 (0%)	1	1	1	1
AKT3	3 (2.27%)	6 (2.27%)	1 (0.79%)	1	1	0.6224	1	0 (0%)	0 (0%)	0 (0%)	1	1	1	1	0 (0%)	0 (0%)	0 (0%)	1	1	1	1	0 (0%)	0 (0%)	0 (0%)	1	1	1	1
ALK	5 (3.79%)	3 (1.14%)	0 (0%)	0.1234	0.895125	0.0603	1	0 (0%)	0 (0%)	1 (7.14%)	1	1	1	1	0 (0%)	0 (0%)	1 (12.5%)	1	1	0.4	1	0 (0%)	0 (0%)	0 (0%)	1	1	1	1
ALOX12B	2 (1.52%)	2 (0.76%)	0 (0%)	0.6034	1	0.4982	1	0 (0%)	0 (0%)	0 (0%)	1	1	1	1	0 (0%)	0 (0%)	0 (0%)	1	1	1	1	0 (0%)	0 (0%)	0 (0%)	1	1	1	1
AMER1	1 (0.76%)	2 (0.76%)	0 (0%)	1	1	1	1	0 (0%)	0 (0%)	0 (0%)	1	1	1	1	1 (8.33%)	0 (0%)	0 (0%)	0.25	1	1	1	0 (0%)	0 (0%)	0 (0%)	1	1	1	1
APC	7 (5.3%)	6 (2.27%)	0 (0%)	0.1361	0.9471449	0.0145	0.66836	0 (0%)	0 (0%)	0 (0%)	1	1	1	1	0 (0%)	0 (0%)	0 (0%)	1	1	1	1	1 (16.67%)	1 (5.56%)	0 (0%)	0.4457	1	1	1
AR	5 (3.79%)	7 (2.65%)	1 (0.79%)	0.5443	1	0.2138	1	0 (0%)	0 (0%)	0 (0%)	1	1	1	1	0 (0%)	0 (0%)	0 (0%)	1	1	1	1	0 (0%)	0 (0%)	0 (0%)	1	1	1	1
ARAF	0 (0%)	5 (1.89%)	1 (0.79%)	0.1742	0.9625	0.4903	1	0 (0%)	0 (0%)	0 (0%)	1	1	1	1	0 (0%)	0 (0%)	0 (0%)	1	1	1	1	0 (0%)	0 (0%)	0 (0%)	1	1	1	1
ARID1A	15 (11.36%)	19 (7.2%)	10 (7.87%)	0.1841	0.9789323	0.4029	1	1 (20%)	1 (6.67%)	0 (0%)	0.4474	1	0.2632	1	1 (8.33%)	1 (2.78%)	0 (0%)	0.4415	1	1	1	0 (0%)	2 (11.11%)	0 (0%)	1	1	1	1
ARID1B	3 (2.27%)	6 (2.27%)	1 (0.79%)	1	1	0.6224	1	0 (0%)	0 (0%)	0 (0%)	1	1	1	1	2 (16.67%)	0 (0%)	0 (0%)	0.0585	1	0.4947	1	0 (0%)	0 (0%)	0 (0%)	1	1	1	1
ARID2	4 (3.03%)	8 (3.03%)	0 (0%)	1	1	0.1224	1	0 (0%)	0 (0%)	0 (0%)	1	1	1	1	1 (8.33%)	1 (2.78%)	1 (12.5%)	0.4415	1	1	1	0 (0%)	2 (11.11%)	0 (0%)	1	1	1	1
ARID5B	4 (3.03%)	4 (1.52%)	1 (0.79%)	0.4492	1	0.3706	1	0 (0%)	0 (0%)	0 (0%)	1	1	1	1	0 (0%)	0 (0%)	0 (0%)	1	1	1	1	1 (16.67%)	0 (0%)	0 (0%)	0.25	1	1	1
ASXL1	2 (1.52%)	2 (0.76%)	1 (0.79%)	0.6034	1	1	1	0 (0%)	0 (0%)	0 (0%)	1	1	1	1	0 (0%)	1 (2.78%)	0 (0%)	1	1	1	1	1 (16.67%)	1 (5.56%)	0 (0%)	0.4457	1	1	1
ASXL2	3 (2.27%)	3 (1.14%)	0 (0%)	0.4049	1	0.2474	1	0 (0%)	0 (0%)	0 (0%)	1	1	1	1	0 (0%)	0 (0%)	0 (0%)	1	1	1	1	0 (0%)	0 (0%)	0 (0%)	1	1	1	1
ATM	7 (5.3%)	8 (3.03%)	0 (0%)	0.2743	1	0.0145	0.66836	1 (20%)	0 (0%)	0 (0%)	0.25	1	0.2632	1	0 (0%)	0 (0%)	0 (0%)	1	1	1	1	0 (0%)	0 (0%)	0 (0%)	1	1	1	1
ATR	4 (3.03%)	7 (2.65%)	2 (1.57%)	1	1	0.6842	1	0 (0%)	0 (0%)	0 (0%)	1	1	1	1	2 (16.67%)	0 (0%)	0 (0%)	0.0585	1	0.4947	1	0 (0%)	1 (5.56%)	0 (0%)	1	1	1	1
ATRX	4 (3.03%)	6 (2.27%)	2 (1.57%)	0.7369	1	0.6842	1	0 (0%)	0 (0%)	0 (0%)	1	1	1	1	0 (0%)	0 (0%)	0 (0%)	1	1	1	1	0 (0%)	3 (16.67%)	0 (0%)	0.5464	1	1	1
AURKA	1 (0.76%)	18 (6.82%)	1 (0.79%)	0.0055	0.2344375	1	1	0 (0%)	0 (0%)	0 (0%)	1	1	1	1	0 (0%)	0 (0%)	0 (0%)	1	1	1	1	0 (0%)	0 (0%)	0 (0%)	1	1	1	1
AURKB	1 (0.76%)	0 (0%)	0 (0%)	0.3333	1	1	1	0 (0%)	0 (0%)	0 (0%)	1	1	1	1	0 (0%)	0 (0%)	0 (0%)	1	1	1	1	0 (0%)	0 (0%)	0 (0%)	1	1	1	1
AXIN1	0 (0%)	2 (0.76%)	1 (0.79%)	0.5544	1	0.4903	1	0 (0%)	0 (0%)	0 (0%)	1	1	1	1	0 (0%)	1 (2.78%)	0 (0%)	1	1	1	1	0 (0%)	0 (0%)	0 (0%)	1	1	1	1
AXIN2	2 (1.52%)	9 (3.41%)	0 (0%)	0.3493	1	0.4982	1	0 (0%)	0 (0%)	1 (7.14%)	1	1	1	1	0 (0%)	0 (0%)	0 (0%)	1	1	1	1	0 (0%)	0 (0%)	0 (0%)	1	1	1	1
AXL	1 (0.76%)	3 (1.14%)	0 (0%)	1	1	1	1	0 (0%)	0 (0%)	0 (0%)	1	1	1	1	0 (0%)	0 (0%)	0 (0%)	1	1	1	1	0 (0%)	0 (0%)	0 (0%)	1	1	1	1
B2M	0 (0%)	1 (0.38%)	0 (0%)	1	1	1	1	0 (0%)	0 (0%)	0 (0%)	1	1	1	1	0 (0%)	0 (0%)	0 (0%)	1	1	1	1	0 (0%)	0 (0%)	0 (0%)	1	1	1	1
BAP1	2 (1.52%)	2 (0.76%)	0 (0%)	0.6034	1	0.4982	1	0 (0%)	0 (0%)	0 (0%)	1	1	1	1	0 (0%)	1 (2.78%)	0 (0%)	1	1	1	1	0 (0%)	1 (5.56%)	0 (0%)	1	1	1	1
BARD1	2 (1.52%)	2 (0.76%)	0 (0%)	0.6034	1	0.4982	1	0 (0%)	0 (0%)	0 (0%)	1	1	1	1	1 (8.33%)	0 (0%)	0 (0%)	0.25	1	1	1	0 (0%)	0 (0%)	0 (0%)	1	1	1	1
BBC3	1 (0.76%)	1 (0.38%)	0 (0%)	1	1	1	1	0 (0%)	0 (0%)	0 (0%)	1	1	1	1	0 (0%)	0 (0%)	0 (0%)	1	1	1	1	0 (0%)	0 (0%)	0 (0%)	1	1	1	1
BCL2	0 (0%)	0 (0%)	0 (0%)	1	1	1	1	0 (0%)	0 (0%)	0 (0%)	1	1	1	1	0 (0%)	0 (0%)	0 (0%)	1	1	1	1	0 (0%)	0 (0%)	0 (0%)	1	1	1	1
BCL2L1	0 (0%)	1 (0.38%)	0 (0%)	1	1	1	1	0 (0%)	0 (0%)	0 (0%)	1	1	1	1	0 (0%)	0 (0%)	0 (0%)	1	1	1	1	0 (0%)	0 (0%)	0 (0%)	1	1	1	1
BCL2L11	1 (0.76%)	0 (0%)	0 (0%)	0.3333	1	1	1	0 (0%)	0 (0%)	0 (0%)	1	1	1	1	0 (0%)	0 (0%)	0 (0%)	1	1	1	1	1 (16.67%)	0 (0%)	0 (0%)	0.25	1	1	1
BCL6	3 (2.27%)	3 (1.14%)	2 (1.57%)	0.4049	1	1	1	0 (0%)	0 (0%)	0 (0%)	1	1	1	1	0 (0%)	0 (0%)	0 (0%)	1	1	1	1	0 (0%)	0 (0%)	0 (0%)	1	1	1	1
BCOR	3 (2.27%)	4 (1.52%)	2 (1.57%)	0.6904	1	1	1	0 (0%)	0 (0%)	0 (0%)	1	1	1	1	0 (0%)	0 (0%)	0 (0%)	1	1	1	1	1 (16.67%)	1 (5.56%)	0 (0%)	0.4457	1	1	1
BLM	5 (3.79%)	1 (0.38%)	0 (0%)	0.0171	0.4485462	0.0603	1	0 (0%)	0 (0%)	0 (0%)	1	1	1	1	0 (0%)	0 (0%)	0 (0%)	1	1	1	1	0 (0%)	0 (0%)	0 (0%)	1	1	1	1
BMPR1A	0 (0%)	4 (1.52%)	0 (0%)	0.306	1	1	1	0 (0%)	0 (0%)	0 (0%)	1	1	1	1	0 (0%)	0 (0%)	0 (0%)	1	1	1	1	1 (16.67%)	0 (0%)	0 (0%)	0.25	1	1	1
BRAF	0 (0%)	4 (1.52%)	2 (1.57%)	0.306	1	0.2395	1	0 (0%)	1 (6.67%)	0 (0%)	1	1	1	1	0 (0%)	2 (5.56%)	0 (0%)	1	1	1	1	0 (0%)	1 (5.56%)	0 (0%)	1	1	1	1
BRCA1	2 (1.52%)	9 (3.41%)	0 (0%)	0.3493	1	0.4982	1	0 (0%)	0 (0%)	1 (7.14%)	1	1	1	1	0 (0%)	1 (2.78%)	0 (0%)	1	1	1	1	0 (0%)	1 (5.56%)	0 (0%)	1	1	1	1
BRCA2	9 (6.82%)	8 (3.03%)	5 (3.94%)	0.1119	0.867225	0.4121	1	0 (0%)	0 (0%)	1 (7.14%)	1	1	1	1	1 (8.33%)	1 (2.78%)	0 (0%)	0.4415	1	1	1	0 (0%)	0 (0%)	1 (20%)	1	1	0.4545	1
BRD4	2 (1.52%)	3 (1.14%)	0 (0%)	1	1	0.4982	1	0 (0%)	0 (0%)	0 (0%)	1	1	1	1	0 (0%)	0 (0%)	0 (0%)	1	1	1	1	0 (0%)	0 (0%)	0 (0%)	1	1	1	1
BRIP1	1 (0.76%)	17 (6.44%)	0 (0%)	0.0091	0.31031	1	1	0 (0%)	0 (0%)	3 (21.43%)	1	1	0.5304	1	1 (8.33%)	0 (0%)	1 (12.5%)	0.25	1	1	1	1 (16.67%)	0 (0%)	0 (0%)	0.25	1	1	1
BTK	3 (2.27%)	4 (1.52%)	0 (0%)	0.6904	1	0.2474	1	0 (0%)	1 (6.67%)	0 (0%)	1	1	1	1	0 (0%)	1 (2.78%)	0 (0%)	1	1	1	1	0 (0%)	0 (0%)	0 (0%)	1	1	1	1
CARD11	1 (0.76%)	6 (2.27%)	0 (0%)	0.4324	1	1	1	0 (0%)	0 (0%)	0 (0%)	1	1	1	1	0 (0%)	1 (2.78%)	0 (0%)	1	1	1	1	0 (0%)	2 (11.11%)	0 (0%)	1	1	1	1
CASP8	0 (0%)	2 (0.76%)	0 (0%)	0.5544	1	1	1	0 (0%)	0 (0%)	0 (0%)	1	1	1	1	0 (0%)	0 (0%)	0 (0%)	1	1	1	1	0 (0%)	0 (0%)	0 (0%)	1	1	1	1
CBFB	5 (3.79%)	17 (6.44%)	10 (7.87%)	0.3554	1	0.1896	1	1 (20%)	3 (20%)	0 (0%)	1	1	0.2632	1	0 (0%)	4 (11.11%)	0 (0%)	0.5597	1	1	1	0 (0%)	2 (11.11%)	0 (0%)	1	1	1	1
CBL	1 (0.76%)	0 (0%)	1 (0.79%)	0.3333	1	1	1	0 (0%)	0 (0%)	0 (0%)	1	1	1	1	0 (0%)	0 (0%)	0 (0%)	1	1	1	1	0 (0%)	0 (0%)	0 (0%)	1	1	1	1
CCND1	23 (17.42%)	67 (25.38%)	21 (16.54%)	0.0768	0.867225	0.87	1	3 (60%)	1 (6.67%)	5 (35.71%)	0.032	1	0.6027	1	2 (16.67%)	3 (8.33%)	3 (37.5%)	0.5872	1	0.3473	1	2 (33.33%)	1 (5.56%)	0 (0%)	0.1433	1	0.4545	1
CCND2	1 (0.76%)	4 (1.52%)	0 (0%)	0.6687	1	1	1	0 (0%)	0 (0%)	0 (0%)	1	1	1	1	0 (0%)	0 (0%)	0 (0%)	1	1	1	1	0 (0%)	0 (0%)	0 (0%)	1	1	1	1
CCND3	0 (0%)	2 (0.76%)	0 (0%)	0.5544	1	1	1	0 (0%)	0 (0%)	0 (0%)	1	1	1	1	0 (0%)	0 (0%)	0 (0%)	1	1	1	1	0 (0%)	0 (0%)	0 (0%)	1	1	1	1
CCNE1	0 (0%)	7 (2.65%)	0 (0%)	0.1008	0.867225	1	1	0 (0%)	1 (6.67%)	0 (0%)	1	1	1	1	0 (0%)	4 (11.11%)	0 (0%)	0.5597	1	1	1	0 (0%)	3 (16.67%)	1 (20%)	0.5464	1	0.4545	1
CD274	1 (0.76%)	4 (1.52%)	0 (0%)	0.6687	1	1	1	0 (0%)	0 (0%)	0 (0%)	1	1	1	1	0 (0%)	1 (2.78%)	0 (0%)	1	1	1	1	0 (0%)	0 (0%)	0 (0%)	1	1	1	1
CD276	0 (0%)	1 (0.38%)	1 (0.79%)	1	1	0.4903	1	0 (0%)	0 (0%)	0 (0%)	1	1	1	1	0 (0%)	0 (0%)	0 (0%)	1	1	1	1	0 (0%)	0 (0%)	0 (0%)	1	1	1	1
CD79B	0 (0%)	10 (3.79%)	1 (0.79%)	0.0347	0.645172	0.4903	1	0 (0%)	2 (13.33%)	1 (7.14%)	1	1	1	1	0 (0%)	2 (5.56%)	0 (0%)	1	1	1	1	0 (0%)	0 (0%)	0 (0%)	1	1	1	1
CDC73	3 (2.27%)	8 (3.03%)	1 (0.79%)	0.7582	1	0.6224	1	0 (0%)	0 (0%)	0 (0%)	1	1	1	1	0 (0%)	1 (2.78%)	0 (0%)	1	1	1	1	0 (0%)	1 (5.56%)	0 (0%)	1	1	1	1
CDH1	100 (75.76%)	9 (3.41%)	104 (81.89%)	9.8×10^{-54}	3.3×10^{-51}	0.2874	1	0 (0%)	0 (0%)	0 (0%)	1	1	1	1	0 (0%)	1 (2.78%)	0 (0%)	1	1	1	1	0 (0%)	2 (11.11%)	0 (0%)	1	1	1	1
CDK12	6 (4.55%)	17 (6.44%)	2 (1.57%)	0.5037	1	0.2821	1	0 (0%)	1 (6.67%)	2 (14.29%)	1	1	1	1	1 (8.33%)	1 (2.78%)	2 (25%)	0.4415	1	0.5368	1	0 (0%)	0 (0%)	0 (0%)	1	1	1	1
CDK4	0 (0%)	5 (1.89%)	0 (0%)	0.1742	0.9625	1	1	0 (0%)	0 (0%)	0 (0%)	1	1	1	1	0 (0%)	0 (0%)	0 (0%)	1	1	1	1	1 (16.67%)	0 (0%)	0 (0%)	0.25	1	1	1
CDK6	1 (0.76%)	3 (1.14%)	0 (0%)	1	1	1	1	0 (0%)	0 (0%)	0 (0%)	1	1	1	1	0 (0%)	0 (0%)	0 (0%)	1	1	1	1	0 (0%)	0 (0%)	0 (0%)	1	1	1	1
CDK8	1 (0.76%)	5 (1.89%)	0 (0%)	0.6683	1	1	1	0 (0%)	0 (0%)	0 (0%)	1	1	1	1	0 (0%)	1 (2.78%)	0 (0%)	1	1	1	1	0 (0%)	1 (5.56%)	0 (0%)	1	1	1	1
CDKN1A	1 (0.76%)	5 (1.89%)	0 (0%)	0.6683	1	1	1	0 (0%)	0 (0%)	0 (0%)	1	1	1	1	0 (0%)	0 (0%)	0 (0%)	1	1	1	1	0 (0%)	1 (5.56%)	0 (0%)	1	1	1	1
CDKN1B	4 (3.03%)	10 (3.79%)	3 (2.36%)	0.7818	1	1	1	0 (0%)	1 (6.67%)	0 (0%)	1	1	1	1	0 (0%)	1 (2.78%)	0 (0%)	1	1	1	1	0 (0%)	0 (0%)	1 (20%)	1	1	0.4545	1
CDKN2A	3 (2.27%)	5 (1.89%)	1 (0.79%)	1	1	0.6224	1	0 (0%)	0 (0%)	1 (7.14%)	1	1	1	1	0 (0%)	0 (0%)	0 (0%)	1	1	1	1	1 (16.67%)	1 (5.56%)	1 (20%)	0.4457	1	1	1
CDKN2B	3 (2.27%)	5 (1.89%)	0 (0%)	1	1	0.2474	1	0 (0%)	0 (0%)	1 (7.14%)	1	1	1	1	0 (0%)	0 (0%)	0 (0%)	1	1	1	1	1 (16.67%)	1 (5.56%)	0 (0%)	0.4457	1	1	1
CDKN2C	0 (0%)	1 (0.38%)	0 (0%)	1	1	1	1	0 (0%)	0 (0%)	0 (0%)	1	1	1	1	0 (0%)	0 (0%)	0 (0%)	1	1	1	1	0 (0%)	0 (0%)	0 (0%)	1	1	1	1
CHEK1	0 (0%)	1 (0.38%)	0 (0%)	1	1	1	1	0 (0%)	0 (0%)	0 (0%)	1	1	1	1	0 (0%)	0 (0%)	0 (0%)	1	1	1	1	0 (0%)	0 (0%)	0 (0%)	1	1	1	1
CHEK2	3 (2.27%)	1 (0.38%)	0 (0%)	0.11	0.867225	0.2474	1	0 (0%)	0 (0%)	0 (0%)	1	1	1	1	0 (0%)	1 (2.78%)	0 (0%)	1	1	1	1	0 (0%)	0 (0%)	0 (0%)	1	1	1	1
CIC	2 (1.52%)	7 (2.65%)	0 (0%)	0.7237	1	0.4982	1	0 (0%)	1 (6.67%)	0 (0%)	1	1	1	1	0 (0%)	1 (2.78%)	0 (0%)	1	1	1	1	0 (0%)	1 (5.56%)	0 (0%)	1	1	1	1
CREBBP	9 (6.82%)	6 (2.27%)	1 (0.79%)	0.0461	0.645172	0.0194	0.66836	0 (0%)	0 (0%)	0 (0%)	1	1	1	1	0 (0%)	0 (0%)	0 (0%)	1	1	1	1	0 (0%)	1 (5.56%)	0 (0%)	1	1	1	1
CRKL	0 (0%)	0 (0%)	0 (0%)	1	1	1	1	0 (0%)	0 (0%)	0 (0%)	1	1	1	1	0 (0%)	0 (0%)	0 (0%)	1	1	1	1	0 (0%)	0 (0%)	0 (0%)	1	1	1	1
CRLF2	0 (0%)	3 (1.14%)	0 (0%)	0.5539	1	1	1	0 (0%)	0 (0%)	1 (7.14%)	1	1	1	1	0 (0%)	0 (0%)	0 (0%)	1	1	1	1	0 (0%)	0 (0%)	0 (0%)	1	1	1	1
CSF1R	2 (1.52%)	0 (0%)	0 (0%)	0.1105	0.867225	0.4982	1	0 (0%)	0 (0%)	0 (0%)	1	1	1	1	0 (0%)	0 (0%)	0 (0%)	1	1	1	1	0 (0%)	0 (0%)	0 (0%)	1	1	1	1
CTCF	2 (1.52%)	7 (2.65%)	3 (2.36%)	0.7237	1	0.6791	1	0 (0%)	0 (0%)	0 (0%)	1	1	1	1	0 (0%)	0 (0%)	0 (0%)	1	1	1	1	0 (0%)	0 (0%)	0 (0%)	1	1	1	1
CTLA4	0 (0%)	1 (0.38%)	0 (0%)	1	1	1	1	0 (0%)	0 (0%)	0 (0%)	1	1	1	1	0 (0%)	0 (0%)	0 (0%)	1	1	1	1	0 (0%)	0 (0%)	0 (0%)	1	1	1	1
CTNNB1	1 (0.76%)	3 (1.14%)	0 (0%)	1	1	1	1	0 (0%)	0 (0%)	0 (0%)	1	1	1	1	0 (0%)	1 (2.78%)	0 (0%)	1	1	1	1	0 (0%)	0 (0%)	0 (0%)	1	1	1	1
CUL3	2 (1.52%)	1 (0.38%)	0 (0%)	0.2587	1	0.4982	1	0 (0%)	0 (0%)	0 (0%)	1	1	1	1	0 (0%)	0 (0%)	0 (0%)	1	1	1	1	0 (0%)	0 (0%)	0 (0%)	1	1	1	1
DAXX	4 (3.03%)	2 (0.76%)	0 (0%)	0.0985	0.867225	0.1224	1	0 (0%)	0 (0%)	0 (0%)	1	1	1	1	0 (0%)	0 (0%)	0 (0%)	1	1	1	1	0 (0%)	0 (0%)	0 (0%)	1	1	1	1
DCUN1D1	0 (0%)	1 (0.38%)	0 (0%)	1	1	1	1	0 (0%)	0 (0%)	0 (0%)	1	1	1	1	0 (0%)	0 (0%)	0 (0%)	1	1	1	1	0 (0%)	0 (0%)	0 (0%)	1	1	1	1
DDR2	4 (3.03%)	6 (2.27%)	0 (0%)	0.7369	1	0.1224	1	0 (0%)	0 (0%)	0 (0%)	1	1	1	1	1 (8.33%)	1 (2.78%)	0 (0%)	0.4415	1	1	1	0 (0%)	1 (5.56%)	0 (0%)	1	1	1	1
DICER1	0 (0%)	2 (0.76%)	2 (1.57%)	0.5544	1	0.2395	1	0 (0%)	0 (0%)	0 (0%)	1	1	1	1	0 (0%)	0 (0%)	0 (0%)	1	1	1	1	0 (0%)	0 (0%)	0 (0%)	1	1	1	1
DIS3	2 (1.52%)	3 (1.14%)	0 (0%)	1	1	0.4982	1	0 (0%)	0 (0%)	0 (0%)	1	1	1	1	0 (0%)	0 (0%)	0 (0%)	1	1	1	1	0 (0%)	1 (5.56%)	0 (0%)	1	1	1	1
DNMT1	2 (1.52%)	4 (1.52%)	2 (1.57%)	1	1	1	1	0 (0%)	0 (0%)	0 (0%)	1	1	1	1	0 (0%)	0 (0%)	0 (0%)	1	1	1	1	0 (0%)	0 (0%)	0 (0%)	1	1	1	1
DNMT3A	5 (3.79%)	3 (1.14%)	1 (0.79%)	0.1234	0.895125	0.2138	1	0 (0%)	0 (0%)	0 (0%)	1	1	1	1	0 (0%)	1 (2.78%)	0 (0%)	1	1	1	1	0 (0%)	0 (0%)	0 (0%)	1	1	1	1
DNMT3B	2 (1.52%)	2 (0.76%)	2 (1.57%)	0.6034	1	1	1	0 (0%)	0 (0%)	0 (0%)	1	1	1	1	0 (0%)	0 (0%)	0 (0%)	1	1	1	1	0 (0%)	0 (0%)	0 (0%)	1	1	1	1
DOT1L	3 (2.27%)	8 (3.03%)	1 (0.79%)	0.7582	1	0.6224	1	0 (0%)	0 (0%)	0 (0%)	1	1	1	1	0 (0%)	3 (8.33%)	0 (0%)	0.5629	1	1	1	0 (0%)	1 (5.56%)	0 (0%)	1	1	1	1
E2F3	1 (0.76%)	2 (0.76%)	0 (0%)	1	1	1	1	0 (0%)	0 (0%)	0 (0%)	1	1	1	1	0 (0%)	0 (0%)	0 (0%)	1	1	1	1	0 (0%)	0 (0%)	0 (0%)	1	1	1	1
EED	1 (0.76%)	2 (0.76%)	2 (1.57%)	1	1	0.6164	1	0 (0%)	0 (0%)	0 (0%)	1	1	1	1	0 (0%)	1 (2.78%)	0 (0%)	1	1	1	1	0 (0%)	1 (5.56%)	0 (0%)	1	1	1	1
EGFL7	0 (0%)	1 (0.38%)	0 (0%)	1	1	1	1	0 (0%)	0 (0%)	0 (0%)	1	1	1	1	0 (0%)	0 (0%)	0 (0%)	1	1	1	1	0 (0%)	0 (0%)	0 (0%)	1	1	1	1
EGFR	3 (2.27%)	10 (3.79%)	2 (1.57%)	0.557	1	1	1	1 (20%)	0 (0%)	1 (7.14%)	0.25	1	0.4678	1	1 (8.33%)	2 (5.56%)	0 (0%)	1	1	1	1	1 (16.67%)	1 (5.56%)	1 (20%)	0.4457	1	1	1
EIF1AX	1 (0.76%)	4 (1.52%)	0 (0%)	0.6687	1	1	1	0 (0%)	0 (0%)	0 (0%)	1	1	1	1	0 (0%)	0 (0%)	0 (0%)	1	1	1	1	0 (0%)	0 (0%)	0 (0%)	1	1	1	1
EP300	2 (1.52%)	4 (1.52%)	0 (0%)	1	1	0.4982	1	0 (0%)	0 (0%)	0 (0%)	1	1	1	1	0 (0%)	0 (0%)	0 (0%)	1	1	1	1	0 (0%)	0 (0%)	0 (0%)	1	1	1	1
EPCAM	0 (0%)	1 (0.38%)	0 (0%)	1	1	1	1	0 (0%)	0 (0%)	0 (0%)	1	1	1	1	0 (0%)	0 (0%)	0 (0%)	1	1	1	1	0 (0%)	0 (0%)	0 (0%)	1	1	1	1
EPHA3	1 (0.76%)	2 (0.76%)	0 (0%)	1	1	1	1	0 (0%)	0 (0%)	0 (0%)	1	1	1	1	0 (0%)	0 (0%)	0 (0%)	1	1	1	1	0 (0%)	0 (0%)	0 (0%)	1	1	1	1
EPHA5	0 (0%)	3 (1.14%)	1 (0.79%)	0.5539	1	0.4903	1	0 (0%)	0 (0%)	0 (0%)	1	1	1	1	0 (0%)	0 (0%)	0 (0%)	1	1	1	1	1 (16.67%)	1 (5.56%)	0 (0%)	0.4457	1	1	1
EPHB1	5 (3.79%)	2 (0.76%)	3 (2.36%)	0.0438	0.645172	0.7227	1	0 (0%)	0 (0%)	0 (0%)	1	1	1	1	1 (8.33%)	0 (0%)	0 (0%)	0.25	1	1	1	0 (0%)	1 (5.56%)	0 (0%)	1	1	1	1
ERBB2	25 (18.94%)	28 (10.61%)	15 (11.81%)	0.0279	0.63426	0.1242	1	1 (20%)	1 (6.67%)	2 (14.29%)	0.4474	1	1	1	1 (8.33%)	4 (11.11%)	3 (37.5%)	1	1	0.2553	1	0 (0%)	2 (11.11%)	0 (0%)	1	1	1	1
ERBB3	4 (3.03%)	8 (3.03%)	2 (1.57%)	1	1	0.6842	1	0 (0%)	0 (0%)	0 (0%)	1	1	1	1	0 (0%)	1 (2.78%)	0 (0%)	1	1	1	1	0 (0%)	1 (5.56%)	0 (0%)	1	1	1	1
ERBB4	3 (2.27%)	4 (1.52%)	1 (0.79%)	0.6904	1	0.6224	1	0 (0%)	1 (6.67%)	0 (0%)	1	1	1	1	0 (0%)	1 (2.78%)	0 (0%)	1	1	1	1	0 (0%)	0 (0%)	0 (0%)	1	1	1	1
ERCC2	3 (2.27%)	4 (1.52%)	0 (0%)	0.6904	1	0.2474	1	0 (0%)	0 (0%)	0 (0%)	1	1	1	1	0 (0%)	1 (2.78%)	0 (0%)	1	1	1	1	0 (0%)	1 (5.56%)	0 (0%)	1	1	1	1
ERCC3	2 (1.52%)	2 (0.76%)	0 (0%)	0.6034	1	0.4982	1	0 (0%)	0 (0%)	0 (0%)	1	1	1	1	0 (0%)	0 (0%)	0 (0%)	1	1	1	1	1 (16.67%)	1 (5.56%)	0 (0%)	0.4457	1	1	1
ERCC4	2 (1.52%)	6 (2.27%)	0 (0%)	0.7241	1	0.4982	1	0 (0%)	0 (0%)	0 (0%)	1	1	1	1	0 (0%)	0 (0%)	0 (0%)	1	1	1	1	0 (0%)	0 (0%)	0 (0%)	1	1	1	1
ERCC5	1 (0.76%)	4 (1.52%)	1 (0.79%)	0.6687	1	1	1	0 (0%)	0 (0%)	0 (0%)	1	1	1	1	0 (0%)	0 (0%)	0 (0%)	1	1	1	1	0 (0%)	0 (0%)	0 (0%)	1	1	1	1
ERG	2 (1.52%)	6 (2.27%)	0 (0%)	0.7241	1	0.4982	1	0 (0%)	2 (13.33%)	0 (0%)	1	1	1	1	0 (0%)	2 (5.56%)	0 (0%)	1	1	1	1	0 (0%)	0 (0%)	0 (0%)	1	1	1	1
ESR1	20 (15.15%)	66 (25%)	3 (2.36%)	0.0279	0.63426	3.00E-04	0.1023	3 (60%)	9 (60%)	0 (0%)	1	1	0.0103	1	3 (25%)	12 (33.33%)	0 (0%)	0.728	1	0.2421	1	0 (0%)	1 (5.56%)	0 (0%)	1	1	1	1
ETV1	0 (0%)	2 (0.76%)	0 (0%)	0.5544	1	1	1	0 (0%)	0 (0%)	0 (0%)	1	1	1	1	0 (0%)	0 (0%)	0 (0%)	1	1	1	1	0 (0%)	0 (0%)	0 (0%)	1	1	1	1
ETV6	2 (1.52%)	2 (0.76%)	0 (0%)	0.6034	1	0.4982	1	0 (0%)	0 (0%)	0 (0%)	1	1	1	1	0 (0%)	0 (0%)	0 (0%)	1	1	1	1	0 (0%)	0 (0%)	1 (20%)	1	1	0.4545	1
EZH2	1 (0.76%)	1 (0.38%)	1 (0.79%)	1	1	1	1	1 (20%)	0 (0%)	0 (0%)	0.25	1	0.2632	1	0 (0%)	0 (0%)	0 (0%)	1	1	1	1	0 (0%)	0 (0%)	0 (0%)	1	1	1	1
FAM175A	0 (0%)	0 (0%)	1 (0.79%)	1	1	0.4903	1	0 (0%)	0 (0%)	0 (0%)	1	1	1	1	0 (0%)	0 (0%)	0 (0%)	1	1	1	1	1 (16.67%)	0 (0%)	0 (0%)	0.25	1	1	1
FAM46C	0 (0%)	3 (1.14%)	0 (0%)	0.5539	1	1	1	0 (0%)	0 (0%)	0 (0%)	1	1	1	1	0 (0%)	0 (0%)	0 (0%)	1	1	1	1	0 (0%)	0 (0%)	0 (0%)	1	1	1	1
FANCA	1 (0.76%)	3 (1.14%)	0 (0%)	1	1	1	1	1 (20%)	0 (0%)	0 (0%)	0.25	1	0.2632	1	0 (0%)	2 (5.56%)	0 (0%)	1	1	1	1	0 (0%)	1 (5.56%)	0 (0%)	1	1	1	1
FANCC	2 (1.52%)	0 (0%)	0 (0%)	0.1105	0.867225	0.4982	1	0 (0%)	0 (0%)	0 (0%)	1	1	1	1	0 (0%)	0 (0%)	0 (0%)	1	1	1	1	0 (0%)	0 (0%)	0 (0%)	1	1	1	1
FAT1	12 (9.09%)	13 (4.92%)	2 (1.57%)	0.126	0.895125	0.0109	0.66836	0 (0%)	0 (0%)	0 (0%)	1	1	1	1	1 (8.33%)	2 (5.56%)	0 (0%)	1	1	1	1	0 (0%)	1 (5.56%)	0 (0%)	1	1	1	1
FBXW7	4 (3.03%)	1 (0.38%)	0 (0%)	0.0442	0.645172	0.1224	1	0 (0%)	0 (0%)	0 (0%)	1	1	1	1	0 (0%)	0 (0%)	0 (0%)	1	1	1	1	1 (16.67%)	0 (0%)	1 (20%)	0.25	1	1	1
FGF19	23 (17.42%)	60 (22.73%)	18 (14.17%)	0.2407	1	0.5002	1	3 (60%)	1 (6.67%)	5 (35.71%)	0.032	1	0.6027	1	2 (16.67%)	3 (8.33%)	3 (37.5%)	0.5872	1	0.3473	1	1 (16.67%)	1 (5.56%)	0 (0%)	0.4457	1	1	1
FGF3	22 (16.67%)	56 (21.21%)	17 (13.39%)	0.3482	1	0.4912	1	2 (40%)	0 (0%)	4 (28.57%)	0.0526	1	1	1	2 (16.67%)	2 (5.56%)	2 (25%)	0.2569	1	1	1	1 (16.67%)	1 (5.56%)	0 (0%)	0.4457	1	1	1
FGF4	22 (16.67%)	57 (21.59%)	18 (14.17%)	0.2867	1	0.6095	1	3 (60%)	0 (0%)	5 (35.71%)	0.0088	1	0.6027	1	2 (16.67%)	2 (5.56%)	2 (25%)	0.2569	1	1	1	1 (16.67%)	1 (5.56%)	0 (0%)	0.4457	1	1	1
FGFR1	19 (14.39%)	43 (16.29%)	9 (7.09%)	0.6627	1	0.0718	1	1 (20%)	2 (13.33%)	0 (0%)	1	1	0.2632	1	2 (16.67%)	3 (8.33%)	3 (37.5%)	0.5872	1	0.3473	1	1 (16.67%)	1 (5.56%)	0 (0%)	0.4457	1	1	1
FGFR2	2 (1.52%)	7 (2.65%)	0 (0%)	0.7237	1	0.4982	1	1 (20%)	0 (0%)	0 (0%)	0.25	1	0.2632	1	0 (0%)	0 (0%)	0 (0%)	1	1	1	1	0 (0%)	1 (5.56%)	0 (0%)	1	1	1	1
FGFR3	2 (1.52%)	5 (1.89%)	0 (0%)	1	1	0.4982	1	0 (0%)	0 (0%)	0 (0%)	1	1	1	1	0 (0%)	0 (0%)	0 (0%)	1	1	1	1	0 (0%)	0 (0%)	0 (0%)	1	1	1	1
FGFR4	5 (3.79%)	5 (1.89%)	1 (0.79%)	0.3116	1	0.2138	1	0 (0%)	0 (0%)	0 (0%)	1	1	1	1	0 (0%)	1 (2.78%)	0 (0%)	1	1	1	1	0 (0%)	0 (0%)	0 (0%)	1	1	1	1
FH	3 (2.27%)	5 (1.89%)	1 (0.79%)	1	1	0.6224	1	0 (0%)	0 (0%)	0 (0%)	1	1	1	1	0 (0%)	0 (0%)	0 (0%)	1	1	1	1	0 (0%)	0 (0%)	0 (0%)	1	1	1	1
FLCN	0 (0%)	5 (1.89%)	0 (0%)	0.1742	0.9625	1	1	0 (0%)	0 (0%)	0 (0%)	1	1	1	1	0 (0%)	1 (2.78%)	0 (0%)	1	1	1	1	0 (0%)	1 (5.56%)	0 (0%)	1	1	1	1
FLT1	7 (5.3%)	4 (1.52%)	1 (0.79%)	0.0473	0.645172	0.0665	1	1 (20%)	0 (0%)	0 (0%)	0.25	1	0.2632	1	0 (0%)	0 (0%)	0 (0%)	1	1	1	1	0 (0%)	0 (0%)	0 (0%)	1	1	1	1
FLT3	3 (2.27%)	5 (1.89%)	0 (0%)	1	1	0.2474	1	0 (0%)	1 (6.67%)	0 (0%)	1	1	1	1	0 (0%)	1 (2.78%)	0 (0%)	1	1	1	1	0 (0%)	0 (0%)	0 (0%)	1	1	1	1
FLT4	3 (2.27%)	7 (2.65%)	3 (2.36%)	1	1	1	1	0 (0%)	0 (0%)	0 (0%)	1	1	1	1	0 (0%)	1 (2.78%)	1 (12.5%)	1	1	0.4	1	0 (0%)	0 (0%)	1 (20%)	1	1	0.4545	1
FOXA1	14 (10.61%)	22 (8.33%)	10 (7.87%)	0.4629	1	0.5231	1	1 (20%)	2 (13.33%)	0 (0%)	1	1	0.2632	1	1 (8.33%)	2 (5.56%)	1 (12.5%)	1	1	1	1	0 (0%)	0 (0%)	0 (0%)	1	1	1	1
FOXL2	0 (0%)	1 (0.38%)	0 (0%)	1	1	1	1	0 (0%)	0 (0%)	0 (0%)	1	1	1	1	0 (0%)	0 (0%)	0 (0%)	1	1	1	1	0 (0%)	0 (0%)	0 (0%)	1	1	1	1
FOXP1	3 (2.27%)	5 (1.89%)	0 (0%)	1	1	0.2474	1	0 (0%)	0 (0%)	0 (0%)	1	1	1	1	0 (0%)	0 (0%)	0 (0%)	1	1	1	1	0 (0%)	0 (0%)	0 (0%)	1	1	1	1
FUBP1	1 (0.76%)	1 (0.38%)	0 (0%)	1	1	1	1	0 (0%)	0 (0%)	0 (0%)	1	1	1	1	0 (0%)	0 (0%)	0 (0%)	1	1	1	1	0 (0%)	0 (0%)	0 (0%)	1	1	1	1
GATA1	1 (0.76%)	3 (1.14%)	0 (0%)	1	1	1	1	0 (0%)	0 (0%)	0 (0%)	1	1	1	1	0 (0%)	0 (0%)	0 (0%)	1	1	1	1	0 (0%)	0 (0%)	0 (0%)	1	1	1	1
GATA2	0 (0%)	2 (0.76%)	1 (0.79%)	0.5544	1	0.4903	1	0 (0%)	0 (0%)	0 (0%)	1	1	1	1	0 (0%)	1 (2.78%)	0 (0%)	1	1	1	1	0 (0%)	0 (0%)	0 (0%)	1	1	1	1
GATA3	9 (6.82%)	53 (20.08%)	4 (3.15%)	4.00E-04	0.059675	0.2557	1	3 (60%)	8 (53.33%)	5 (35.71%)	1	1	0.6027	1	0 (0%)	7 (19.44%)	2 (25%)	0.1686	1	0.1474	1	0 (0%)	3 (16.67%)	0 (0%)	0.5464	1	1	1
GNA11	0 (0%)	4 (1.52%)	0 (0%)	0.306	1	1	1	0 (0%)	0 (0%)	0 (0%)	1	1	1	1	0 (0%)	0 (0%)	0 (0%)	1	1	1	1	0 (0%)	0 (0%)	0 (0%)	1	1	1	1
GNAQ	1 (0.76%)	1 (0.38%)	0 (0%)	1	1	1	1	0 (0%)	0 (0%)	0 (0%)	1	1	1	1	0 (0%)	0 (0%)	0 (0%)	1	1	1	1	0 (0%)	0 (0%)	0 (0%)	1	1	1	1
GNAS	4 (3.03%)	18 (6.82%)	2 (1.57%)	0.1627	0.9625	0.6842	1	0 (0%)	0 (0%)	1 (7.14%)	1	1	1	1	0 (0%)	0 (0%)	0 (0%)	1	1	1	1	0 (0%)	0 (0%)	0 (0%)	1	1	1	1
GREM1	1 (0.76%)	1 (0.38%)	0 (0%)	1	1	1	1	0 (0%)	0 (0%)	0 (0%)	1	1	1	1	0 (0%)	0 (0%)	0 (0%)	1	1	1	1	0 (0%)	0 (0%)	0 (0%)	1	1	1	1
GRIN2A	5 (3.79%)	4 (1.52%)	3 (2.36%)	0.1671	0.9625	0.7227	1	0 (0%)	0 (0%)	0 (0%)	1	1	1	1	0 (0%)	0 (0%)	0 (0%)	1	1	1	1	0 (0%)	0 (0%)	0 (0%)	1	1	1	1
GSK3B	0 (0%)	4 (1.52%)	0 (0%)	0.306	1	1	1	0 (0%)	0 (0%)	0 (0%)	1	1	1	1	0 (0%)	0 (0%)	0 (0%)	1	1	1	1	0 (0%)	0 (0%)	0 (0%)	1	1	1	1
H3F3C	0 (0%)	2 (0.76%)	1 (0.79%)	0.5544	1	0.4903	1	0 (0%)	0 (0%)	0 (0%)	1	1	1	1	0 (0%)	0 (0%)	0 (0%)	1	1	1	1	0 (0%)	0 (0%)	0 (0%)	1	1	1	1
HGF	2 (1.52%)	2 (0.76%)	0 (0%)	0.6034	1	0.4982	1	0 (0%)	0 (0%)	0 (0%)	1	1	1	1	0 (0%)	0 (0%)	0 (0%)	1	1	1	1	0 (0%)	0 (0%)	0 (0%)	1	1	1	1
HIST1H1C	4 (3.03%)	0 (0%)	0 (0%)	0.012	0.341	0.1224	1	0 (0%)	0 (0%)	0 (0%)	1	1	1	1	0 (0%)	0 (0%)	0 (0%)	1	1	1	1	0 (0%)	0 (0%)	0 (0%)	1	1	1	1
HIST1H2BD	0 (0%)	0 (0%)	0 (0%)	1	1	1	1	0 (0%)	0 (0%)	0 (0%)	1	1	1	1	1 (8.33%)	0 (0%)	0 (0%)	0.25	1	1	1	0 (0%)	0 (0%)	0 (0%)	1	1	1	1
HIST1H3B	2 (1.52%)	2 (0.76%)	0 (0%)	0.6034	1	0.4982	1	0 (0%)	0 (0%)	0 (0%)	1	1	1	1	0 (0%)	0 (0%)	0 (0%)	1	1	1	1	0 (0%)	0 (0%)	0 (0%)	1	1	1	1
HNF1A	3 (2.27%)	3 (1.14%)	1 (0.79%)	0.4049	1	0.6224	1	0 (0%)	0 (0%)	0 (0%)	1	1	1	1	1 (8.33%)	1 (2.78%)	0 (0%)	0.4415	1	1	1	0 (0%)	2 (11.11%)	0 (0%)	1	1	1	1
HRAS	0 (0%)	2 (0.76%)	0 (0%)	0.5544	1	1	1	0 (0%)	0 (0%)	0 (0%)	1	1	1	1	0 (0%)	0 (0%)	0 (0%)	1	1	1	1	0 (0%)	0 (0%)	0 (0%)	1	1	1	1
ICOSLG	0 (0%)	5 (1.89%)	0 (0%)	0.1742	0.9625	1	1	0 (0%)	0 (0%)	0 (0%)	1	1	1	1	1 (8.33%)	0 (0%)	0 (0%)	0.25	1	1	1	0 (0%)	0 (0%)	0 (0%)	1	1	1	1
IDH1	0 (0%)	3 (1.14%)	0 (0%)	0.5539	1	1	1	0 (0%)	0 (0%)	0 (0%)	1	1	1	1	0 (0%)	0 (0%)	0 (0%)	1	1	1	1	0 (0%)	1 (5.56%)	0 (0%)	1	1	1	1
IDH2	1 (0.76%)	1 (0.38%)	0 (0%)	1	1	1	1	0 (0%)	0 (0%)	0 (0%)	1	1	1	1	0 (0%)	0 (0%)	0 (0%)	1	1	1	1	0 (0%)	0 (0%)	0 (0%)	1	1	1	1
IFNGR1	0 (0%)	4 (1.52%)	0 (0%)	0.306	1	1	1	0 (0%)	0 (0%)	0 (0%)	1	1	1	1	1 (8.33%)	0 (0%)	0 (0%)	0.25	1	1	1	1 (16.67%)	0 (0%)	0 (0%)	0.25	1	1	1
IGF1	1 (0.76%)	1 (0.38%)	0 (0%)	1	1	1	1	0 (0%)	0 (0%)	0 (0%)	1	1	1	1	0 (0%)	0 (0%)	0 (0%)	1	1	1	1	0 (0%)	0 (0%)	0 (0%)	1	1	1	1
IGF1R	0 (0%)	18 (6.82%)	1 (0.79%)	0.0012	0.08184	0.4903	1	0 (0%)	1 (6.67%)	0 (0%)	1	1	1	1	0 (0%)	2 (5.56%)	0 (0%)	1	1	1	1	0 (0%)	2 (11.11%)	0 (0%)	1	1	1	1
IGF2	0 (0%)	0 (0%)	0 (0%)	1	1	1	1	0 (0%)	0 (0%)	0 (0%)	1	1	1	1	0 (0%)	0 (0%)	0 (0%)	1	1	1	1	0 (0%)	0 (0%)	0 (0%)	1	1	1	1
IKBKE	8 (6.06%)	13 (4.92%)	2 (1.57%)	0.6396	1	0.1032	1	0 (0%)	0 (0%)	1 (7.14%)	1	1	1	1	0 (0%)	0 (0%)	0 (0%)	1	1	1	1	0 (0%)	0 (0%)	0 (0%)	1	1	1	1
IKZF1	2 (1.52%)	4 (1.52%)	3 (2.36%)	1	1	0.6791	1	0 (0%)	0 (0%)	0 (0%)	1	1	1	1	0 (0%)	0 (0%)	0 (0%)	1	1	1	1	0 (0%)	0 (0%)	0 (0%)	1	1	1	1
IL10	7 (5.3%)	11 (4.17%)	1 (0.79%)	0.6152	1	0.0665	1	0 (0%)	0 (0%)	1 (7.14%)	1	1	1	1	0 (0%)	0 (0%)	0 (0%)	1	1	1	1	0 (0%)	0 (0%)	0 (0%)	1	1	1	1
IL7R	1 (0.76%)	3 (1.14%)	0 (0%)	1	1	1	1	0 (0%)	0 (0%)	0 (0%)	1	1	1	1	0 (0%)	0 (0%)	0 (0%)	1	1	1	1	0 (0%)	0 (0%)	0 (0%)	1	1	1	1
INPP4A	3 (2.27%)	3 (1.14%)	1 (0.79%)	0.4049	1	0.6224	1	0 (0%)	0 (0%)	0 (0%)	1	1	1	1	0 (0%)	0 (0%)	1 (12.5%)	1	1	0.4	1	0 (0%)	0 (0%)	0 (0%)	1	1	1	1
INPP4B	2 (1.52%)	0 (0%)	1 (0.79%)	0.1105	0.867225	1	1	0 (0%)	0 (0%)	0 (0%)	1	1	1	1	0 (0%)	0 (0%)	0 (0%)	1	1	1	1	0 (0%)	0 (0%)	0 (0%)	1	1	1	1
INSR	2 (1.52%)	2 (0.76%)	0 (0%)	0.6034	1	0.4982	1	0 (0%)	0 (0%)	1 (7.14%)	1	1	1	1	0 (0%)	1 (2.78%)	0 (0%)	1	1	1	1	0 (0%)	0 (0%)	0 (0%)	1	1	1	1
IRF4	2 (1.52%)	2 (0.76%)	1 (0.79%)	0.6034	1	1	1	0 (0%)	0 (0%)	0 (0%)	1	1	1	1	0 (0%)	0 (0%)	0 (0%)	1	1	1	1	0 (0%)	0 (0%)	0 (0%)	1	1	1	1
IRS1	2 (1.52%)	5 (1.89%)	0 (0%)	1	1	0.4982	1	0 (0%)	0 (0%)	0 (0%)	1	1	1	1	0 (0%)	1 (2.78%)	0 (0%)	1	1	1	1	1 (16.67%)	0 (0%)	0 (0%)	0.25	1	1	1
IRS2	2 (1.52%)	5 (1.89%)	0 (0%)	1	1	0.4982	1	0 (0%)	1 (6.67%)	0 (0%)	1	1	1	1	0 (0%)	2 (5.56%)	0 (0%)	1	1	1	1	0 (0%)	1 (5.56%)	0 (0%)	1	1	1	1
JAK1	3 (2.27%)	7 (2.65%)	1 (0.79%)	1	1	0.6224	1	0 (0%)	1 (6.67%)	0 (0%)	1	1	1	1	0 (0%)	0 (0%)	0 (0%)	1	1	1	1	0 (0%)	0 (0%)	0 (0%)	1	1	1	1
JAK2	2 (1.52%)	6 (2.27%)	1 (0.79%)	0.7241	1	1	1	0 (0%)	0 (0%)	0 (0%)	1	1	1	1	0 (0%)	1 (2.78%)	0 (0%)	1	1	1	1	0 (0%)	0 (0%)	0 (0%)	1	1	1	1
JAK3	4 (3.03%)	4 (1.52%)	1 (0.79%)	0.4492	1	0.3706	1	0 (0%)	0 (0%)	0 (0%)	1	1	1	1	0 (0%)	0 (0%)	0 (0%)	1	1	1	1	0 (0%)	1 (5.56%)	0 (0%)	1	1	1	1
JUN	1 (0.76%)	3 (1.14%)	1 (0.79%)	1	1	1	1	0 (0%)	0 (0%)	0 (0%)	1	1	1	1	0 (0%)	0 (0%)	0 (0%)	1	1	1	1	0 (0%)	0 (0%)	0 (0%)	1	1	1	1
KDM5A	3 (2.27%)	7 (2.65%)	1 (0.79%)	1	1	0.6224	1	0 (0%)	0 (0%)	0 (0%)	1	1	1	1	0 (0%)	2 (5.56%)	0 (0%)	1	1	1	1	0 (0%)	1 (5.56%)	1 (20%)	1	1	0.4545	1
KDM5C	2 (1.52%)	8 (3.03%)	1 (0.79%)	0.5066	1	1	1	0 (0%)	0 (0%)	0 (0%)	1	1	1	1	0 (0%)	1 (2.78%)	1 (12.5%)	1	1	0.4	1	0 (0%)	0 (0%)	0 (0%)	1	1	1	1
KDM6A	2 (1.52%)	10 (3.79%)	2 (1.57%)	0.3513	1	1	1	0 (0%)	1 (6.67%)	0 (0%)	1	1	1	1	1 (8.33%)	1 (2.78%)	0 (0%)	0.4415	1	1	1	0 (0%)	1 (5.56%)	0 (0%)	1	1	1	1
KDR	3 (2.27%)	4 (1.52%)	0 (0%)	0.6904	1	0.2474	1	0 (0%)	1 (6.67%)	0 (0%)	1	1	1	1	2 (16.67%)	1 (2.78%)	0 (0%)	0.1501	1	0.4947	1	0 (0%)	0 (0%)	0 (0%)	1	1	1	1
KEAP1	1 (0.76%)	2 (0.76%)	0 (0%)	1	1	1	1	0 (0%)	0 (0%)	0 (0%)	1	1	1	1	0 (0%)	1 (2.78%)	0 (0%)	1	1	1	1	0 (0%)	0 (0%)	0 (0%)	1	1	1	1
KIT	4 (3.03%)	1 (0.38%)	0 (0%)	0.0442	0.645172	0.1224	1	0 (0%)	1 (6.67%)	0 (0%)	1	1	1	1	1 (8.33%)	1 (2.78%)	0 (0%)	0.4415	1	1	1	0 (0%)	0 (0%)	0 (0%)	1	1	1	1
KLF4	0 (0%)	2 (0.76%)	1 (0.79%)	0.5544	1	0.4903	1	0 (0%)	0 (0%)	0 (0%)	1	1	1	1	0 (0%)	0 (0%)	0 (0%)	1	1	1	1	1 (16.67%)	0 (0%)	0 (0%)	0.25	1	1	1
KMT2A	0 (0%)	0 (0%)	0 (0%)	1	1	1	1	0 (0%)	0 (0%)	0 (0%)	1	1	1	1	0 (0%)	0 (0%)	0 (0%)	1	1	1	1	0 (0%)	0 (0%)	0 (0%)	1	1	1	1
KMT2C	1 (0.76%)	0 (0%)	3 (2.36%)	0.3333	1	0.3626	1	0 (0%)	0 (0%)	0 (0%)	1	1	1	1	1 (8.33%)	0 (0%)	0 (0%)	0.25	1	1	1	0 (0%)	0 (0%)	0 (0%)	1	1	1	1
KMT2D	0 (0%)	0 (0%)	0 (0%)	1	1	1	1	0 (0%)	0 (0%)	0 (0%)	1	1	1	1	0 (0%)	0 (0%)	0 (0%)	1	1	1	1	0 (0%)	0 (0%)	0 (0%)	1	1	1	1
KRAS	2 (1.52%)	2 (0.76%)	1 (0.79%)	0.6034	1	1	1	0 (0%)	0 (0%)	0 (0%)	1	1	1	1	0 (0%)	0 (0%)	0 (0%)	1	1	1	1	0 (0%)	0 (0%)	0 (0%)	1	1	1	1
LATS1	4 (3.03%)	2 (0.76%)	0 (0%)	0.0985	0.867225	0.1224	1	0 (0%)	0 (0%)	0 (0%)	1	1	1	1	1 (8.33%)	1 (2.78%)	0 (0%)	0.4415	1	1	1	0 (0%)	0 (0%)	0 (0%)	1	1	1	1
LATS2	5 (3.79%)	4 (1.52%)	0 (0%)	0.1671	0.9625	0.0603	1	0 (0%)	0 (0%)	0 (0%)	1	1	1	1	0 (0%)	1 (2.78%)	0 (0%)	1	1	1	1	0 (0%)	1 (5.56%)	0 (0%)	1	1	1	1
LMO1	1 (0.76%)	1 (0.38%)	1 (0.79%)	1	1	1	1	0 (0%)	0 (0%)	0 (0%)	1	1	1	1	0 (0%)	0 (0%)	0 (0%)	1	1	1	1	0 (0%)	0 (0%)	0 (0%)	1	1	1	1
MAP2K1	1 (0.76%)	0 (0%)	0 (0%)	0.3333	1	1	1	0 (0%)	0 (0%)	0 (0%)	1	1	1	1	0 (0%)	0 (0%)	0 (0%)	1	1	1	1	0 (0%)	0 (0%)	0 (0%)	1	1	1	1
MAP2K2	3 (2.27%)	1 (0.38%)	0 (0%)	0.11	0.867225	0.2474	1	0 (0%)	0 (0%)	1 (7.14%)	1	1	1	1	0 (0%)	0 (0%)	0 (0%)	1	1	1	1	0 (0%)	0 (0%)	0 (0%)	1	1	1	1
MAP2K4	7 (5.3%)	21 (7.95%)	5 (3.94%)	0.4086	1	0.7695	1	1 (20%)	2 (13.33%)	0 (0%)	1	1	0.2632	1	0 (0%)	3 (8.33%)	0 (0%)	0.5629	1	1	1	0 (0%)	0 (0%)	0 (0%)	1	1	1	1
MAP3K1	13 (9.85%)	33 (12.5%)	13 (10.24%)	0.5077	1	1	1	1 (20%)	5 (33.33%)	1 (7.14%)	1	1	0.4678	1	0 (0%)	6 (16.67%)	1 (12.5%)	0.3145	1	0.4	1	1 (16.67%)	3 (16.67%)	0 (0%)	1	1	1	1

MAP3K13	2 (1.52%)	3 (1.14%)	0 (0%)	1	1	0.4982	1	0 (0%)	0 (0%)	0 (0%)	1	1	1	1	0 (0%)	0 (0%)	0 (0%)	1	1	1	1	0 (0%)	0 (0%)	0 (0%)	1	1	1	1
MAPK1	0 (0%)	1 (0.38%)	1 (0.79%)	1	1	0.4903	1	0 (0%)	0 (0%)	0 (0%)	1	1	1	1	0 (0%)	0 (0%)	0 (0%)	1	1	1	1	0 (0%)	0 (0%)	0 (0%)	1	1	1	1
MAX	0 (0%)	2 (0.76%)	1 (0.79%)	0.5544	1	0.4903	1	0 (0%)	1 (6.67%)	0 (0%)	1	1	1	1	0 (0%)	0 (0%)	0 (0%)	1	1	1	1	0 (0%)	1 (5.56%)	0 (0%)	1	1	1	1
MCL1	6 (4.55%)	7 (2.65%)	3 (2.36%)	0.3726	1	0.5008	1	0 (0%)	0 (0%)	0 (0%)	1	1	1	1	0 (0%)	0 (0%)	0 (0%)	1	1	1	1	0 (0%)	0 (0%)	0 (0%)	1	1	1	1
MDC1	2 (1.52%)	4 (1.52%)	0 (0%)	1	1	0.4982	1	0 (0%)	0 (0%)	0 (0%)	1	1	1	1	0 (0%)	0 (0%)	0 (0%)	1	1	1	1	0 (0%)	1 (5.56%)	0 (0%)	1	1	1	1
MDM2	8 (6.06%)	13 (4.92%)	3 (2.36%)	0.6396	1	0.2174	1	0 (0%)	0 (0%)	1 (7.14%)	1	1	1	1	1 (8.33%)	0 (0%)	1 (12.5%)	0.25	1	1	1	0 (0%)	0 (0%)	0 (0%)	1	1	1	1
MDM4	10 (7.58%)	14 (5.3%)	1 (0.79%)	0.3783	1	0.0103	0.66836	0 (0%)	1 (6.67%)	1 (7.14%)	1	1	1	1	0 (0%)	0 (0%)	0 (0%)	1	1	1	1	0 (0%)	0 (0%)	0 (0%)	1	1	1	1
MED12	4 (3.03%)	5 (1.89%)	4 (3.15%)	0.4887	1	1	1	0 (0%)	0 (0%)	0 (0%)	1	1	1	1	0 (0%)	0 (0%)	0 (0%)	1	1	1	1	0 (0%)	0 (0%)	0 (0%)	1	1	1	1
MEF2B	0 (0%)	1 (0.38%)	0 (0%)	1	1	1	1	0 (0%)	0 (0%)	0 (0%)	1	1	1	1	0 (0%)	0 (0%)	0 (0%)	1	1	1	1	0 (0%)	0 (0%)	0 (0%)	1	1	1	1
MEN1	3 (2.27%)	8 (3.03%)	2 (1.57%)	0.7582	1	1	1	0 (0%)	0 (0%)	0 (0%)	1	1	1	1	0 (0%)	1 (2.78%)	1 (12.5%)	1	1	0.4	1	0 (0%)	1 (5.56%)	0 (0%)	1	1	1	1
MET	1 (0.76%)	4 (1.52%)	0 (0%)	0.6687	1	1	1	0 (0%)	0 (0%)	0 (0%)	1	1	1	1	0 (0%)	0 (0%)	0 (0%)	1	1	1	1	0 (0%)	0 (0%)	0 (0%)	1	1	1	1
MITF	2 (1.52%)	0 (0%)	0 (0%)	0.1105	0.867225	0.4982	1	0 (0%)	0 (0%)	0 (0%)	1	1	1	1	0 (0%)	0 (0%)	0 (0%)	1	1	1	1	0 (0%)	0 (0%)	0 (0%)	1	1	1	1
MLH1	2 (1.52%)	2 (0.76%)	2 (1.57%)	0.6034	1	1	1	0 (0%)	0 (0%)	0 (0%)	1	1	1	1	0 (0%)	0 (0%)	0 (0%)	1	1	1	1	0 (0%)	0 (0%)	0 (0%)	1	1	1	1
MPL	2 (1.52%)	2 (0.76%)	0 (0%)	0.6034	1	0.4982	1	0 (0%)	0 (0%)	0 (0%)	1	1	1	1	0 (0%)	1 (2.78%)	0 (0%)	1	1	1	1	0 (0%)	1 (5.56%)	0 (0%)	1	1	1	1
MRE11A	0 (0%)	3 (1.14%)	0 (0%)	0.5539	1	1	1	0 (0%)	0 (0%)	0 (0%)	1	1	1	1	0 (0%)	0 (0%)	0 (0%)	1	1	1	1	0 (0%)	0 (0%)	0 (0%)	1	1	1	1
MSH2	2 (1.52%)	0 (0%)	0 (0%)	0.1105	0.867225	0.4982	1	0 (0%)	0 (0%)	0 (0%)	1	1	1	1	0 (0%)	0 (0%)	0 (0%)	1	1	1	1	0 (0%)	0 (0%)	0 (0%)	1	1	1	1
MSH6	1 (0.76%)	3 (1.14%)	0 (0%)	1	1	1	1	0 (0%)	0 (0%)	0 (0%)	1	1	1	1	0 (0%)	0 (0%)	0 (0%)	1	1	1	1	0 (0%)	0 (0%)	0 (0%)	1	1	1	1
MTOR	7 (5.3%)	2 (0.76%)	2 (1.57%)	0.0076	0.2879556	0.1728	1	0 (0%)	0 (0%)	0 (0%)	1	1	1	1	0 (0%)	0 (0%)	0 (0%)	1	1	1	1	0 (0%)	0 (0%)	0 (0%)	1	1	1	1
MUTYH	0 (0%)	3 (1.14%)	0 (0%)	0.5539	1	1	1	0 (0%)	0 (0%)	0 (0%)	1	1	1	1	0 (0%)	1 (2.78%)	0 (0%)	1	1	1	1	0 (0%)	1 (5.56%)	0 (0%)	1	1	1	1
MYC	7 (5.3%)	20 (7.58%)	4 (3.15%)	0.5267	1	0.5408	1	1 (20%)	0 (0%)	1 (7.14%)	0.25	1	0.4678	1	0 (0%)	0 (0%)	3 (37.5%)	1	1	0.0491	1	1 (16.67%)	0 (0%)	0 (0%)	0.25	1	1	1
MYCL1	3 (2.27%)	2 (0.76%)	0 (0%)	0.3386	1	0.2474	1	0 (0%)	0 (0%)	0 (0%)	1	1	1	1	0 (0%)	0 (0%)	0 (0%)	1	1	1	1	0 (0%)	0 (0%)	0 (0%)	1	1	1	1
MYCN	0 (0%)	0 (0%)	0 (0%)	1	1	1	1	0 (0%)	0 (0%)	0 (0%)	1	1	1	1	0 (0%)	0 (0%)	1 (12.5%)	1	1	0.4	1	0 (0%)	0 (0%)	0 (0%)	1	1	1	1
MYD88	2 (1.52%)	0 (0%)	0 (0%)	0.1105	0.867225	0.4982	1	0 (0%)	0 (0%)	0 (0%)	1	1	1	1	0 (0%)	0 (0%)	0 (0%)	1	1	1	1	0 (0%)	0 (0%)	0 (0%)	1	1	1	1
MYOD1	0 (0%)	1 (0.38%)	2 (1.57%)	1	1	0.2395	1	0 (0%)	0 (0%)	0 (0%)	1	1	1	1	0 (0%)	0 (0%)	0 (0%)	1	1	1	1	0 (0%)	0 (0%)	0 (0%)	1	1	1	1
NBN	6 (4.55%)	16 (6.06%)	2 (1.57%)	0.6455	1	0.2821	1	0 (0%)	0 (0%)	1 (7.14%)	1	1	1	1	0 (0%)	0 (0%)	1 (12.5%)	1	1	0.4	1	1 (16.67%)	0 (0%)	0 (0%)	0.25	1	1	1
NCOR1	12 (9.09%)	7 (2.65%)	8 (6.3%)	0.0102	0.3162	0.4877	1	2 (40%)	0 (0%)	0 (0%)	0.0526	1	0.0585	1	1 (8.33%)	2 (5.56%)	1 (12.5%)	1	1	1	1	0 (0%)	1 (5.56%)	0 (0%)	1	1	1	1
NF1	11 (8.33%)	13 (4.92%)	2 (1.57%)	0.1866	0.9789323	0.0196	0.66836	0 (0%)	1 (6.67%)	0 (0%)	1	1	1	1	1 (8.33%)	1 (2.78%)	0 (0%)	0.4415	1	1	1	1 (16.67%)	0 (0%)	1 (20%)	0.25	1	1	1
NF2	0 (0%)	3 (1.14%)	0 (0%)	0.5539	1	1	1	0 (0%)	0 (0%)	0 (0%)	1	1	1	1	0 (0%)	0 (0%)	0 (0%)	1	1	1	1	0 (0%)	1 (5.56%)	0 (0%)	1	1	1	1
NFE2L2	2 (1.52%)	4 (1.52%)	1 (0.79%)	1	1	1	1	0 (0%)	0 (0%)	0 (0%)	1	1	1	1	0 (0%)	0 (0%)	0 (0%)	1	1	1	1	0 (0%)	1 (5.56%)	0 (0%)	1	1	1	1
NKX2.1	0 (0%)	5 (1.89%)	0 (0%)	0.1742	0.9625	1	1	0 (0%)	0 (0%)	0 (0%)	1	1	1	1	1 (8.33%)	2 (5.56%)	0 (0%)	1	1	1	1	0 (0%)	0 (0%)	0 (0%)	1	1	1	1
NKX3.1	1 (0.76%)	2 (0.76%)	0 (0%)	1	1	1	1	0 (0%)	0 (0%)	0 (0%)	1	1	1	1	0 (0%)	0 (0%)	0 (0%)	1	1	1	1	0 (0%)	0 (0%)	0 (0%)	1	1	1	1
NOTCH1	4 (3.03%)	4 (1.52%)	4 (3.15%)	0.4492	1	1	1	0 (0%)	0 (0%)	0 (0%)	1	1	1	1	1 (8.33%)	0 (0%)	0 (0%)	0.25	1	1	1	0 (0%)	0 (0%)	0 (0%)	1	1	1	1
NOTCH2	5 (3.79%)	11 (4.17%)	2 (1.57%)	1	1	0.4473	1	0 (0%)	2 (13.33%)	1 (7.14%)	1	1	1	1	0 (0%)	2 (5.56%)	0 (0%)	1	1	1	1	0 (0%)	1 (5.56%)	0 (0%)	1	1	1	1
NOTCH3	4 (3.03%)	3 (1.14%)	0 (0%)	0.2283	1	0.1224	1	0 (0%)	1 (6.67%)	0 (0%)	1	1	1	1	0 (0%)	0 (0%)	0 (0%)	1	1	1	1	0 (0%)	0 (0%)	1 (20%)	1	1	0.4545	1
NOTCH4	7 (5.3%)	4 (1.52%)	2 (1.57%)	0.0473	0.645172	0.1728	1	0 (0%)	0 (0%)	0 (0%)	1	1	1	1	0 (0%)	0 (0%)	0 (0%)	1	1	1	1	0 (0%)	0 (0%)	0 (0%)	1	1	1	1
NPM1	0 (0%)	0 (0%)	1 (0.79%)	1	1	0.4903	1	0 (0%)	0 (0%)	0 (0%)	1	1	1	1	0 (0%)	0 (0%)	0 (0%)	1	1	1	1	0 (0%)	0 (0%)	0 (0%)	1	1	1	1
NRAS	0 (0%)	2 (0.76%)	0 (0%)	0.5544	1	1	1	0 (0%)	0 (0%)	0 (0%)	1	1	1	1	0 (0%)	0 (0%)	0 (0%)	1	1	1	1	0 (0%)	0 (0%)	0 (0%)	1	1	1	1
NSD1	4 (3.03%)	8 (3.03%)	1 (0.79%)	1	1	0.3706	1	0 (0%)	0 (0%)	1 (7.14%)	1	1	1	1	0 (0%)	0 (0%)	0 (0%)	1	1	1	1	0 (0%)	0 (0%)	0 (0%)	1	1	1	1
NTRK1	5 (3.79%)	4 (1.52%)	1 (0.79%)	0.1671	0.9625	0.2138	1	0 (0%)	0 (0%)	0 (0%)	1	1	1	1	0 (0%)	0 (0%)	0 (0%)	1	1	1	1	0 (0%)	0 (0%)	0 (0%)	1	1	1	1
NTRK2	2 (1.52%)	2 (0.76%)	1 (0.79%)	0.6034	1	1	1	0 (0%)	0 (0%)	0 (0%)	1	1	1	1	0 (0%)	0 (0%)	0 (0%)	1	1	1	1	0 (0%)	0 (0%)	0 (0%)	1	1	1	1
NTRK3	0 (0%)	4 (1.52%)	0 (0%)	0.306	1	1	1	0 (0%)	0 (0%)	0 (0%)	1	1	1	1	0 (0%)	0 (0%)	0 (0%)	1	1	1	1	0 (0%)	0 (0%)	0 (0%)	1	1	1	1
PAK1	17 (12.88%)	34 (12.88%)	12 (9.45%)	1	1	0.4338	1	1 (20%)	0 (0%)	3 (21.43%)	0.25	1	1	1	1 (8.33%)	1 (2.78%)	1 (12.5%)	0.4415	1	1	1	0 (0%)	1 (5.56%)	0 (0%)	1	1	1	1
PAK7	3 (2.27%)	2 (0.76%)	1 (0.79%)	0.3386	1	0.6224	1	1 (20%)	0 (0%)	0 (0%)	0.25	1	0.2632	1	1 (8.33%)	0 (0%)	0 (0%)	0.25	1	1	1	0 (0%)	0 (0%)	0 (0%)	1	1	1	1
PALB2	2 (1.52%)	4 (1.52%)	2 (1.57%)	1	1	1	1	0 (0%)	0 (0%)	0 (0%)	1	1	1	1	0 (0%)	1 (2.78%)	0 (0%)	1	1	1	1	0 (0%)	0 (0%)	0 (0%)	1	1	1	1
PARK2	0 (0%)	3 (1.14%)	0 (0%)	0.5539	1	1	1	0 (0%)	0 (0%)	0 (0%)	1	1	1	1	0 (0%)	0 (0%)	0 (0%)	1	1	1	1	0 (0%)	0 (0%)	0 (0%)	1	1	1	1
PARP1	9 (6.82%)	6 (2.27%)	1 (0.79%)	0.0461	0.645172	0.0194	0.66836	0 (0%)	1 (6.67%)	0 (0%)	1	1	1	1	0 (0%)	1 (2.78%)	0 (0%)	1	1	1	1	0 (0%)	0 (0%)	0 (0%)	1	1	1	1
PAX5	0 (0%)	3 (1.14%)	0 (0%)	0.5539	1	1	1	0 (0%)	0 (0%)	0 (0%)	1	1	1	1	0 (0%)	2 (5.56%)	0 (0%)	1	1	1	1	0 (0%)	1 (5.56%)	0 (0%)	1	1	1	1
PBRM1	2 (1.52%)	1 (0.38%)	1 (0.79%)	0.2587	1	1	1	0 (0%)	0 (0%)	0 (0%)	1	1	1	1	0 (0%)	1 (2.78%)	0 (0%)	1	1	1	1	0 (0%)	1 (5.56%)	0 (0%)	1	1	1	1
PDCD1	0 (0%)	1 (0.38%)	0 (0%)	1	1	1	1	0 (0%)	0 (0%)	0 (0%)	1	1	1	1	0 (0%)	0 (0%)	0 (0%)	1	1	1	1	0 (0%)	0 (0%)	0 (0%)	1	1	1	1
PDGFRA	5 (3.79%)	5 (1.89%)	0 (0%)	0.3116	1	0.0603	1	0 (0%)	1 (6.67%)	0 (0%)	1	1	1	1	0 (0%)	2 (5.56%)	0 (0%)	1	1	1	1	0 (0%)	0 (0%)	0 (0%)	1	1	1	1
PDGFRB	2 (1.52%)	2 (0.76%)	1 (0.79%)	0.6034	1	1	1	0 (0%)	0 (0%)	0 (0%)	1	1	1	1	0 (0%)	0 (0%)	0 (0%)	1	1	1	1	0 (0%)	0 (0%)	0 (0%)	1	1	1	1
PDPK1	1 (0.76%)	0 (0%)	0 (0%)	0.3333	1	1	1	0 (0%)	0 (0%)	0 (0%)	1	1	1	1	0 (0%)	0 (0%)	0 (0%)	1	1	1	1	0 (0%)	0 (0%)	0 (0%)	1	1	1	1
PHOX2B	0 (0%)	1 (0.38%)	0 (0%)	1	1	1	1	0 (0%)	0 (0%)	0 (0%)	1	1	1	1	0 (0%)	0 (0%)	0 (0%)	1	1	1	1	0 (0%)	0 (0%)	0 (0%)	1	1	1	1
PIK3C2G	5 (3.79%)	4 (1.52%)	0 (0%)	0.1671	0.9625	0.0603	1	1 (20%)	0 (0%)	0 (0%)	0.25	1	0.2632	1	0 (0%)	0 (0%)	0 (0%)	1	1	1	1	0 (0%)	1 (5.56%)	0 (0%)	1	1	1	1
PIK3C3	2 (1.52%)	3 (1.14%)	0 (0%)	1	1	0.4982	1	0 (0%)	0 (0%)	0 (0%)	1	1	1	1	0 (0%)	0 (0%)	0 (0%)	1	1	1	1	0 (0%)	0 (0%)	0 (0%)	1	1	1	1
PIK3CA	68 (51.52%)	89 (33.71%)	73 (57.48%)	7.00E-04	0.059675	0.3827	1	1 (20%)	7 (46.67%)	1 (7.14%)	0.6027	1	0.4678	1	7 (58.33%)	11 (30.56%)	2 (25%)	0.1008	1	0.1968	1	3 (50%)	5 (27.78%)	1 (20%)	0.3618	1	0.5455	1
PIK3CB	2 (1.52%)	3 (1.14%)	1 (0.79%)	1	1	1	1	0 (0%)	0 (0%)	0 (0%)	1	1	1	1	1 (8.33%)	0 (0%)	0 (0%)	0.25	1	1	1	0 (0%)	0 (0%)	0 (0%)	1	1	1	1
PIK3CD	1 (0.76%)	2 (0.76%)	1 (0.79%)	1	1	1	1	0 (0%)	0 (0%)	0 (0%)	1	1	1	1	0 (0%)	0 (0%)	0 (0%)	1	1	1	1	0 (0%)	0 (0%)	0 (0%)	1	1	1	1
PIK3CG	3 (2.27%)	3 (1.14%)	1 (0.79%)	0.4049	1	0.6224	1	0 (0%)	0 (0%)	0 (0%)	1	1	1	1	0 (0%)	2 (5.56%)	0 (0%)	1	1	1	1	0 (0%)	1 (5.56%)	0 (0%)	1	1	1	1
PIK3R1	2 (1.52%)	7 (2.65%)	1 (0.79%)	0.7237	1	1	1	0 (0%)	0 (0%)	0 (0%)	1	1	1	1	0 (0%)	0 (0%)	0 (0%)	1	1	1	1	1 (16.67%)	0 (0%)	0 (0%)	0.25	1	1	1
PIK3R2	1 (0.76%)	3 (1.14%)	0 (0%)	1	1	1	1	0 (0%)	0 (0%)	0 (0%)	1	1	1	1	0 (0%)	0 (0%)	1 (12.5%)	1	1	0.4	1	0 (0%)	0 (0%)	0 (0%)	1	1	1	1
PIK3R3	1 (0.76%)	4 (1.52%)	1 (0.79%)	0.6687	1	1	1	0 (0%)	0 (0%)	0 (0%)	1	1	1	1	0 (0%)	1 (2.78%)	0 (0%)	1	1	1	1	0 (0%)	1 (5.56%)	0 (0%)	1	1	1	1
PIM1	0 (0%)	2 (0.76%)	0 (0%)	0.5544	1	1	1	0 (0%)	0 (0%)	0 (0%)	1	1	1	1	0 (0%)	0 (0%)	0 (0%)	1	1	1	1	0 (0%)	0 (0%)	0 (0%)	1	1	1	1
PLK2	4 (3.03%)	7 (2.65%)	0 (0%)	1	1	0.1224	1	0 (0%)	0 (0%)	0 (0%)	1	1	1	1	0 (0%)	0 (0%)	0 (0%)	1	1	1	1	1 (16.67%)	0 (0%)	0 (0%)	0.25	1	1	1
PMAIP1	0 (0%)	0 (0%)	0 (0%)	1	1	1	1	0 (0%)	0 (0%)	0 (0%)	1	1	1	1	0 (0%)	0 (0%)	0 (0%)	1	1	1	1	0 (0%)	0 (0%)	0 (0%)	1	1	1	1
PMS1	1 (0.76%)	3 (1.14%)	0 (0%)	1	1	1	1	0 (0%)	0 (0%)	0 (0%)	1	1	1	1	1 (8.33%)	0 (0%)	0 (0%)	0.25	1	1	1	0 (0%)	0 (0%)	0 (0%)	1	1	1	1
PMS2	1 (0.76%)	1 (0.38%)	0 (0%)	1	1	1	1	0 (0%)	0 (0%)	0 (0%)	1	1	1	1	0 (0%)	0 (0%)	0 (0%)	1	1	1	1	0 (0%)	0 (0%)	0 (0%)	1	1	1	1
PNRC1	1 (0.76%)	1 (0.38%)	0 (0%)	1	1	1	1	0 (0%)	0 (0%)	0 (0%)	1	1	1	1	0 (0%)	0 (0%)	0 (0%)	1	1	1	1	0 (0%)	0 (0%)	0 (0%)	1	1	1	1
POLE	5 (3.79%)	6 (2.27%)	1 (0.79%)	0.5173	1	0.2138	1	0 (0%)	0 (0%)	0 (0%)	1	1	1	1	0 (0%)	0 (0%)	0 (0%)	1	1	1	1	0 (0%)	1 (5.56%)	0 (0%)	1	1	1	1
PPP2R1A	3 (2.27%)	7 (2.65%)	0 (0%)	1	1	0.2474	1	0 (0%)	1 (6.67%)	0 (0%)	1	1	1	1	0 (0%)	1 (2.78%)	0 (0%)	1	1	1	1	0 (0%)	0 (0%)	0 (0%)	1	1	1	1
PRDM1	1 (0.76%)	8 (3.03%)	1 (0.79%)	0.2823	1	1	1	0 (0%)	1 (6.67%)	0 (0%)	1	1	1	1	0 (0%)	1 (2.78%)	0 (0%)	1	1	1	1	0 (0%)	0 (0%)	0 (0%)	1	1	1	1
PRKAR1A	0 (0%)	9 (3.41%)	0 (0%)	0.0325	0.645172	1	1	0 (0%)	0 (0%)	1 (7.14%)	1	1	1	1	0 (0%)	0 (0%)	1 (12.5%)	1	1	0.4	1	0 (0%)	0 (0%)	0 (0%)	1	1	1	1
PTCH1	4 (3.03%)	4 (1.52%)	1 (0.79%)	0.4492	1	0.3706	1	0 (0%)	1 (6.67%)	0 (0%)	1	1	1	1	0 (0%)	3 (8.33%)	0 (0%)	0.5629	1	1	1	0 (0%)	1 (5.56%)	0 (0%)	1	1	1	1
PTEN	12 (9.09%)	18 (6.82%)	11 (8.66%)	0.4259	1	1	1	0 (0%)	0 (0%)	1 (7.14%)	1	1	1	1	0 (0%)	0 (0%)	0 (0%)	1	1	1	1	1 (16.67%)	1 (5.56%)	2 (40%)	0.4457	1	0.5455	1
PTPN11	1 (0.76%)	0 (0%)	0 (0%)	0.3333	1	1	1	0 (0%)	0 (0%)	0 (0%)	1	1	1	1	0 (0%)	0 (0%)	0 (0%)	1	1	1	1	0 (0%)	0 (0%)	0 (0%)	1	1	1	1
PTPRD	3 (2.27%)	6 (2.27%)	1 (0.79%)	1	1	0.6224	1	0 (0%)	1 (6.67%)	0 (0%)	1	1	1	1	1 (8.33%)	1 (2.78%)	0 (0%)	0.4415	1	1	1	0 (0%)	1 (5.56%)	0 (0%)	1	1	1	1
PTPRS	3 (2.27%)	4 (1.52%)	2 (1.57%)	0.6904	1	1	1	0 (0%)	0 (0%)	0 (0%)	1	1	1	1	1 (8.33%)	0 (0%)	1 (12.5%)	0.25	1	1	1	0 (0%)	0 (0%)	0 (0%)	1	1	1	1
PTPRT	5 (3.79%)	10 (3.79%)	0 (0%)	1	1	0.0603	1	0 (0%)	0 (0%)	0 (0%)	1	1	1	1	0 (0%)	0 (0%)	0 (0%)	1	1	1	1	0 (0%)	1 (5.56%)	0 (0%)	1	1	1	1
RAC1	0 (0%)	2 (0.76%)	0 (0%)	0.5544	1	1	1	0 (0%)	0 (0%)	0 (0%)	1	1	1	1	0 (0%)	1 (2.78%)	0 (0%)	1	1	1	1	0 (0%)	1 (5.56%)	0 (0%)	1	1	1	1
RAD50	3 (2.27%)	5 (1.89%)	1 (0.79%)	1	1	0.6224	1	0 (0%)	0 (0%)	0 (0%)	1	1	1	1	0 (0%)	0 (0%)	0 (0%)	1	1	1	1	1 (16.67%)	0 (0%)	0 (0%)	0.25	1	1	1
RAD51	1 (0.76%)	3 (1.14%)	0 (0%)	1	1	1	1	0 (0%)	0 (0%)	0 (0%)	1	1	1	1	0 (0%)	0 (0%)	0 (0%)	1	1	1	1	0 (0%)	0 (0%)	0 (0%)	1	1	1	1
RAD51B	0 (0%)	1 (0.38%)	0 (0%)	1	1	1	1	1 (20%)	1 (6.67%)	0 (0%)	0.4474	1	0.2632	1	0 (0%)	0 (0%)	0 (0%)	1	1	1	1	0 (0%)	0 (0%)	0 (0%)	1	1	1	1
RAD51C	3 (2.27%)	13 (4.92%)	0 (0%)	0.2824	1	0.2474	1	0 (0%)	1 (6.67%)	2 (14.29%)	1	1	1	1	0 (0%)	1 (2.78%)	1 (12.5%)	1	1	0.4	1	0 (0%)	0 (0%)	0 (0%)	1	1	1	1
RAD51D	0 (0%)	3 (1.14%)	1 (0.79%)	0.5539	1	0.4903	1	0 (0%)	0 (0%)	0 (0%)	1	1	1	1	0 (0%)	1 (2.78%)	1 (12.5%)	1	1	0.4	1	0 (0%)	0 (0%)	0 (0%)	1	1	1	1
RAD52	1 (0.76%)	2 (0.76%)	0 (0%)	1	1	1	1	0 (0%)	0 (0%)	0 (0%)	1	1	1	1	0 (0%)	0 (0%)	0 (0%)	1	1	1	1	0 (0%)	0 (0%)	0 (0%)	1	1	1	1
RAD54L	2 (1.52%)	2 (0.76%)	0 (0%)	0.6034	1	0.4982	1	0 (0%)	0 (0%)	0 (0%)	1	1	1	1	0 (0%)	1 (2.78%)	0 (0%)	1	1	1	1	0 (0%)	1 (5.56%)	0 (0%)	1	1	1	1
RAF1	1 (0.76%)	1 (0.38%)	0 (0%)	1	1	1	1	0 (0%)	0 (0%)	0 (0%)	1	1	1	1	0 (0%)	0 (0%)	0 (0%)	1	1	1	1	0 (0%)	0 (0%)	0 (0%)	1	1	1	1
RARA	4 (3.03%)	7 (2.65%)	2 (1.57%)	1	1	0.6842	1	0 (0%)	1 (6.67%)	2 (14.29%)	1	1	1	1	0 (0%)	1 (2.78%)	1 (12.5%)	1	1	0.4	1	0 (0%)	0 (0%)	0 (0%)	1	1	1	1
RASA1	4 (3.03%)	2 (0.76%)	1 (0.79%)	0.0985	0.867225	0.3706	1	0 (0%)	0 (0%)	0 (0%)	1	1	1	1	0 (0%)	0 (0%)	0 (0%)	1	1	1	1	1 (16.67%)	0 (0%)	0 (0%)	0.25	1	1	1
RB1	6 (4.55%)	5 (1.89%)	1 (0.79%)	0.1912	0.9878667	0.1204	1	0 (0%)	0 (0%)	0 (0%)	1	1	1	1	0 (0%)	0 (0%)	0 (0%)	1	1	1	1	0 (0%)	1 (5.56%)	0 (0%)	1	1	1	1
RBM10	2 (1.52%)	5 (1.89%)	0 (0%)	1	1	0.4982	1	0 (0%)	0 (0%)	0 (0%)	1	1	1	1	0 (0%)	1 (2.78%)	0 (0%)	1	1	1	1	0 (0%)	1 (5.56%)	1 (20%)	1	1	0.4545	1
RECQL4	4 (3.03%)	20 (7.58%)	3 (2.36%)	0.0785	0.867225	1	1	1 (20%)	0 (0%)	0 (0%)	0.25	1	0.2632	1	0 (0%)	0 (0%)	1 (12.5%)	1	1	0.4	1	1 (16.67%)	0 (0%)	0 (0%)	0.25	1	1	1
REL	3 (2.27%)	1 (0.38%)	1 (0.79%)	0.11	0.867225	0.6224	1	0 (0%)	0 (0%)	0 (0%)	1	1	1	1	0 (0%)	0 (0%)	0 (0%)	1	1	1	1	0 (0%)	1 (5.56%)	0 (0%)	1	1	1	1
RET	0 (0%)	4 (1.52%)	2 (1.57%)	0.306	1	0.2395	1	0 (0%)	0 (0%)	0 (0%)	1	1	1	1	0 (0%)	0 (0%)	0 (0%)	1	1	1	1	1 (16.67%)	0 (0%)	0 (0%)	0.25	1	1	1
RFWD2	11 (8.33%)	3 (1.14%)	1 (0.79%)	6.00E-04	0.059675	0.0054	0.66836	0 (0%)	0 (0%)	0 (0%)	1	1	1	1	0 (0%)	1 (2.78%)	0 (0%)	1	1	1	1	0 (0%)	1 (5.56%)	0 (0%)	1	1	1	1
RHOA	5 (3.79%)	3 (1.14%)	2 (1.57%)	0.1234	0.895125	0.4473	1	0 (0%)	0 (0%)	0 (0%)	1	1	1	1	0 (0%)	1 (2.78%)	0 (0%)	1	1	1	1	0 (0%)	1 (5.56%)	0 (0%)	1	1	1	1
RICTOR	6 (4.55%)	9 (3.41%)	3 (2.36%)	0.5845	1	0.5008	1	0 (0%)	0 (0%)	0 (0%)	1	1	1	1	0 (0%)	0 (0%)	0 (0%)	1	1	1	1	1 (16.67%)	0 (0%)	0 (0%)	0.25	1	1	1
RIT1	4 (3.03%)	8 (3.03%)	1 (0.79%)	1	1	0.3706	1	0 (0%)	0 (0%)	0 (0%)	1	1	1	1	0 (0%)	0 (0%)	0 (0%)	1	1	1	1	0 (0%)	0 (0%)	0 (0%)	1	1	1	1
RNF43	2 (1.52%)	9 (3.41%)	0 (0%)	0.3493	1	0.4982	1	0 (0%)	1 (6.67%)	1 (7.14%)	1	1	1	1	1 (8.33%)	1 (2.78%)	0 (0%)	0.4415	1	1	1	0 (0%)	0 (0%)	0 (0%)	1	1	1	1
ROS1	3 (2.27%)	9 (3.41%)	0 (0%)	0.7579	1	0.2474	1	0 (0%)	1 (6.67%)	0 (0%)	1	1	1	1	0 (0%)	2 (5.56%)	0 (0%)	1	1	1	1	0 (0%)	3 (16.67%)	0 (0%)	0.5464	1	1	1
RPS6KA4	2 (1.52%)	1 (0.38%)	1 (0.79%)	0.2587	1	1	1	0 (0%)	0 (0%)	0 (0%)	1	1	1	1	0 (0%)	0 (0%)	1 (12.5%)	1	1	0.4	1	0 (0%)	0 (0%)	0 (0%)	1	1	1	1
RPS6KB2	7 (5.3%)	9 (3.41%)	6 (4.72%)	0.4195	1	1	1	0 (0%)	0 (0%)	1 (7.14%)	1	1	1	1	0 (0%)	1 (2.78%)	0 (0%)	1	1	1	1	0 (0%)	1 (5.56%)	0 (0%)	1	1	1	1
RPTOR	0 (0%)	5 (1.89%)	0 (0%)	0.1742	0.9625	1	1	0 (0%)	0 (0%)	1 (7.14%)	1	1	1	1	0 (0%)	0 (0%)	0 (0%)	1	1	1	1	0 (0%)	0 (0%)	0 (0%)	1	1	1	1
RUNX1	6 (4.55%)	6 (2.27%)	12 (9.45%)	0.2257	1	0.1458	1	0 (0%)	0 (0%)	0 (0%)	1	1	1	1	0 (0%)	1 (2.78%)	0 (0%)	1	1	1	1	0 (0%)	0 (0%)	0 (0%)	1	1	1	1
RYBP	0 (0%)	1 (0.38%)	0 (0%)	1	1	1	1	0 (0%)	0 (0%)	0 (0%)	1	1	1	1	0 (0%)	0 (0%)	0 (0%)	1	1	1	1	0 (0%)	0 (0%)	0 (0%)	1	1	1	1
SDHA	0 (0%)	5 (1.89%)	0 (0%)	0.1742	0.9625	1	1	0 (0%)	0 (0%)	0 (0%)	1	1	1	1	0 (0%)	0 (0%)	0 (0%)	1	1	1	1	0 (0%)	0 (0%)	0 (0%)	1	1	1	1
SDHAF2	1 (0.76%)	0 (0%)	1 (0.79%)	0.3333	1	1	1	0 (0%)	0 (0%)	1 (7.14%)	1	1	1	1	0 (0%)	0 (0%)	0 (0%)	1	1	1	1	0 (0%)	0 (0%)	0 (0%)	1	1	1	1
SDHB	1 (0.76%)	2 (0.76%)	0 (0%)	1	1	1	1	0 (0%)	0 (0%)	0 (0%)	1	1	1	1	0 (0%)	0 (0%)	0 (0%)	1	1	1	1	0 (0%)	0 (0%)	0 (0%)	1	1	1	1
SDHC	4 (3.03%)	2 (0.76%)	0 (0%)	0.0985	0.867225	0.1224	1	0 (0%)	0 (0%)	0 (0%)	1	1	1	1	0 (0%)	1 (2.78%)	0 (0%)	1	1	1	1	0 (0%)	1 (5.56%)	0 (0%)	1	1	1	1
SDHD	0 (0%)	0 (0%)	0 (0%)	1	1	1	1	0 (0%)	0 (0%)	0 (0%)	1	1	1	1	0 (0%)	0 (0%)	0 (0%)	1	1	1	1	0 (0%)	0 (0%)	0 (0%)	1	1	1	1
SETD2	4 (3.03%)	3 (1.14%)	4 (3.15%)	0.2283	1	1	1	0 (0%)	0 (0%)	0 (0%)	1	1	1	1	0 (0%)	1 (2.78%)	1 (12.5%)	1	1	0.4	1	0 (0%)	2 (11.11%)	0 (0%)	1	1	1	1
SF3B1	4 (3.03%)	6 (2.27%)	2 (1.57%)	0.7369	1	0.6842	1	0 (0%)	0 (0%)	0 (0%)	1	1	1	1	0 (0%)	1 (2.78%)	0 (0%)	1	1	1	1	0 (0%)	0 (0%)	0 (0%)	1	1	1	1
SH2D1A	1 (0.76%)	3 (1.14%)	0 (0%)	1	1	1	1	0 (0%)	0 (0%)	0 (0%)	1	1	1	1	0 (0%)	0 (0%)	0 (0%)	1	1	1	1	0 (0%)	0 (0%)	0 (0%)	1	1	1	1
SHQ1	0 (0%)	2 (0.76%)	0 (0%)	0.5544	1	1	1	0 (0%)	0 (0%)	0 (0%)	1	1	1	1	0 (0%)	0 (0%)	0 (0%)	1	1	1	1	0 (0%)	0 (0%)	0 (0%)	1	1	1	1
SMAD2	2 (1.52%)	0 (0%)	0 (0%)	0.1105	0.867225	0.4982	1	0 (0%)	0 (0%)	0 (0%)	1	1	1	1	0 (0%)	0 (0%)	0 (0%)	1	1	1	1	0 (0%)	0 (0%)	0 (0%)	1	1	1	1
SMAD3	0 (0%)	2 (0.76%)	0 (0%)	0.5544	1	1	1	0 (0%)	0 (0%)	0 (0%)	1	1	1	1	0 (0%)	1 (2.78%)	0 (0%)	1	1	1	1	0 (0%)	1 (5.56%)	0 (0%)	1	1	1	1
SMAD4	6 (4.55%)	6 (2.27%)	3 (2.36%)	0.2257	1	0.5008	1	0 (0%)	1 (6.67%)	0 (0%)	1	1	1	1	0 (0%)	1 (2.78%)	0 (0%)	1	1	1	1	0 (0%)	0 (0%)	0 (0%)	1	1	1	1
SMARCA4	2 (1.52%)	2 (0.76%)	1 (0.79%)	0.6034	1	1	1	0 (0%)	0 (0%)	0 (0%)	1	1	1	1	1 (8.33%)	0 (0%)	0 (0%)	0.25	1	1	1	0 (0%)	1 (5.56%)	0 (0%)	1	1	1	1
SMARCB1	2 (1.52%)	0 (0%)	0 (0%)	0.1105	0.867225	0.4982	1	0 (0%)	0 (0%)	0 (0%)	1	1	1	1	0 (0%)	0 (0%)	0 (0%)	1	1	1	1	0 (0%)	0 (0%)	0 (0%)	1	1	1	1
SMARCD1	1 (0.76%)	0 (0%)	1 (0.79%)	0.3333	1	1	1	0 (0%)	0 (0%)	0 (0%)	1	1	1	1	0 (0%)	0 (0%)	0 (0%)	1	1	1	1	0 (0%)	0 (0%)	0 (0%)	1	1	1	1
SMO	2 (1.52%)	1 (0.38%)	2 (1.57%)	0.2587	1	1	1	0 (0%)	0 (0%)	0 (0%)	1	1	1	1	0 (0%)	0 (0%)	0 (0%)	1	1	1	1	0 (0%)	0 (0%)	0 (0%)	1	1	1	1
SOCS1	1 (0.76%)	1 (0.38%)	0 (0%)	1	1	1	1	0 (0%)	0 (0%)	0 (0%)	1	1	1	1	0 (0%)	0 (0%)	0 (0%)	1	1	1	1	0 (0%)	0 (0%)	0 (0%)	1	1	1	1
SOX17	3 (2.27%)	7 (2.65%)	1 (0.79%)	1	1	0.6224	1	0 (0%)	0 (0%)	0 (0%)	1	1	1	1	2 (16.67%)	0 (0%)	1 (12.5%)	0.0585	1	1	1	1 (16.67%)	1 (5.56%)	0 (0%)	0.4457	1	1	1
SOX2	1 (0.76%)	4 (1.52%)	0 (0%)	0.6687	1	1	1	0 (0%)	0 (0%)	0 (0%)	1	1	1	1	0 (0%)	1 (2.78%)	0 (0%)	1	1	1	1	0 (0%)	1 (5.56%)	0 (0%)	1	1	1	1
SOX9	3 (2.27%)	9 (3.41%)	1 (0.79%)	0.7579	1	0.6224	1	0 (0%)	0 (0%)	0 (0%)	1	1	1	1	0 (0%)	1 (2.78%)	0 (0%)	1	1	1	1	2 (33.33%)	1 (5.56%)	0 (0%)	0.1433	1	0.4545	1
SPEN	10 (7.58%)	18 (6.82%)	3 (2.36%)	0.836	1	0.085	1	0 (0%)	2 (13.33%)	0 (0%)	1	1	1	1	0 (0%)	2 (5.56%)	0 (0%)	1	1	1	1	0 (0%)	2 (11.11%)	0 (0%)	1	1	1	1
SPOP	4 (3.03%)	9 (3.41%)	1 (0.79%)	1	1	0.3706	1	0 (0%)	0 (0%)	2 (14.29%)	1	1	1	1	1 (8.33%)	0 (0%)	0 (0%)	0.25	1	1	1	0 (0%)	0 (0%)	0 (0%)	1	1	1	1
SRC	0 (0%)	4 (1.52%)	1 (0.79%)	0.306	1	0.4903	1	0 (0%)	0 (0%)	0 (0%)	1	1	1	1	0 (0%)	0 (0%)	0 (0%)	1	1	1	1	0 (0%)	0 (0%)	0 (0%)	1	1	1	1
STAG2	3 (2.27%)	11 (4.17%)	2 (1.57%)	0.4021	1	1	1	0 (0%)	1 (6.67%)	0 (0%)	1	1	1	1	0 (0%)	4 (11.11%)	0 (0%)	0.5597	1	1	1	0 (0%)	2 (11.11%)	0 (0%)	1	1	1	1
STK11	3 (2.27%)	2 (0.76%)	1 (0.79%)	0.3386	1	0.6224	1	0 (0%)	0 (0%)	1 (7.14%)	1	1	1	1	0 (0%)	0 (0%)	0 (0%)	1	1	1	1	0 (0%)	0 (0%)	0 (0%)	1	1	1	1
STK40	0 (0%)	1 (0.38%)	0 (0%)	1	1	1	1	0 (0%)	0 (0%)	0 (0%)	1	1	1	1	0 (0%)	0 (0%)	0 (0%)	1	1	1	1	0 (0%)	0 (0%)	0 (0%)	1	1	1	1
SUFU	0 (0%)	1 (0.38%)	0 (0%)	1	1	1	1	0 (0%)	0 (0%)	0 (0%)	1	1	1	1	0 (0%)	1 (2.78%)	0 (0%)	1	1	1	1	0 (0%)	0 (0%)	0 (0%)	1	1	1	1
SUZ12	1 (0.76%)	3 (1.14%)	0 (0%)	1	1	1	1	0 (0%)	0 (0%)	0 (0%)	1	1	1	1	0 (0%)	1 (2.78%)	0 (0%)	1	1	1	1	0 (0%)	0 (0%)	0 (0%)	1	1	1	1
SYK	2 (1.52%)	1 (0.38%)	0 (0%)	0.2587	1	0.4982	1	0 (0%)	0 (0%)	0 (0%)	1	1	1	1	0 (0%)	1 (2.78%)	0 (0%)	1	1	1	1	0 (0%)	0 (0%)	0 (0%)	1	1	1	1
TBX3	21 (15.91%)	17 (6.44%)	13 (10.24%)	0.0036	0.1753714	0.2005	1	0 (0%)	1 (6.67%)	0 (0%)	1	1	1	1	0 (0%)	2 (5.56%)	1 (12.5%)	1	1	0.4	1	0 (0%)	2 (11.11%)	0 (0%)	1	1	1	1
TERT	2 (1.52%)	8 (3.03%)	0 (0%)	0.5066	1	0.4982	1	0 (0%)	0 (0%)	0 (0%)	1	1	1	1	1 (8.33%)	0 (0%)	0 (0%)	0.25	1	1	1	1 (16.67%)	0 (0%)	0 (0%)	0.25	1	1	1
TET1	0 (0%)	3 (1.14%)	1 (0.79%)	0.5539	1	0.4903	1	0 (0%)	0 (0%)	0 (0%)	1	1	1	1	1 (8.33%)	1 (2.78%)	0 (0%)	0.4415	1	1	1	1 (16.67%)	2 (11.11%)	0 (0%)	1	1	1	1
TET2	5 (3.79%)	6 (2.27%)	1 (0.79%)	0.5173	1	0.2138	1	0 (0%)	0 (0%)	1 (7.14%)	1	1	1	1	0 (0%)	1 (2.78%)	0 (0%)	1	1	1	1	1 (16.67%)	1 (5.56%)	0 (0%)	0.4457	1	1	1
TGFBR1	0 (0%)	0 (0%)	2 (1.57%)	1	1	0.2395	1	0 (0%)	0 (0%)	0 (0%)	1	1	1	1	0 (0%)	0 (0%)	0 (0%)	1	1	1	1	0 (0%)	0 (0%)	0 (0%)	1	1	1	1
TGFBR2	0 (0%)	0 (0%)	0 (0%)	1	1	1	1	0 (0%)	0 (0%)	0 (0%)	1	1	1	1	0 (0%)	0 (0%)	0 (0%)	1	1	1	1	0 (0%)	0 (0%)	0 (0%)	1	1	1	1
TMEM127	0 (0%)	0 (0%)	0 (0%)	1	1	1	1	0 (0%)	0 (0%)	0 (0%)	1	1	1	1	0 (0%)	0 (0%)	0 (0%)	1	1	1	1	0 (0%)	0 (0%)	0 (0%)	1	1	1	1
TMPRSS2	0 (0%)	6 (2.27%)	0 (0%)	0.1846	0.9789323	1	1	0 (0%)	0 (0%)	0 (0%)	1	1	1	1	0 (0%)	0 (0%)	0 (0%)	1	1	1	1	0 (0%)	0 (0%)	0 (0%)	1	1	1	1
TNFAIP3	1 (0.76%)	3 (1.14%)	0 (0%)	1	1	1	1	0 (0%)	0 (0%)	0 (0%)	1	1	1	1	1 (8.33%)	0 (0%)	0 (0%)	0.25	1	1	1	0 (0%)	0 (0%)	0 (0%)	1	1	1	1
TNFRSF14	0 (0%)	1 (0.38%)	1 (0.79%)	1	1	0.4903	1	0 (0%)	0 (0%)	0 (0%)	1	1	1	1	0 (0%)	0 (0%)	0 (0%)	1	1	1	1	0 (0%)	0 (0%)	0 (0%)	1	1	1	1
TOP1	2 (1.52%)	6 (2.27%)	0 (0%)	0.7241	1	0.4982	1	0 (0%)	0 (0%)	0 (0%)	1	1	1	1	0 (0%)	0 (0%)	0 (0%)	1	1	1	1	0 (0%)	0 (0%)	0 (0%)	1	1	1	1
TP53	26 (19.7%)	90 (34.09%)	11 (8.66%)	0.0033	0.1753714	0.0128	0.66836	1 (20%)	1 (6.67%)	0 (0%)	0.4474	1	0.2632	1	5 (41.67%)	13 (36.11%)	3 (37.5%)	0.7429	1	1	1	5 (83.33%)	13 (72.22%)	5 (100%)	1	1	1	1
TP63	3 (2.27%)	4 (1.52%)	0 (0%)	0.6904	1	0.2474	1	0 (0%)	0 (0%)	0 (0%)	1	1	1	1	0 (0%)	0 (0%)	0 (0%)	1	1	1	1	0 (0%)	0 (0%)	0 (0%)	1	1	1	1
TRAF7	0 (0%)	2 (0.76%)	1 (0.79%)	0.5544	1	0.4903	1	0 (0%)	0 (0%)	0 (0%)	1	1	1	1	0 (0%)	0 (0%)	0 (0%)	1	1	1	1	0 (0%)	1 (5.56%)	0 (0%)	1	1	1	1
TSC1	2 (1.52%)	3 (1.14%)	1 (0.79%)	1	1	1	1	0 (0%)	0 (0%)	0 (0%)	1	1	1	1	1 (8.33%)	0 (0%)	0 (0%)	0.25	1	1	1	0 (0%)	0 (0%)	0 (0%)	1	1	1	1
TSC2	5 (3.79%)	5 (1.89%)	0 (0%)	0.3116	1	0.0603	1	0 (0%)	0 (0%)	0 (0%)	1	1	1	1	1 (8.33%)	0 (0%)	0 (0%)	0.25	1	1	1	0 (0%)	1 (5.56%)	0 (0%)	1	1	1	1
TSHR	1 (0.76%)	2 (0.76%)	0 (0%)	1	1	1	1	0 (0%)	0 (0%)	0 (0%)	1	1	1	1	0 (0%)	0 (0%)	0 (0%)	1	1	1	1	0 (0%)	0 (0%)	0 (0%)	1	1	1	1
U2AF1	1 (0.76%)	4 (1.52%)	0 (0%)	0.6687	1	1	1	0 (0%)	0 (0%)	0 (0%)	1	1	1	1	0 (0%)	0 (0%)	0 (0%)	1	1	1	1	0 (0%)	0 (0%)	0 (0%)	1	1	1	1
VHL	0 (0%)	1 (0.38%)	0 (0%)	1	1	1	1	0 (0%)	0 (0%)	0 (0%)	1	1	1	1	0 (0%)	0 (0%)	0 (0%)	1	1	1	1	0 (0%)	0 (0%)	0 (0%)	1	1	1	1
VTCN1	1 (0.76%)	3 (1.14%)	1 (0.79%)	1	1	1	1	0 (0%)	0 (0%)	0 (0%)	1	1	1	1	0 (0%)	0 (0%)	0 (0%)	1	1	1	1	0 (0%)	0 (0%)	0 (0%)	1	1	1	1
WT1	1 (0.76%)	6 (2.27%)	2 (1.57%)	0.4324	1	0.6164	1	0 (0%)	0 (0%)	0 (0%)	1	1	1	1	0 (0%)	0 (0%)	0 (0%)	1	1	1	1	0 (0%)	0 (0%)	0 (0%)	1	1	1	1
XIAP	1 (0.76%)	1 (0.38%)	1 (0.79%)	1	1	1	1	0 (0%)	0 (0%)	0 (0%)	1	1	1	1	0 (0%)	0 (0%)	0 (0%)	1	1	1	1	0 (0%)	0 (0%)	0 (0%)	1	1	1	1
XPO1	4 (3.03%)	3 (1.14%)	0 (0%)	0.2283	1	0.1224	1	0 (0%)	0 (0%)	0 (0%)	1	1	1	1	0 (0%)	0 (0%)	0 (0%)	1	1	1	1	0 (0%)	0 (0%)	0 (0%)	1	1	1	1
YAP1	1 (0.76%)	1 (0.38%)	0 (0%)	1	1	1	1	0 (0%)	0 (0%)	0 (0%)	1	1	1	1	0 (0%)	1 (2.78%)	0 (0%)	1	1	1	1	0 (0%)	1 (5.56%)	0 (0%)	1	1	1	1
YES1	1 (0.76%)	9 (3.41%)	0 (0%)	0.175	0.9625	1	1	0 (0%)	0 (0%)	0 (0%)	1	1	1	1	0 (0%)	1 (2.78%)	0 (0%)	1	1	1	1	0 (0%)	2 (11.11%)	0 (0%)	1	1	1	1

*Muliple correction testing using the Benjamini Hochberg procedure

Supplementary Table 4. Non synonymous somatic mutations in breast cancers of histologic special type included in this study identified by targeted massively parallel sequencing (MSK-IMPACT)

Sample ID	Histology	Sample type	Gene symbol	Aminoacid change	Chromosome	Start Position	End Position	Reference Allele	Tumor Seq Allele1	Tumor_Seq_Allele2	Type of mutation	HOTSPOT	Loss of heterozygosity
MSK-ILC-150	Invasive Lobular Carcinoma	Metastasis	ASXL2	p.F373L	2	25,976,426	25,976,426	G	G	C	Missense_Mutation	FALSE	FALSE
MSK-ILC-150	Invasive Lobular Carcinoma	Metastasis	NOTCH1	p.F357L	9	139,413,071	139,413,071	G	G	C	Missense_Mutation	FALSE	FALSE
MSK-ILC-150	Invasive Lobular Carcinoma	Metastasis	PTEN	p.K13*	10	89,624,263	89,624,263	A	A	T	Nonsense_Mutation	FALSE	FALSE
MSK-ILC-150	Invasive Lobular Carcinoma	Metastasis	CDH1	p.V252Efs*30	16	68,844,167	68,844,167	T	T	-	Frame_Shift_Del	FALSE	TRUE
MSK-ILC-150	Invasive Lobular Carcinoma	Metastasis	RB1	p.N867Sfs*2	13	49,050,914	49,050,918	CAACC	CAACC	-	Frame_Shift_Del	FALSE	TRUE
MSK-ILC-56	Invasive Lobular Carcinoma	Primary	BCOR	p.R1678H	X	39,911,597	39,911,597	C	C	T	Missense_Mutation	FALSE	FALSE
MSK-ILC-56	Invasive Lobular Carcinoma	Primary	FLT1	p.C1039Y	13	28,895,658	28,895,658	C	C	T	Missense_Mutation	FALSE	FALSE
MSK-ILC-56	Invasive Lobular Carcinoma	Primary	CDH1	p.T515_F516ins*	16	68,849,641	68,849,642	-	-	ATA	Nonsense_Mutation	FALSE	TRUE
MSK-ILC-56	Invasive Lobular Carcinoma	Primary	TBX3	p.Y271*	12	115,117,361	115,117,362	-	-	T	Nonsense_Mutation	FALSE	FALSE
MSK-ILC-104	Invasive Lobular Carcinoma	Metastasis	PIK3CA	p.H1047R	3	178,952,085	178,952,085	A	A	G	Missense_Mutation	TRUE	FALSE
MSK-ILC-104	Invasive Lobular Carcinoma	Metastasis	ARID1A	p.Q1584*	1	27,101,468	27,101,468	C	C	T	Nonsense_Mutation	FALSE	TRUE
MSK-ILC-104	Invasive Lobular Carcinoma	Metastasis	CDH1	p.E852Rfs*9	16	68,867,305	68,867,306	-	-	A	Frame_Shift_Ins	FALSE	TRUE
MSK-ILC-179	Invasive Lobular Carcinoma	Primary	PIK3CA	p.N345K	3	178,921,553	178,921,553	T	T	A	Missense_Mutation	TRUE	FALSE
MSK-ILC-179	Invasive Lobular Carcinoma	Primary	NCOR1	p.Y666*	17	16,021,255	16,021,259	TAAAA	TAAAA	-	Nonsense_Mutation	FALSE	FALSE
MSK-ILC-215	Invasive Lobular Carcinoma	Primary	CBFB	p.E13*	16	67,063,347	67,063,347	G	G	T	Nonsense_Mutation	FALSE	TRUE
MSK-ILC-215	Invasive Lobular Carcinoma	Primary	CDH1	p.X380_splice	16	68,847,167	68,847,249	TGCTAGCAGTCTTGGTACTTTGTAAATGACACATCTCTTTGCTCTGCAGTACAAGGGTCAGGTGCCTGAGAACGAGGCTAACG	TGCTAGCAGTCTTGGTACTTTGTAAATGACACATCTCTTTGCTCTGCAGTACAAGGGTCAGGTGCCTGAGAACGAGGCTAACG	-	Splice_Site	FALSE	TRUE
MSK-ILC-215	Invasive Lobular Carcinoma	Primary	KDM6A	p.K905*	X	44,935,950	44,935,951	-	-	T	Frame_Shift_Ins	FALSE	FALSE
MSK-ILC-37	Invasive Lobular Carcinoma	Metastasis	SDHB	p.E176G	1	17,354,257	17,354,257	T	T	C	Missense_Mutation	FALSE	FALSE
MSK-ILC-37	Invasive Lobular Carcinoma	Metastasis	PIK3CA	p.E545K	3	178,936,091	178,936,091	G	G	A	Missense_Mutation	TRUE	FALSE
MSK-ILC-37	Invasive Lobular Carcinoma	Metastasis	KDR	p.E755Q	4	55,968,067	55,968,067	C	C	G	Missense_Mutation	FALSE	FALSE
MSK-ILC-37	Invasive Lobular Carcinoma	Metastasis	INPP4B	p.I314V	4	143,130,076	143,130,076	T	T	C	Missense_Mutation	FALSE	FALSE
MSK-ILC-37	Invasive Lobular Carcinoma	Metastasis	FAT1	p.R881C	4	187,628,341	187,628,341	G	G	A	Missense_Mutation	FALSE	FALSE
MSK-ILC-37	Invasive Lobular Carcinoma	Metastasis	ARID1B	p.A767T	6	157,431,662	157,431,662	G	G	A	Missense_Mutation	FALSE	FALSE
MSK-ILC-37	Invasive Lobular Carcinoma	Metastasis	PTCH1	p.E604K	9	98,232,132	98,232,132	C	C	T	Missense_Mutation	FALSE	FALSE
MSK-ILC-37	Invasive Lobular Carcinoma	Metastasis	GATA3	p.R9C	10	8,097,643	8,097,643	C	C	T	Missense_Mutation	FALSE	FALSE
MSK-ILC-37	Invasive Lobular Carcinoma	Metastasis	RAD52	p.R396C	12	1,023,069	1,023,069	G	G	A	Missense_Mutation	FALSE	FALSE
MSK-ILC-37	Invasive Lobular Carcinoma	Metastasis	BLM	p.D64V	15	91,292,689	91,292,689	A	A	T	Missense_Mutation	FALSE	FALSE
MSK-ILC-37	Invasive Lobular Carcinoma	Metastasis	CDH1	p.Y523C	16	68,853,185	68,853,185	A	A	G	Missense_Mutation	FALSE	FALSE
MSK-ILC-37	Invasive Lobular Carcinoma	Metastasis	TP53	p.Q192*	17	7,578,275	7,578,275	G	G	A	Nonsense_Mutation	TRUE	FALSE
MSK-ILC-37	Invasive Lobular Carcinoma	Metastasis	STK11	p.F354L	19	1,223,125	1,223,125	C	C	G	Missense_Mutation	FALSE	FALSE
MSK-ILC-37	Invasive Lobular Carcinoma	Metastasis	MAP2K2	p.R92G	19	4,117,446	4,117,446	T	T	C	Missense_Mutation	FALSE	FALSE
MSK-ILC-37	Invasive Lobular Carcinoma	Metastasis	EP300	p.S535L	22	41,531,892	41,531,892	C	C	T	Missense_Mutation	FALSE	FALSE
MSK-ILC-70	Invasive Lobular Carcinoma	Primary	ERBB3	p.E928G	12	56,492,633	56,492,633	A	A	G	Missense_Mutation	TRUE	FALSE
MSK-ILC-70	Invasive Lobular Carcinoma	Primary	EPHB1	p.L725I	3	134,920,358	134,920,358	C	C	A	Missense_Mutation	FALSE	FALSE
MSK-ILC-70	Invasive Lobular Carcinoma	Primary	CDH1	p.D644Tfs*9	16	68,856,122	68,856,122	G	G	-	Frame_Shift_Del	FALSE	TRUE
MSK-ILC-84	Invasive Lobular Carcinoma	Metastasis	ESR1	p.D538G	6	152,419,926	152,419,926	A	A	G	Missense_Mutation	TRUE	FALSE
MSK-ILC-171	Invasive Lobular Carcinoma	Primary	BRCA2	p.R2034C	13	32,914,592	32,914,592	C	C	T	Missense_Mutation	FALSE	FALSE
MSK-ILC-171	Invasive Lobular Carcinoma	Primary	CDH1	p.D349_A359del	16	68,846,071	68,846,103	GCTGACCTTCAAGGTGAGGGGTTAAGCACAACA	GCTGACCTTCAAGGTGAGGGGTTAAGCACAACA	-	In_Frame_Del	FALSE	FALSE
MSK-ILC-171	Invasive Lobular Carcinoma	Primary	DNMT3B	p.L489F	20	31,385,080	31,385,080	C	C	T	Missense_Mutation	FALSE	FALSE
MSK-ILC-49	Invasive Lobular Carcinoma	Metastasis	ESR1	p.D538G	6	152,419,926	152,419,926	A	A	G	Missense_Mutation	TRUE	FALSE
[illegible]	[illegible]	[illegible]	[illegible]	[illegible]	[illegible]	[illegible]	[illegible]	[illegible]	[illegible]	[illegible]	[illegible]	[illegible]	[illegible]

MSK-ILC-18	Invasive Lobular Carcinoma	Metastasis	RNF43	p.R219C	17	56,439,937	56,439,937	G	G	A	Missense_Mutation	FALSE	FALSE
MSK-ILC-18	Invasive Lobular Carcinoma	Metastasis	CDH1	p.Y827*	16	68,867,232	68,867,233	TA	TA	-	Frame_Shift_Del	FALSE	FALSE
MSK-ILC-68	Invasive Lobular Carcinoma	Metastasis	ERBB2	p.L755S	17	37,880,220	37,880,220	T	T	C	Missense_Mutation	TRUE	FALSE
MSK-ILC-68	Invasive Lobular Carcinoma	Metastasis	RUNX1	p.D96Gfs*15	21	36,252,995	36,252,996	-	-	C	Frame_Shift_Ins	FALSE	FALSE
MSK-ILC-123	Invasive Lobular Carcinoma	Metastasis	PTEN	p.L23V	10	89,624,293	89,624,293	T	T	G	Missense_Mutation	FALSE	FALSE
MSK-ILC-123	Invasive Lobular Carcinoma	Metastasis	PTEN	p.C124S	10	89,692,887	89,692,887	G	G	C	Missense_Mutation	TRUE	FALSE
MSK-ILC-123	Invasive Lobular Carcinoma	Metastasis	FOXA1	p.S250F	14	38,061,240	38,061,240	G	G	A	Missense_Mutation	TRUE	FALSE
MSK-ILC-123	Invasive Lobular Carcinoma	Metastasis	CDH1	p.E310*	16	68,845,682	68,845,682	G	G	T	Nonsense_Mutation	FALSE	TRUE
MSK-ILC-143	Invasive Lobular Carcinoma	Metastasis	DDR2	p.P840S	1	162,749,986	162,749,986	C	C	T	Missense_Mutation	FALSE	FALSE
MSK-ILC-143	Invasive Lobular Carcinoma	Metastasis	PTEN	p.R130P	10	89,692,905	89,692,905	G	G	C	Missense_Mutation	TRUE	FALSE
MSK-ILC-143	Invasive Lobular Carcinoma	Metastasis	TP53	p.E180*	17	7,578,392	7,578,392	C	C	A	Nonsense_Mutation	FALSE	TRUE
MSK-ILC-143	Invasive Lobular Carcinoma	Metastasis	EPHB1	p.S324L	3	134,851,565	134,851,565	C	C	T	Missense_Mutation	FALSE	TRUE
MSK-ILC-143	Invasive Lobular Carcinoma	Metastasis	FAT1	p.S3526L	4	187,525,103	187,525,103	G	G	A	Missense_Mutation	FALSE	FALSE
MSK-ILC-143	Invasive Lobular Carcinoma	Metastasis	FAT1	p.E1925V	4	187,541,986	187,541,986	T	T	A	Missense_Mutation	FALSE	FALSE
MSK-ILC-143	Invasive Lobular Carcinoma	Metastasis	PIK3CG	p.I831M	7	106,520,065	106,520,065	C	C	G	Missense_Mutation	FALSE	FALSE
MSK-ILC-143	Invasive Lobular Carcinoma	Metastasis	ABL1	p.W261*	9	133,738,383	133,738,383	G	G	A	Nonsense_Mutation	FALSE	FALSE
MSK-ILC-143	Invasive Lobular Carcinoma	Metastasis	KDM5A	p.E771K	12	431,698	431,698	C	C	T	Missense_Mutation	FALSE	FALSE
MSK-ILC-143	Invasive Lobular Carcinoma	Metastasis	FLT3	p.E360*	13	28,622,539	28,622,539	C	C	A	Nonsense_Mutation	FALSE	FALSE
MSK-ILC-143	Invasive Lobular Carcinoma	Metastasis	DIS3	p.E117Q	13	73,355,021	73,355,021	C	C	G	Missense_Mutation	FALSE	TRUE
MSK-ILC-143	Invasive Lobular Carcinoma	Metastasis	FOXA1	p.F396L	14	38,060,801	38,060,801	G	G	T	Missense_Mutation	FALSE	TRUE
MSK-ILC-143	Invasive Lobular Carcinoma	Metastasis	CDH1	p.W103*	16	68,835,718	68,835,718	G	G	A	Nonsense_Mutation	FALSE	TRUE
MSK-ILC-143	Invasive Lobular Carcinoma	Metastasis	MAP2K4	p.R228T	17	12,013,741	12,013,741	G	G	C	Missense_Mutation	FALSE	TRUE
MSK-ILC-143	Invasive Lobular Carcinoma	Metastasis	CDK12	p.D1100N	17	37,681,129	37,681,129	G	G	A	Missense_Mutation	FALSE	TRUE
MSK-ILC-143	Invasive Lobular Carcinoma	Metastasis	PTPRS	p.D1144N	19	5,221,036	5,221,036	C	C	T	Missense_Mutation	FALSE	TRUE
MSK-ILC-143	Invasive Lobular Carcinoma	Metastasis	JAK3	p.D661N	19	17,945,958	17,945,958	C	C	T	Missense_Mutation	FALSE	FALSE
MSK-ILC-143	Invasive Lobular Carcinoma	Metastasis	AMER1	p.E480K	X	63,411,729	63,411,729	C	C	T	Missense_Mutation	FALSE	TRUE
MSK-ILC-143	Invasive Lobular Carcinoma	Metastasis	ATRX	p.D1051N	X	76,937,597	76,937,597	C	C	T	Missense_Mutation	FALSE	TRUE
MSK-ILC-143	Invasive Lobular Carcinoma	Metastasis	TBX3	p.N211Qfs*16	12	115,118,711	115,118,712	-	-	G	Frame_Shift_Ins	FALSE	FALSE
MSK-ILC-146	Invasive Lobular Carcinoma	Metastasis	PIK3CA	p.H1047R	3	178,952,085	178,952,085	A	A	G	Missense_Mutation	TRUE	FALSE
MSK-ILC-146	Invasive Lobular Carcinoma	Metastasis	TP53	p.E180K	17	7,578,392	7,578,392	C	C	T	Missense_Mutation	FALSE	FALSE
MSK-ILC-146	Invasive Lobular Carcinoma	Metastasis	TBX3	p.P48Lfs*56	12	115,120,844	115,120,863	GAGCGCCGCCGCGCCGTTGG	GAGCGCCGCCGCGCCGTTGG	-	Frame_Shift_Del	FALSE	FALSE
MSK-ILC-93	Invasive Lobular Carcinoma	Primary	PIK3CA	p.H1047R	3	178,952,085	178,952,085	A	A	G	Missense_Mutation	TRUE	FALSE
MSK-ILC-93	Invasive Lobular Carcinoma	Primary	ATR	p.S272L	3	142,281,429	142,281,429	G	G	A	Missense_Mutation	FALSE	FALSE
MSK-ILC-93	Invasive Lobular Carcinoma	Primary	CDH1	p.W20*	16	68,772,210	68,772,210	G	G	A	Nonsense_Mutation	FALSE	FALSE
MSK-ILC-93	Invasive Lobular Carcinoma	Primary	FOXA1	p.K267_C268insR	14	38,061,188	38,061,189	-	-	CTT	In_Frame_Ins	FALSE	FALSE
MSK-ILC-93	Invasive Lobular Carcinoma	Primary	RUNX1	p.M341Hfs*232	21	36,164,775	36,164,776	-	-	C	Frame_Shift_Ins	FALSE	FALSE
MSK-ILC-47	Invasive Lobular Carcinoma	Metastasis	PARP1	p.E235Q	1	226,576,371	226,576,371	C	C	G	Missense_Mutation	FALSE	FALSE
MSK-ILC-47	Invasive Lobular Carcinoma	Metastasis	MSH2	p.S269*	2	47,641,421	47,641,421	C	C	G	Nonsense_Mutation	FALSE	FALSE
MSK-ILC-47	Invasive Lobular Carcinoma	Metastasis	RASA1	p.F690C	5	86,672,267	86,672,267	T	T	G	Missense_Mutation	FALSE	FALSE
MSK-ILC-47	Invasive Lobular Carcinoma	Metastasis	ESR1	p.N532K	6	152,419,909	152,419,909	C	C	G	Missense_Mutation	FALSE	FALSE
MSK-ILC-47	Invasive Lobular Carcinoma	Metastasis	ERBB3	p.D501N	12	56,487,568	56,487,568	G	G	A	Missense_Mutation	FALSE	FALSE
MSK-ILC-47	Invasive Lobular Carcinoma	Metastasis	TBX3	p.S554W	12	115,112,079	115,112,079	G	G	C	Missense_Mutation	FALSE	FALSE
MSK-ILC-47	Invasive Lobular Carcinoma	Metastasis	TBX3	p.N211T	12	115,118,709	115,118,709	T	T	G	Missense_Mutation	FALSE	FALSE
MSK-ILC-47	Invasive Lobular Carcinoma	Metastasis	CDH1	p.X278_splice	16	68,845,585	68,845,585	A	A	G	Splice_Site	TRUE	TRUE
MSK-ILC-47	Invasive Lobular Carcinoma	Metastasis	PTEN	p.I33del	10	89,653,796	89,653,798	ATT	ATT	-	In_Frame_Del	FALSE	FALSE
MSK-ILC-47	Invasive Lobular Carcinoma	Metastasis	TBX3	p.D66Ifs*22	12	115,120,810	115,120,810	C	C	-	Frame_Shift_Del	FALSE	FALSE
MSK-ILC-32	Invasive Lobular Carcinoma	Metastasis	PIK3CA	p.E81K	3	178,916,854	178,916,854	G	G	A	Missense_Mutation	TRUE	FALSE
MSK-ILC-32	Invasive Lobular Carcinoma	Metastasis	RB1	p.X406_splice	13	48,951,053	48,951,053	G	G	T	Splice_Site	TRUE	TRUE
MSK-ILC-32	Invasive Lobular Carcinoma	Metastasis	CDH1	p.V204Lfs*4	16	68,842,672	68,842,673	GT	GT	-	Frame_Shift_Del	FALSE	TRUE
MSK-ILC-90	Invasive Lobular Carcinoma	Primary	NCOR1	p.Q59*	17	16,089,935	16,089,935	G	G	A	Nonsense_Mutation	FALSE	TRUE
MSK-ILC-54	Invasive Lobular Carcinoma	Metastasis	PIK3CA	p.H1047R	3	178,952,085	178,952,085	A	A	G	Missense_Mutation	TRUE	FALSE
MSK-ILC-54	Invasive Lobular Carcinoma	Metastasis	PIK3CD	p.E197Q	1	9,776,125	9,776,125	G	G	C	Missense_Mutation	FALSE	FALSE
MSK-ILC-54	Invasive Lobular Carcinoma	Metastasis	BRCA2	p.R118C	13	32,899,248	32,899,248	C	C	T	Missense_Mutation	FALSE	TRUE
MSK-ILC-116	Invasive Lobular Carcinoma	Metastasis	PIK3CA	p.R93W	3	178,916,890	178,916,890	C	C	T	Missense_Mutation	TRUE	FALSE
MSK-ILC-6	Invasive Lobular Carcinoma	Metastasis	FLT4	p.A727V	5	180,047,995	180,047,995	G	G	A	Missense_Mutation	FALSE	FALSE
MSK-ILC-6	Invasive Lobular Carcinoma	Metastasis	ESR1	p.E380Q	6	152,332,832	152,332,832	G	G	C	Missense_Mutation	TRUE	FALSE
MSK-ILC-6	Invasive Lobular Carcinoma	Metastasis	CDH1	p.X278_splice	16	68,844,246	68,844,246	T	T	C	Splice_Site	TRUE	FALSE
[illegible]													

Supplementary Table 4
Page 3

MSK-ILC-108	Invasive Lobular Carcinoma	Metastasis	TP53	p.C238Y	17	7,577,568	7,577,568
MSK-ILC-108	Invasive Lobular Carcinoma	Metastasis	RHOA	p.G17E	3	49,412,973	49,412,973
MSK-ILC-108	Invasive Lobular Carcinoma	Metastasis	CDH1	p.X765_splice	16	68,862,208	68,862,208
MSK-ILC-108	Invasive Lobular Carcinoma	Metastasis	ATM	p.E574K	11	108,122,676	108,122,676
MSK-ILC-108	Invasive Lobular Carcinoma	Metastasis	TBX3	p.S547Vfs*85	12	115,112,100	115,112,101
MSK-ILC-186	Invasive Lobular Carcinoma	Primary	CDH1	p.P200Tfs*16	16	68,842,660	68,842,661
MSK-ILC-58	Invasive Lobular Carcinoma	Primary	PIK3CA	p.H1047R	3	178,952,085	178,952,085
MSK-ILC-58	Invasive Lobular Carcinoma	Primary	SMAD4	p.Q245*	18	48,584,560	48,584,560
MSK-ILC-58	Invasive Lobular Carcinoma	Primary	PIK3CA	p.R88Q	3	178,916,876	178,916,876
MSK-ILC-58	Invasive Lobular Carcinoma	Primary	CDKN1B	p.A98Cfs*27	12	12,871,064	12,871,065
MSK-ILC-58	Invasive Lobular Carcinoma	Primary	FAM175A	p.A382G	4	84,383,707	84,383,707
MSK-ILC-58	Invasive Lobular Carcinoma	Primary	CD276	p.S525R	15	74,003,504	74,003,504
MSK-ILC-58	Invasive Lobular Carcinoma	Primary	CTCF	p.Y523D	16	67,662,321	67,662,321
MSK-ILC-58	Invasive Lobular Carcinoma	Primary	FAM175A	p.M385*	4	84,383,703	84,383,703
MSK-ILC-100	Invasive Lobular Carcinoma	Metastasis	CDH1	p.A634V	16	68,856,093	68,856,093
MSK-ILC-100	Invasive Lobular Carcinoma	Metastasis	FAT1	p.S1803*	4	187,542,332	187,542,332
MSK-ILC-100	Invasive Lobular Carcinoma	Metastasis	TP53	p.S149Ffs*32	17	7,578,485	7,578,486
MSK-ILC-100	Invasive Lobular Carcinoma	Metastasis	CREBBP	p.H1485Y	16	3,786,758	3,786,758
MSK-ILC-100	Invasive Lobular Carcinoma	Metastasis	LATS1	p.E534K	6	150,004,625	150,004,625
MSK-ILC-100	Invasive Lobular Carcinoma	Metastasis	MYD88	p.I130M	3	38,181,377	38,181,377
MSK-ILC-100	Invasive Lobular Carcinoma	Metastasis	RHOA	p.P69R	3	49,405,872	49,405,872
MSK-ILC-100	Invasive Lobular Carcinoma	Metastasis	PDGFRA	p.P864S	4	55,153,624	55,153,624
MSK-ILC-100	Invasive Lobular Carcinoma	Metastasis	RICTOR	p.E410Q	5	38,966,814	38,966,814
MSK-ILC-100	Invasive Lobular Carcinoma	Metastasis	NOTCH4	p.G796C	6	32,180,964	32,180,964
MSK-ILC-100	Invasive Lobular Carcinoma	Metastasis	ARID5B	p.E765Q	10	63,851,515	63,851,515
MSK-ILC-100	Invasive Lobular Carcinoma	Metastasis	ETV6	p.S16L	12	11,905,397	11,905,397
MSK-ILC-100	Invasive Lobular Carcinoma	Metastasis	IDH2	p.X39_splice	15	90,634,877	90,634,877
MSK-ILC-100	Invasive Lobular Carcinoma	Metastasis	SMARCA4	p.R1618Q	19	11,170,805	11,170,805
MSK-ILC-185	Invasive Lobular Carcinoma	Metastasis	PTPRS	p.P1024S	19	5,222,733	5,222,733
MSK-ILC-185	Invasive Lobular Carcinoma	Metastasis	TSC2	p.E234K	16	2,106,696	2,106,696
MSK-ILC-185	Invasive Lobular Carcinoma	Metastasis	PTEN	p.R130Q	10	89,692,905	89,692,905
MSK-ILC-185	Invasive Lobular Carcinoma	Metastasis	MPL	p.R445Q	1	43,814,539	43,814,539
MSK-ILC-185	Invasive Lobular Carcinoma	Metastasis	FANCA	p.E84K	16	89,880,961	89,880,961
MSK-ILC-185	Invasive Lobular Carcinoma	Metastasis	PIK3R1	p.F456_Q457del	5	67,589,599	67,589,604
MSK-ILC-185	Invasive Lobular Carcinoma	Metastasis	SDHC	p.T57I	1	161,298,278	161,298,278
MSK-ILC-185	Invasive Lobular Carcinoma	Metastasis	PARP1	p.E76*	1	226,589,975	226,589,975
MSK-ILC-185	Invasive Lobular Carcinoma	Metastasis	NFE2L2	p.L200V	2	178,096,733	178,096,733
MSK-ILC-185	Invasive Lobular Carcinoma	Metastasis	IL7R	p.Q26E	5	35,857,155	35,857,155
MSK-ILC-185	Invasive Lobular Carcinoma	Metastasis	MAP3K1	p.Q1259E	5	56,179,462	56,179,462
MSK-ILC-185	Invasive Lobular Carcinoma	Metastasis	APC	p.H1845N	5	112,176,824	112,176,824
MSK-ILC-185	Invasive Lobular Carcinoma	Metastasis	PDGFRB	p.E472K	5	149,509,485	149,509,485
MSK-ILC-185	Invasive Lobular Carcinoma	Metastasis	NSD1	p.R2558T	5	176,722,042	176,722,042
MSK-ILC-185	Invasive Lobular Carcinoma	Metastasis	E2F3	p.S172C	6	20,481,446	20,481,446
MSK-ILC-185	Invasive Lobular Carcinoma	Metastasis	ARID1B	p.S361W	6	157,100,145	157,100,145
MSK-ILC-185	Invasive Lobular Carcinoma	Metastasis	PIK3C2G	p.S1291L	12	18,719,975	18,719,975
MSK-ILC-185	Invasive Lobular Carcinoma	Metastasis	CDH1	p.S9*	16	68,771,344	68,771,344
MSK-ILC-185	Invasive Lobular Carcinoma	Metastasis	AURKB	p.M244I	17	8,108,663	8,108,663
MSK-ILC-185	Invasive Lobular Carcinoma	Metastasis	NF1	p.G751*	17	29,553,702	29,553,702
MSK-ILC-185	Invasive Lobular Carcinoma	Metastasis	DOT1L	p.S225*	19	2,199,905	2,199,905
MSK-ILC-185	Invasive Lobular Carcinoma	Metastasis	MED12	p.S700F	X	70,344,869	70,344,869
MSK-ILC-173	Invasive Lobular Carcinoma	Primary	PIK3CA	p.E545K	3	178,936,091	178,936,091
MSK-ILC-173	Invasive Lobular Carcinoma	Primary	EGFR	p.T1041A	7	55,269,434	55,269,434
MSK-ILC-173	Invasive Lobular Carcinoma	Primary	CBL	p.Q175*	11	119,142,524	119,142,524
MSK-ILC-173	Invasive Lobular Carcinoma	Primary	TBX3	p.S396Pfs*236	12	115,112,555	115,112,555
MSK-ILC-173	Invasive Lobular Carcinoma	Primary	CDH1	p.F516Nfs*4	16	68,849,643	68,849,649
MSK-ILC-156	Invasive Lobular Carcinoma	Primary	PIK3CA	p.H1047R	3	178,952,085	178,952,085
MSK-ILC-156	Invasive Lobular Carcinoma	Primary	DNMT1	p.Q181E	19	10,286,275	10,286,275
MSK-ILC-156	Invasive Lobular Carcinoma	Primary	MAP3K1	p.P1228Tfs*11	5	56,179,367	56,179,368
MSK-ILC-156	Invasive Lobular Carcinoma	Primary	CDH1	p.Y190Lfs*9	16	68,842,632	68,842,633
MSK-ILC-190	Invasive Lobular Carcinoma	Primary	KRAS	p.G12A	12	25,398,284	25,398,284
MSK-ILC-190	Invasive Lobular Carcinoma	Primary	PIK3CA	p.C420R	3	178,927,980	178,927,980
MSK-ILC-190	Invasive Lobular Carcinoma	Primary	PTEN	p.R234Pfs*9	10	89,717,675	89,717,676
MSK-ILC-190	Invasive Lobular Carcinoma	Primary	BRAF	p.G464V	7	140,481,417	140,481,417
MSK-ILC-190	Invasive Lobular Carcinoma	Primary	ASXL1	p.R1073C	20	31,023,732	31,023,732
MSK-ILC-190	Invasive Lobular Carcinoma	Primary	PIK3R3	p.L421P	1	46,509,469	46,509,469
MSK-ILC-190	Invasive Lobular Carcinoma	Primary	FLT4	p.R378L	5	180,053,236	180,053,236
MSK-ILC-190	Invasive Lobular Carcinoma	Primary	PTEN	p.E114Dfs*17	10	89,692,858	89,692,867
MSK-ILC-151	Invasive Lobular Carcinoma	Primary	PIK3CA	p.E542K	3	178,936,082	178,936,082
MSK-ILC-151	Invasive Lobular Carcinoma	Primary	PIK3CA	p.E726K	3	178,938,934	178,938,934
MSK-ILC-151	Invasive Lobular Carcinoma	Primary	NPM1	p.Q153H	5	170,819,820	170,819,820
MSK-ILC-151	Invasive Lobular Carcinoma	Primary	ESR1	p.L466Q	6	152,415,547	152,415,547
MSK-ILC-151	Invasive Lobular Carcinoma	Primary	CDH1	p.P620Lfs*11	16	68,856,050	68,856,050
MSK-ILC-195	Invasive Lobular Carcinoma	Primary	TP53	p.X187_splice	17	7,578,370	7,578,370
MSK-ILC-195	Invasive Lobular Carcinoma	Primary	CDH1	p.Q23*	16	68,772,218	68,772,218
MSK-ILC-195	Invasive Lobular Carcinoma	Primary	FOXA1	p.G322Rfs*10	14	38,060,971	38,061,025
MSK-ILC-195	Invasive Lobular Carcinoma	Primary	NCOR1	p.V706Kfs*49	17	16,012,128	16,012,167
MSK-ILC-195	Invasive Lobular Carcinoma	Primary	NCOR1	p.E526Kfs*9	17	16,029,453	16,029,454
MSK-ILC-11	Invasive Lobular Carcinoma	Metastasis	ESR1	p.Y537S	6	152,419,923	152,419,923
MSK-ILC-11	Invasive Lobular Carcinoma	Metastasis	PIK3CA	p.Q546R	3	178,936,095	178,936,095
MSK-ILC-165	Invasive Lobular Carcinoma	Metastasis	PIK3CA	p.H1047R	3	178,952,085	178,952,085
MSK-ILC-165	Invasive Lobular Carcinoma	Metastasis	PIK3CA	p.E81K	3	178,916,854	178,916,854
MSK-ILC-165	Invasive Lobular Carcinoma	Metastasis	SMAD4	p.E330K	18	48,591,825	48,591,825
MSK-ILC-165	Invasive Lobular Carcinoma	Metastasis	SPOP	p.E78K	17	47,696,716	47,696,716
MSK-ILC-165	Invasive Lobular Carcinoma	Metastasis	MCL1	p.E284Q	1	150,550,806	150,550,806
MSK-ILC-165	Invasive Lobular Carcinoma	Metastasis	FGFR4	p.E227Q	5	176,518,761	176,518,761
MSK-ILC-165	Invasive Lobular Carcinoma	Metastasis	PNRC1	p.H196D	6	89,793,517	89,793,517
MSK-ILC-165	Invasive Lobular Carcinoma	Metastasis	BLM	p.S147F	15	91,292,938	91,292,938
MSK-ILC-165	Invasive Lobular Carcinoma	Metastasis	CDH1	p.N751Kfs*19	16	68,862,165	68,862,165
MSK-ILC-184	Invasive Lobular Carcinoma	Primary	PIK3CA	p.E545K	3	178,936,091	178,936,091
MSK-ILC-184	Invasive Lobular Carcinoma	Primary	IKBKE	p.R236W	1	206,651,096	206,651,096
MSK-ILC-184	Invasive Lobular Carcinoma	Primary	CDH1	p.E410Gfs*9	16	68,847,304	68,847,305
MSK-ILC-62	Invasive Lobular Carcinoma	Metastasis	PIK3CA	p.E542K	3	178,936,082	178,936,082
MSK-ILC-72	Invasive Lobular Carcinoma	Metastasis	PIK3CA	p.H1047R	3	178,952,085	178,952,085
MSK-ILC-72	Invasive Lobular Carcinoma	Metastasis	CDH1	p.Q610*	16	68,856,020	68,856,020
MSK-ILC-72	Invasive Lobular Carcinoma	Metastasis	NTRK1	p.R761Q	1	156,851,325	156,851,325
MSK-ILC-72	Invasive Lobular Carcinoma	Metastasis	RICTOR	p.G1511E	5	38,945,694	38,945,694
MSK-ILC-158	Invasive Lobular Carcinoma	Primary	PTEN	p.Q171R	10	89,711,894	89,711,894
MSK-ILC-158	Invasive Lobular Carcinoma	Primary	NTRK2	p.H343R	9	87,342,743	87,342,743
MSK-ILC-158	Invasive Lobular Carcinoma	Primary	CDH1	p.T287_A289del	16	68,845,610	68,845,618
MSK-ILC-158	Invasive Lobular Carcinoma	Primary	PTEN	p.T321Qfs*23	10	89,720,809	89,720,809
MSK-ILC-19	Invasive Lobular Carcinoma	Metastasis	ESR1	p.D538G	6	152,419,926	152,419,926
MSK-ILC-19	Invasive Lobular Carcinoma	Metastasis	AR	p.L57del	X	66,765,149	66,765,151
MSK-ILC-19	Invasive Lobular Carcinoma	Metastasis	TNFAIP3	p.S459L	6	138,199,958	138,199,958
MSK-ILC-19	Invasive Lobular Carcinoma	Metastasis	FGFR4	p.V550M	5	176,522,551	176,522,551
MSK-ILC-19	Invasive Lobular Carcinoma	Metastasis	MTOR	p.A2210P	1	11,184,589	11,184,589
MSK-ILC-19	Invasive Lobular Carcinoma	Metastasis	RARA	p.L220V	17	38,508,610	38,508,610
MSK-ILC-19	Invasive Lobular Carcinoma	Metastasis	AKT1	p.R76_C77insSGPGPTPSSSR	14	105,243,058	105,243,059
MSK-ILC-19	Invasive Lobular Carcinoma	Metastasis	LATS2	p.R862Q	13	21,555,684	21,555,685
MSK-ILC-162	Invasive Lobular Carcinoma	Metastasis	CDH1	p.H453Rfs*10	16	68,849,453	68,849,454
MSK-ILC-160	Invasive Lobular Carcinoma	Primary	SMAD4	p.G386D	18	48,593,406	48,593,406
MSK-ILC-160	Invasive Lobular Carcinoma	Primary	SPEN	p.E1964*	1	16,258,625	16,258,625
MSK-ILC-160	Invasive Lobular Carcinoma	Primary	NOTCH2	p.Q1656P	1	120,465,294	120,465,294
MSK-ILC-160	Invasive Lobular Carcinoma	Primary	CDH1	p.Q641*	16	68,856,113	68,856,113
MSK-ILC-183	Invasive Lobular Carcinoma	Metastasis	ERBB2	p.L755S	17	37,880,220	37,880,220
MSK-ILC-183	Invasive Lobular Carcinoma	Metastasis	ERBB3	p.E928G	12	56,492,633	56,492,633
MSK-ILC-183	Invasive Lobular Carcinoma	Metastasis	POLE	p.P1692H	12	133,218,861	133,218,861
MSK-ILC-183	Invasive Lobular Carcinoma	Metastasis	CDH1	p.X178_splice	16	68,842,595	68,842,595
MSK-ILC-78	Invasive Lobular Carcinoma	Primary	TP53	p.E285K	17	7,577,085	7,577,085
MSK-ILC-78	Invasive Lobular Carcinoma	Primary	ERBB2	p.V777L	17	37,881,000	37,881,000
MSK-ILC-78	Invasive Lobular Carcinoma	Primary	DNMT3B	p.P159L	20	31,375,079	31,375,079
MSK-ILC-78	Invasive Lobular Carcinoma	Primary	CDH1	p.Q677Pfs*3	16	68,857,394	68,857,395
MSK-ILC-81	Invasive Lobular Carcinoma	Primary	MED12	p.L36R	X	70,339,230	70,339,230
MSK-ILC-81	Invasive Lobular Carcinoma	Primary	RARA	p.D288G	17	38,510,609	38,510,609
MSK-ILC-94	Invasive Lobular Carcinoma	Metastasis	PIK3CA	p.E545K	3	178,936,091	178,936,091
MSK-ILC-94	Invasive Lobular Carcinoma	Metastasis	ESR1	p.D538G	6	152,419,926	152,419,926
MSK-ILC-94	Invasive Lobular Carcinoma	Metastasis	ATM	p.E2164Q	11	108,192,065	108,192,065
MSK-ILC-94	Invasive Lobular Carcinoma	Metastasis	SETD2	p.Q1797*	3	47,127,693	47,127,693
MSK-ILC-94	Invasive Lobular Carcinoma	Metastasis	MTOR	p.R112W	1	11,317,160	11,317,160
MSK-ILC-94	Invasive Lobular Carcinoma	Metastasis	SPEN	p.E1400*	1	16,256,933	16,256,933
MSK-ILC-94	Invasive Lobular Carcinoma	Metastasis	SPEN	p.E1519K	1	16,257,290	16,257,290
MSK-ILC-94	Invasive Lobular Carcinoma	Metastasis	NOTCH2	p.F1304L	1	120,469,217	120,469,217
MSK-ILC-94	Invasive Lobular Carcinoma	Metastasis	ERBB4	p.A1097V	2	212,251,769	212,251,769
MSK-ILC-94	Invasive Lobular Carcinoma	Metastasis	RAF1	p.L613V	3	12,626,123	12,626,123
MSK-ILC-94	Invasive Lobular Carcinoma	Metastasis	RAF1	p.F443L	3	12,632,338	12,632,338
MSK-ILC-94	Invasive Lobular Carcinoma	Metastasis	SETD2	p.P2202T	3	47,098,670	47,098,670
MSK-ILC-94	Invasive Lobular Carcinoma	Metastasis	PBRM1	p.S383L	3	52,668,771	52,668,771
MSK-ILC-94	Invasive Lobular Carcinoma	Metastasis	MITF	p.R217Q	3	69,988,316	69,988,316
MSK-ILC-94	Invasive Lobular Carcinoma	Metastasis	FOXP1	p.X511_splice	3	71,021,828	71,021,828
MSK-ILC-94	Invasive Lobular Carcinoma	Metastasis	ATR	p.E2626K	3	142,168,330	142,168,330
MSK-ILC-94	Invasive Lobular Carcinoma	Metastasis	TP63	p.S622I	3	189,612,113	189,612,113
MSK-ILC-94	Invasive Lobular Carcinoma	Metastasis	FBXW7	p.Q306K	4	153,253,817	153,253,817
MSK-ILC-94	Invasive Lobular Carcinoma	Metastasis	FAT1	p.E2010K	4	187,541,712	187,541,712
MSK-ILC-94	Invasive Lobular Carcinoma	Metastasis	CSF1R	p.X361_splice	5	149,450,135	149,450,135
MSK-ILC-94	Invasive Lobular Carcinoma	Metastasis	PDGFRB	p.D1018Y	5	149,497,266	149,497,266
MSK-ILC-94	Invasive Lobular Carcinoma	Metastasis	FGFR4	p.N535K	5	176,522,416	176,522,416
MSK-ILC-94	Invasive Lobular Carcinoma	Metastasis	HIST1H3B	p.Q86H	6	26,032,031	26,032,031
MSK-ILC-94	Invasive Lobular Carcinoma	Metastasis	HIST1H1C	p.K212N	6	26,056,021	26,056,021
MSK-ILC-94	Invasive Lobular Carcinoma	Metastasis	NOTCH4	p.E550K	6	32,185,020	32,185,020
MSK-ILC-94	Invasive Lobular Carcinoma	Metastasis	LATS1	p.E450K	6	150,004,877	150,004,877
MSK-ILC-94	Invasive Lobular Carcinoma	Metastasis	NBN	p.X745_splice	8	90,947,841	90,947,841
MSK-ILC-94	Invasive Lobular Carcinoma	Metastasis	RECQL4	p.E606K	8	145,739,635	145,739,635
MSK-ILC-94	Invasive Lobular Carcinoma	Metastasis	FLT1	p.S1125C	13	28,891,647	28,891,647
MSK-ILC-94	Invasive Lobular Carcinoma	Metastasis	BRCA1	p.Q81E	17	41,256,945	41,256,945
MSK-ILC-94	Invasive Lobular Carcinoma	Metastasis	SPOP	p.E78K	17	47,696,716	47,696,716
MSK-ILC-94	Invasive Lobular Carcinoma	Metastasis	INSR	p.P1299L	19	7,117,320	7,117,320
MSK-ILC-94	Invasive Lobular Carcinoma	Metastasis	ERCC2	p.I71M	19	45,872,221	45,872,221
MSK-ILC-94	Invasive Lobular Carcinoma	Metastasis	ASXL1	p.S146Y	20	31,016,191	31,016,191
MSK-ILC-94	Invasive Lobular Carcinoma	Metastasis	TOP1	p.E709Q	20	39,750,725	39,750,725
MSK-ILC-94	Invasive Lobular Carcinoma	Metastasis	ERG	p.E246K	21	39,772,526	39,772,526
MSK-ILC-94	Invasive Lobular Carcinoma	Metastasis	BCOR	p.E1338Q	X	39,922,160	39,922,160
MSK-ILC-94	Invasive Lobular Carcinoma	Metastasis	RBM10	p.S781L	X	47,045,016	47,045,016
MSK-ILC-94	Invasive Lobular Carcinoma	Metastasis	GATA1	p.X200_splice	X	48,650,729	48,650,729
MSK-ILC-94	Invasive Lobular Carcinoma	Metastasis	KDM5C	p.S509L	X	53,239,915	53,239,915
MSK-ILC-94	Invasive Lobular Carcinoma	Metastasis	MED12	p.R1467*	X	70,352,372	70,352,372
MSK-ILC-94	Invasive Lobular Carcinoma	Metastasis	CUL3	p.R420*	2	225,368,489	225,368,490
MSK-ILC-94	Invasive Lobular Carcinoma	Metastasis	CDH1	p.L731Ffs*39	16	68,862,100	68,862,100
MSK-ILC-145	Invasive Lobular Carcinoma	Primary	TP53	p.R209Kfs*6	17	7,578,222	7,578,223
MSK-ILC-145	Invasive Lobular Carcinoma	Primary	PIK3CA	p.H1047R	3	178,952,085	178,952,085
MSK-ILC-145	Invasive Lobular Carcinoma	Primary	PARP1	p.D726E	1	226,555,999	226,555,999
MSK-ILC-145	Invasive Lobular Carcinoma	Primary	JAK2	p.G993*	9	5,090,829	5,090,829
MSK-ILC-145	Invasive Lobular Carcinoma	Primary	PTCH1	p.L732V	9	98,231,089	98,231,089
MSK-ILC-145	Invasive Lobular Carcinoma	Primary	SMARCA4	p.V880Gfs*64	19	11,132,421	11,132,422
MSK-ILC-145	Invasive Lobular Carcinoma	Primary	FOXA1	p.H374Pfs*40	14	38,060,869	38,060,870
MSK-ILC-145	Invasive Lobular Carcinoma	Primary	CDH1	p.S337Ffs*12	16	68,846,036	68,846,037
MSK-ILC-145	Invasive Lobular Carcinoma	Primary	ERBB2	p.L755_T759delinsAQSQQ	17	37,880,219	37,880,235
MSK-ILC-101	Invasive Lobular Carcinoma	Metastasis	TBX3	p.E100Gfs*40	12	115,120,709	115,120,710
MSK-ILC-101	Invasive Lobular Carcinoma	Metastasis	ESR1	p.Y537C	6	152,419,923	152,419,923
MSK-ILC-101	Invasive Lobular Carcinoma	Metastasis	PIK3CG	p.N299K	7	106,508,903	106,508,903
MSK-ILC-101	Invasive Lobular Carcinoma	Metastasis	MET	p.S916P	7	116,411,567	116,411,567
MSK-ILC-101	Invasive Lobular Carcinoma	Metastasis	FANCC	p.S540N	9	97,864,047	97,864,047
MSK-ILC-101	Invasive Lobular Carcinoma	Metastasis	TBX3	p.W113*	12	115,120,668	115,120,668
MSK-ILC-101	Invasive Lobular Carcinoma	Metastasis	BRCA2	p.E2650Q	13	32,936,802	32,936,802
MSK-ILC-101	Invasive Lobular Carcinoma	Metastasis	CDH1	p.D221Ifs*29	16	68,842,724	68,842,724
MSK-ILC-10	Invasive Lobular Carcinoma	Metastasis	PIK3CA	p.Q546R	3	178,936,095	178,936,095
MSK-ILC-10	Invasive Lobular Carcinoma	Metastasis	SPOP	p.E79K	17	47,696,713	47,696,713
MSK-ILC-10	Invasive Lobular Carcinoma	Metastasis	TBX3	p.N297H	12	115,115,437	115,115,437
MSK-ILC-10	Invasive Lobular Carcinoma	Metastasis	CDH1	p.T115Nfs*53	16	68,835,752	68,835,753
MSK-ILC-229	Invasive Lobular Carcinoma	Metastasis	PIK3CA	p.N345K	3	178,921,553	178,921,553
MSK-ILC-229	Invasive Lobular Carcinoma	Metastasis	PARP1	p.A995D	1	226,549,222	226,549,222
MSK-ILC-229	Invasive Lobular Carcinoma	Metastasis	SMARCD1	p.C339R	12	50,484,165	50,484,165
MSK-ILC-229	Invasive Lobular Carcinoma	Metastasis	CDH1	p.E410Gfs*7	16	68,847,307	68,847,307
MSK-ILC-214	Invasive Lobular Carcinoma	Primary	PIK3CA	p.E542K	3	178,936,082	178,936,082
MSK-ILC-214	Invasive Lobular Carcinoma	Primary	MAP3K1	p.Q1048*	5	56,178,169	56,178,169
MSK-ILC-214	Invasive Lobular Carcinoma	Primary	MAP3K1	p.F1389Lfs*33	5	56,183,254	56,183,254
MSK-ILC-214	Invasive Lobular Carcinoma	Primary	CDH1	p.D777Tfs*6	16	68,863,589	68,863,589
MSK-ILC-211	Invasive Lobular Carcinoma	Metastasis	PIK3CA	p.E545K	3	178,936,091	178,936,091
MSK-ILC-211	Invasive Lobular Carcinoma	Metastasis	TP53	p.E287*	17	7,577,079	7,577,079
MSK-ILC-211	Invasive Lobular Carcinoma	Metastasis	MAP2K4	p.S184L	17	12,011,144	12,011,144
MSK-ILC-211	Invasive Lobular Carcinoma	Metastasis	CDH1	p.E659*	16	68,857,340	68,857,340
MSK-ILC-220	Invasive Lobular Carcinoma	Primary	DNMT1	p.T780M	19	10,260,328	10,260,328
MSK-ILC-220	Invasive Lobular Carcinoma	Primary	SPOP	p.D130H	17	47,696,435	47,696,435
MSK-ILC-220	Invasive Lobular Carcinoma	Primary	CDH1	p.X722_splice	16	68,857,531	68,857,531
MSK-ILC-220	Invasive Lobular Carcinoma	Primary	RUNX1	p.T121Pfs*4	21	36,252,920	36,252,920
MSK-ILC-222	Invasive Lobular Carcinoma	Primary	AKT1	p.E17K	14	105,246,551	105,246,551
MSK-ILC-222	Invasive Lobular Carcinoma	Primary	TP53	p.V73Rfs*76	17	7,579,471	7,579,472
MSK-ILC-222	Invasive Lobular Carcinoma	Primary	TET2	p.I1140T	4	106,162,505	106,162,505
MSK-ILC-91	Invasive Lobular Carcinoma	Metastasis	ERBB2	p.E770_A771insGIRD	17	37,880,982	37,880,983
MSK-ILC-91	Invasive Lobular Carcinoma	Metastasis	ARID1A	p.Q372*	1	27,024,008	27,024,008
MSK-ILC-91	Invasive Lobular Carcinoma	Metastasis	CBFB	p.X133_splice	16	67,100,702	67,100,702
MSK-ILC-91	Invasive Lobular Carcinoma	Metastasis	CDH1	p.E497Rfs*25	16	68,849,586	68,849,586
MSK-ILC-192	Invasive Lobular Carcinoma	Metastasis	PIK3CA	p.E542K	3	178,936,082	178,936,082
MSK-ILC-192	Invasive Lobular Carcinoma	Metastasis	PIK3CA	p.E726K	3	178,938,934	178,938,934
MSK-ILC-192	Invasive Lobular Carcinoma	Metastasis	YAP1	p.Q372*	11	102,094,434	102,094,434
MSK-ILC-192	Invasive Lobular Carcinoma	Metastasis	MSH6	p.S682C	2	48,027,167	48,027,167
MSK-ILC-192	Invasive Lobular Carcinoma	Metastasis	CTNNB1	p.D727H	3	41,280,666	41,280,666
MSK-ILC-192	Invasive Lobular Carcinoma	Metastasis	TERT	p.S796C	5	1,271,315	1,271,315
MSK-ILC-192	Invasive Lobular Carcinoma	Metastasis	NOTCH3	p.S1580L	19	15,281,634	15,281,634
MSK-ILC-192	Invasive Lobular Carcinoma	Metastasis	CDH1	p.G500Wfs*37	16	68,849,592	68,849,593
MSK-ILC-192	Invasive Lobular Carcinoma	Metastasis	TBX3	p.I125Vfs*14	12	115,120,635	115,120,636
MSK-ILC-206	Invasive Lobular Carcinoma	Primary	RICTOR	p.I461L	5	38,984,913	38,984,913
MSK-ILC-206	Invasive Lobular Carcinoma	Primary	CDH1	p.D198Efs*18	16	68,842,657	68,842,658
MSK-ILC-226	Invasive Lobular Carcinoma	Metastasis	ERBB2	p.E770_A771insGIRD	17	37,880,982	37,880,983
MSK-ILC-226	Invasive Lobular Carcinoma	Metastasis	FOXA1	p.D226N	14	38,061,313	38,061,313
MSK-ILC-226	Invasive Lobular Carcinoma	Metastasis	CDH1	p.A439Qfs*16	16	68,847,393	68,847,393
MSK-ILC-226	Invasive Lobular Carcinoma	Metastasis	FLT4	p.R1354H	5	180,030,223	180,030,223
MSK-ILC-176	Invasive Lobular Carcinoma	Primary	PIK3CA	p.H1047R	3	178,952,085	178,952,085
MSK-ILC-176	Invasive Lobular Carcinoma	Primary	CDH1	p.I650Yfs*13	16	68,857,312	68,857,313
MSK-ILC-176	Invasive Lobular Carcinoma	Primary	RUNX1	p.Q237*	21	36,206,722	36,206,722
MSK-ILC-176	Invasive Lobular Carcinoma	Primary	FOXA1	p.S250_G251insTGRCTRTP	14	38,061,239	38,061,240
MSK-ILC-176	Invasive Lobular Carcinoma	Primary	TBX3	p.L242Afs*6	12	115,117,425	115,117,450
MSK-ILC-41	Invasive Lobular Carcinoma	Metastasis	ERBB2	p.L869R	17	37,881,414	37,881,414
MSK-ILC-41	Invasive Lobular Carcinoma	Metastasis	CDH1	p.G808Wfs*9	16	68,863,682	68,863,683
MSK-ILC-137	Invasive Lobular Carcinoma	Metastasis	CDH1	p.R734Kfs*14	16	68,862,112	68,862,113
MSK-ILC-137	Invasive Lobular Carcinoma	Metastasis	ASXL2	p.G10Afs*12	2	26,101,063	26,101,063
MSK-ILC-80	Invasive Lobular Carcinoma	Metastasis	AKT1	p.E17K	14	105,246,551	105,246,551
MSK-ILC-80	Invasive Lobular Carcinoma	Metastasis	CDH1	p.D418Efs*27	16	68,847,332	68,847,333
MSK-ILC-227	Invasive Lobular Carcinoma	Primary	PIK3CA	p.E545K	3	178,936,091	178,936,091
MSK-ILC-227	Invasive Lobular Carcinoma	Primary	FAT1	p.E3234K	4	187,532,693	187,532,693
MSK-ILC-227	Invasive Lobular Carcinoma	Primary	MTOR	p.X2072_splice	1	11,187,680	11,187,680
MSK-ILC-227	Invasive Lobular Carcinoma	Primary	CDH1	p.L721Pfs*27	16	68,857,526	68,857,527
MSK-ILC-227	Invasive Lobular Carcinoma	Primary	NF1	p.Q756H	17	29,554,252	29,554,252
MSK-ILC-227	Invasive Lobular Carcinoma	Primary	PIK3CB	p.L557F	3	138,417,850	138,417,850
MSK-ILC-227	Invasive Lobular Carcinoma	Primary	FLT4	p.R745C	5	180,047,942	180,047,942
MSK-ILC-227	Invasive Lobular Carcinoma	Primary	NOTCH4	p.D404N	6	32,188,011	32,188,011
MSK-ILC-227	Invasive Lobular Carcinoma	Primary	ATRX	p.D1287N	X	76,920,218	76,920,218
MSK-ILC-212	Invasive Lobular Carcinoma	Metastasis	PIK3CA	p.E545K	3	178,936,091	178,936,091
MSK-ILC-212	Invasive Lobular Carcinoma	Metastasis	TP53	p.X332_splice	17	7,574,034	7,574,034
MSK-ILC-212	Invasive Lobular Carcinoma	Metastasis	CDH1	p.G577Efs*7	16	68,855,921	68,855,921
MSK-ILC-212	Invasive Lobular Carcinoma	Metastasis	TP53	p.M246I	17	7,577,543	7,577,543
MSK-ILC-212	Invasive Lobular Carcinoma	Metastasis	HNF1A	p.R131Q	12	121,426,701	121,426,701
MSK-ILC-212	Invasive Lobular Carcinoma	Metastasis	EPHB1	p.S948L	3	134,968,330	134,968,330
MSK-ILC-212	Invasive Lobular Carcinoma	Metastasis	ARID5B	p.H836Y	10	63,851,728	63,851,728
MSK-ILC-212	Invasive Lobular Carcinoma	Metastasis	RNF43	p.S766L	17	56,434,840	56,434,840
MSK-ILC-212	Invasive Lobular Carcinoma	Metastasis	PAK7	p.Y272F	20	9,560,967	9,560,967
MSK-ILC-212	Invasive Lobular Carcinoma	Metastasis	ATRX	p.Y2391C	X	76,776,294	76,776,294
MSK-ILC-112	Invasive Lobular Carcinoma	Primary	PIK3CA	p.E542K	3	178,936,082	178,936,082
MSK-ILC-112	Invasive Lobular Carcinoma	Primary	ARID5B	p.D159V	10	63,700,141	63,700,141
MSK-ILC-112	Invasive Lobular Carcinoma	Primary	MAP2K4	p.P268S	17	12,016,666	12,016,666
MSK-ILC-112	Invasive Lobular Carcinoma	Primary	CDH1	p.G62Rfs*32	16	68,835,591	68,835,592
MSK-ILC-119	Invasive Lobular Carcinoma	Metastasis	TP53	p.R248Q	17	7,577,538	7,577,538
MSK-ILC-119	Invasive Lobular Carcinoma	Metastasis	PIK3CA	p.N345K	3	178,921,553	178,921,553
MSK-ILC-119	Invasive Lobular Carcinoma	Metastasis	TBX3	p.E122K	12	115,120,642	115,120,642
MSK-ILC-119	Invasive Lobular Carcinoma	Metastasis	TSC2	p.V1711M	16	2,138,111	2,138,111
MSK-ILC-119	Invasive Lobular Carcinoma	Metastasis	ROS1	p.Q1114E	6	117,683,807	117,683,807
MSK-ILC-15	Invasive Lobular Carcinoma	Metastasis	PIK3CA	p.E545K	3	178,936,091	178,936,091
MSK-ILC-15	Invasive Lobular Carcinoma	Metastasis	NCOR1	p.T525Nfs*11	17	16,029,457	16,029,458
MSK-ILC-15	Invasive Lobular Carcinoma	Metastasis	SMAD4	p.L540del	18	48,604,796	48,604,798
MSK-ILC-15	Invasive Lobular Carcinoma	Metastasis	NF1	p.R416*	17	29,528,489	29,528,489
MSK-ILC-15	Invasive Lobular Carcinoma	Metastasis	JUN	p.E112Q	1	59,248,409	59,248,409
MSK-ILC-15	Invasive Lobular Carcinoma	Metastasis	MTOR	p.E595Q	1	11,300,363	11,300,363
MSK-ILC-15	Invasive Lobular Carcinoma	Metastasis	CDH1	p.T295_Q307delinsK	16	68,845,638	68,845,673
MSK-ILC-15	Invasive Lobular Carcinoma	Metastasis	MAP2K4	p.S257C	17	12,016,634	12,016,634
MSK-ILC-15	Invasive Lobular Carcinoma	Metastasis	CIC	p.R1214Q	19	42,797,279	42,797,279
MSK-ILC-15	Invasive Lobular Carcinoma	Metastasis	MITF	p.E234Q	3	69,990,420	69,990,420
MSK-ILC-15	Invasive Lobular Carcinoma	Metastasis	EPHB1	p.A836T	3	134,967,167	134,967,167
MSK-ILC-15	Invasive Lobular Carcinoma	Metastasis	TET2	p.Q232K	4	106,155,793	106,155,793
MSK-ILC-15	Invasive Lobular Carcinoma	Metastasis	APC	p.T1267S	5	112,175,091	112,175,091
MSK-ILC-193	Invasive Lobular Carcinoma	Primary	MTOR	p.M2010I	1	11,188,064	11,188,064
MSK-ILC-193	Invasive Lobular Carcinoma	Primary	CCND1	p.S43C	11	69,456,209	69,456,209
MSK-ILC-193	Invasive Lobular Carcinoma	Primary	ERBB3	p.S846I	12	56,491,645	56,491,645
MSK-ILC-193	Invasive Lobular Carcinoma	Primary	ERBB3	p.E928G	12	56,492,633	56,492,633
MSK-ILC-193	Invasive Lobular Carcinoma	Primary	CREBBP	p.Q2267H	16	3,778,247	3,778,247
MSK-ILC-193	Invasive Lobular Carcinoma	Primary	CTCF	p.C271_S272insKLHVSTAFKFGSSHE	16	67,645,886	67,645,887
MSK-ILC-193	Invasive Lobular Carcinoma	Primary	CDH1	p.N536Ifs*21	16	68,853,221	68,853,221
MSK-ILC-193	Invasive Lobular Carcinoma	Primary	PIK3CA	p.E81K	3	178,916,854	178,916,854
MSK-ILC-196	Invasive Lobular Carcinoma	Primary	PIK3CA	p.H1047R	3	178,952,085	178,952,085
MSK-ILC-118	Invasive Lobular Carcinoma	Metastasis	ERBB2	p.L755S	17	37,880,220	37,880,220
MSK-ILC-118	Invasive Lobular Carcinoma	Metastasis	FOXA1	p.I176V	14	38,061,463	38,061,463
MSK-ILC-118	Invasive Lobular Carcinoma	Metastasis	TBX3	p.A51Gfs*37	12	115,120,854	115,120,854
MSK-ILC-118	Invasive Lobular Carcinoma	Metastasis	CDH1	p.V392Efs*5	16	68,847,253	68,847,253
MSK-ILC-118	Invasive Lobular Carcinoma	Metastasis	RB1	p.R71Kfs*7	13	48,881,489	48,881,490
MSK-ILC-118	Invasive Lobular Carcinoma	Metastasis	GRIN2A	p.X336_splice	16	10,031,815	10,031,815
MSK-ILC-118	Invasive Lobular Carcinoma	Metastasis	KIT	p.R634Q	4	55,594,198	55,594,198
MSK-ILC-218	Invasive Lobular Carcinoma	Primary	AKT1	p.E17K	14	105,246,551	105,246,551
MSK-ILC-218	Invasive Lobular Carcinoma	Primary	CBFB	p.X55_splice	16	67,063,717	67,063,717
MSK-ILC-218	Invasive Lobular Carcinoma	Primary	ERBB2	p.V777L	17	37,881,000	37,881,000
MSK-ILC-218	Invasive Lobular Carcinoma	Primary	CDH1	p.D104Tfs*13	16	68,835,717	68,835,717
MSK-ILC-218	Invasive Lobular Carcinoma	Primary	CDH1	p.H555Rfs*32	16	68,853,280	68,853,281
MSK-ILC-202	Invasive Lobular Carcinoma	Primary	PTEN	p.H123R	10	89,692,884	89,692,884
MSK-ILC-202	Invasive Lobular Carcinoma	Primary	PTEN	p.X164_splice	10	89,693,005	89,693,010
MSK-ILC-202	Invasive Lobular Carcinoma	Primary	CBFB	p.L21R	16	67,063,372	67,063,372
MSK-ILC-202	Invasive Lobular Carcinoma	Primary	CDH1	p.P744Qfs*26	16	68,862,139	68,862,139
MSK-ILC-23	Invasive Lobular Carcinoma	Metastasis	ERBB3	p.E928G	12	56,492,633	56,492,633
MSK-ILC-23	Invasive Lobular Carcinoma	Metastasis	ERBB2	p.L869R	17	37,881,414	37,881,414
MSK-ILC-23	Invasive Lobular Carcinoma	Metastasis	PTCH1	p.V556I	9	98,238,378	98,238,378
MSK-ILC-23	Invasive Lobular Carcinoma	Metastasis	CDH1	p.G81Vfs*2	16	68,835,650	68,835,650
MSK-ILC-23	Invasive Lobular Carcinoma	Metastasis	TBX3	p.R436Tfs*3	12	115,112,435	115,112,436
MSK-ILC-235	Invasive Lobular Carcinoma	Primary	PIK3CA	p.N345K	3	178,921,553	178,921,553
MSK-ILC-235	Invasive Lobular Carcinoma	Primary	CBFB	p.X55_splice	16	67,063,717	67,063,717
MSK-ILC-235	Invasive Lobular Carcinoma	Primary	CDKN1B	p.Y74*	12	12,870,994	12,870,995
MSK-ILC-235	Invasive Lobular Carcinoma	Primary	TBX3	p.Y603Sfs*27	12	115,110,064	115,110,070
MSK-ILC-235	Invasive Lobular Carcinoma	Primary	TBX3	p.M123V	12	115,120,639	115,120,639
MSK-ILC-235	Invasive Lobular Carcinoma	Primary	CDH1	p.X522_splice	16	68,853,179	68,853,187
MSK-ILC-22	Invasive Lobular Carcinoma	Metastasis	AKT1	p.E17K	14	105,246,551	105,246,551
MSK-ILC-22	Invasive Lobular Carcinoma	Metastasis	TP53	p.Q331*	17	7,576,855	7,576,855
MSK-ILC-22	Invasive Lobular Carcinoma	Metastasis	ARID1A	p.A770T	1	27,088,699	27,088,699
MSK-ILC-22	Invasive Lobular Carcinoma	Metastasis	RB1	p.X830_splice	13	49,047,495	49,047,495
MSK-ILC-22	Invasive Lobular Carcinoma	Metastasis	CREBBP	p.F2231L	16	3,778,355	3,778,355
MSK-ILC-22	Invasive Lobular Carcinoma	Metastasis	CBFB	p.L103R	16	67,100,610	67,100,610
MSK-ILC-22	Invasive Lobular Carcinoma	Metastasis	CDH1	p.R54Kfs*5	16	68,772,311	68,772,312
MSK-ILC-22	Invasive Lobular Carcinoma	Metastasis	BCL6	p.C520Y	3	187,444,668	187,444,668
MSK-ILC-124	Invasive Lobular Carcinoma	Metastasis	PTEN	p.L23V	10	89,624,293	89,624,293
MSK-ILC-124	Invasive Lobular Carcinoma	Metastasis	PTEN	p.C124S	10	89,692,887	89,692,887
MSK-ILC-124	Invasive Lobular Carcinoma	Metastasis	FOXA1	p.S250F	14	38,061,240	38,061,240
MSK-ILC-124	Invasive Lobular Carcinoma	Metastasis	CDH1	p.E310*	16	68,845,682	68,845,682
MSK-ILC-124	Invasive Lobular Carcinoma	Metastasis	NTRK1	p.M464I	1	156,845,349	156,845,349
MSK-ILC-124	Invasive Lobular Carcinoma	Metastasis	GATA3	p.H69R	10	8,097,794	8,097,794
MSK-ILC-124	Invasive Lobular Carcinoma	Metastasis	FLT1	p.V960I	13	28,897,002	28,897,002
MSK-ILC-124	Invasive Lobular Carcinoma	Metastasis	MAP2K2	p.S295A	19	4,099,235	4,099,235
MSK-ILC-124	Invasive Lobular Carcinoma	Metastasis	DNMT3B	p.R125Q	20	31,374,375	31,374,375
MSK-ILC-124	Invasive Lobular Carcinoma	Metastasis	PDGFRA	p.T274N	4	55,133,517	55,133,517
MSK-ILC-205	Invasive Lobular Carcinoma	Primary	PIK3CA	p.H1047R	3	178,952,085	178,952,085
MSK-ILC-205	Invasive Lobular Carcinoma	Primary	MEN1	p.L89R	11	64,577,316	64,577,316
MSK-ILC-205	Invasive Lobular Carcinoma	Primary	CDH1	p.X278_splice	16	68,844,245	68,844,245
MSK-ILC-197	Invasive Lobular Carcinoma	Primary	PIK3CA	p.N345K	3	178,921,553	178,921,553
MSK-ILC-197	Invasive Lobular Carcinoma	Primary	CDH1	p.Q23*	16	68,772,218	68,772,218
MSK-ILC-197	Invasive Lobular Carcinoma	Primary	CBFB	p.F44V	16	67,063,681	67,063,681
MSK-ILC-111	Invasive Lobular Carcinoma	Primary	PIK3CA	p.E545K	3	178,936,091	178,936,091
MSK-ILC-111	Invasive Lobular Carcinoma	Primary	CDH1	p.S270Lfs*12	16	68,844,220	68,844,220
MSK-ILC-35	Invasive Lobular Carcinoma	Primary	PIK3CA	p.H1047R	3	178,952,085	178,952,085
MSK-ILC-35	Invasive Lobular Carcinoma	Primary	MED12	p.R1496H	X	70,352,766	70,352,766
MSK-ILC-35	Invasive Lobular Carcinoma	Primary	STAG2	p.G1062E	X	123,220,528	123,220,528
MSK-ILC-35	Invasive Lobular Carcinoma	Primary	CDH1	p.D291Hfs*11	16	68,845,624	68,845,649
MSK-ILC-48	Invasive Lobular Carcinoma	Metastasis	PIK3CA	p.H1047R	3	178,952,085	178,952,085
MSK-ILC-48	Invasive Lobular Carcinoma	Metastasis	MYD88	p.S243N	3	38,182,292	38,182,292
MSK-ILC-175	Invasive Lobular Carcinoma	Primary	PIK3CA	p.E542K	3	178,936,082	178,936,082
MSK-ILC-175	Invasive Lobular Carcinoma	Primary	CDH1	p.L681*	16	68,857,407	68,857,407
MSK-ILC-177	Invasive Lobular Carcinoma	Primary	PIK3CA	p.E545K	3	178,936,091	178,936,091
MSK-ILC-177	Invasive Lobular Carcinoma	Primary	CDH1	p.Q23*	16	68,772,218	68,772,218
MSK-ILC-177	Invasive Lobular Carcinoma	Primary	SOX9	p.E75K	17	70,117,755	70,117,755
MSK-ILC-3	Invasive Lobular Carcinoma	Metastasis	ESR1	p.E380Q	6	152,332,832	152,332,832
MSK-ILC-3	Invasive Lobular Carcinoma	Metastasis	REL	p.S409F	2	61,149,036	61,149,036
MSK-ILC-3	Invasive Lobular Carcinoma	Metastasis	DAXX	p.S405C	6	33,288,194	33,288,194
MSK-ILC-3	Invasive Lobular Carcinoma	Metastasis	GATA3	p.X308_splice	10	8,111,433	8,111,434
MSK-ILC-3	Invasive Lobular Carcinoma	Metastasis	NOTCH2	p.D2435H	1	120,458,042	120,458,042
MSK-ILC-3	Invasive Lobular Carcinoma	Metastasis	IKBKE	p.M562I	1	206,661,320	206,661,320
MSK-ILC-3	Invasive Lobular Carcinoma	Metastasis	RPS6KA4	p.X535_splice	11	64,137,170	64,137,170
MSK-ILC-3	Invasive Lobular Carcinoma	Metastasis	AKT1	p.E98*	14	105,242,132	105,242,132
MSK-ILC-3	Invasive Lobular Carcinoma	Metastasis	CDH1	p.S70Dfs*9	16	68,835,617	68,835,629
MSK-ILC-3	Invasive Lobular Carcinoma	Metastasis	NF1	p.S1443L	17	29,585,516	29,585,516
MSK-ILC-3	Invasive Lobular Carcinoma	Metastasis	RUNX1	p.R142_G143insEE	21	36,252,853	36,252,854
MSK-ILC-3	Invasive Lobular Carcinoma	Metastasis	KIT	p.R135C	4	55,564,515	55,564,515
MSK-ILC-3	Invasive Lobular Carcinoma	Metastasis	FAT1	p.E38K	4	187,630,870	187,630,870
MSK-ILC-3	Invasive Lobular Carcinoma	Metastasis	TSC1	p.S832F	9	135,776,983	135,776,983
MSK-ILC-213	Invasive Lobular Carcinoma	Primary	PIK3CA	p.E545K	3	178,936,091	178,936,091
MSK-ILC-213	Invasive Lobular Carcinoma	Primary	TP53	p.E336*	17	7,574,021	7,574,021
MSK-ILC-213	Invasive Lobular Carcinoma	Primary	FH	p.R6W	1	241,683,007	241,683,007
MSK-ILC-213	Invasive Lobular Carcinoma	Primary	ARID1A	p.S2269*	1	27,107,195	27,107,195
MSK-ILC-213	Invasive Lobular Carcinoma	Primary	FOXA1	p.I176M	14	38,061,461	38,061,461
MSK-ILC-213	Invasive Lobular Carcinoma	Primary	CDH1	p.T330Nfs*20	16	68,845,742	68,845,743
MSK-ILC-213	Invasive Lobular Carcinoma	Primary	TP53	p.E28K	17	7,579,714	7,579,714
MSK-ILC-213	Invasive Lobular Carcinoma	Primary	DOT1L	p.E655Q	19	2,216,319	2,216,319
MSK-ILC-213	Invasive Lobular Carcinoma	Primary	DOT1L	p.E710K	19	2,216,484	2,216,484
MSK-ILC-213	Invasive Lobular Carcinoma	Primary	DOT1L	p.D744N	19	2,216,586	2,216,586
MSK-ILC-213	Invasive Lobular Carcinoma	Primary	EPHB1	p.K643N	3	134,911,464	134,911,464
MSK-ILC-213	Invasive Lobular Carcinoma	Primary	MAP3K1	p.X1223_splice	5	56,179,353	56,179,353
MSK-ILC-213	Invasive Lobular Carcinoma	Primary	NSD1	p.P1209A	5	176,639,025	176,639,025
MSK-ILC-213	Invasive Lobular Carcinoma	Primary	NSD1	p.S1240C	5	176,639,119	176,639,119
MSK-ILC-213	Invasive Lobular Carcinoma	Primary	EZH2	p.H129D	7	148,526,919	148,526,919
MSK-ILC-213	Invasive Lobular Carcinoma	Primary	TSC1	p.H279N	9	135,787,747	135,787,747
MSK-ILC-21	Invasive Lobular Carcinoma	Primary	PIK3CA	p.E545K	3	178,936,091	178,936,091
MSK-ILC-21	Invasive Lobular Carcinoma	Primary	CDKN2A	p.A102V	9	21,971,053	21,971,053
MSK-ILC-21	Invasive Lobular Carcinoma	Primary	CDH1	p.D750Rfs*5	16	68,862,159	68,862,163
MSK-ILC-102	Invasive Lobular Carcinoma	Metastasis	PIK3CA	p.E542K	3	178,936,082	178,936,082
MSK-ILC-102	Invasive Lobular Carcinoma	Metastasis	TP53	p.E285K	17	7,577,085	7,577,085
MSK-ILC-102	Invasive Lobular Carcinoma	Metastasis	PIK3CA	p.E726K	3	178,938,934	178,938,934
MSK-ILC-102	Invasive Lobular Carcinoma	Metastasis	CTCF	p.F351L	16	67,650,748	67,650,748
MSK-ILC-102	Invasive Lobular Carcinoma	Metastasis	FOXA1	p.F266L	14	38,061,191	38,061,191
MSK-ILC-102	Invasive Lobular Carcinoma	Metastasis	LATS2	p.D1048N	13	21,549,134	21,549,134
MSK-ILC-102	Invasive Lobular Carcinoma	Metastasis	CDH1	p.T40Rfs*9	16	68,772,269	68,772,290
MSK-ILC-102	Invasive Lobular Carcinoma	Metastasis	NF1	p.S365*	17	29,528,086	29,528,086
MSK-ILC-102	Invasive Lobular Carcinoma	Metastasis	U2AF1	p.Q31H	21	44,524,466	44,524,466
MSK-ILC-102	Invasive Lobular Carcinoma	Metastasis	FBXW7	p.M268I	4	153,259,011	153,259,011
MSK-ILC-102	Invasive Lobular Carcinoma	Metastasis	FGFR1	p.E137*	8	38,285,903	38,285,903
MSK-ILC-102	Invasive Lobular Carcinoma	Metastasis	NBN	p.D61*	8	90,993,741	90,993,742
MSK-ILC-102	Invasive Lobular Carcinoma	Metastasis	JAK2	p.D840H	9	5,081,808	5,081,808
MSK-ILC-34	Invasive Lobular Carcinoma	Primary	PIK3CA	p.E453K	3	178,928,079	178,928,079
MSK-ILC-34	Invasive Lobular Carcinoma	Primary	MED12	p.G44D	X	70,339,254	70,339,254
MSK-ILC-34	Invasive Lobular Carcinoma	Primary	GATA3	p.X308_splice	10	8,111,433	8,111,434
MSK-ILC-34	Invasive Lobular Carcinoma	Primary	GATA3	p.S212A	10	8,100,660	8,100,660
MSK-ILC-34	Invasive Lobular Carcinoma	Primary	CDH1	p.S635Kfs*34	16	68,856,095	68,856,096
MSK-ILC-34	Invasive Lobular Carcinoma	Primary	MAP2K4	p.L76Qfs*11	17	11,984,681	11,984,684
MSK-ILC-34	Invasive Lobular Carcinoma	Primary	MAP3K1	p.D1388G	5	56,183,253	56,183,253
MSK-ILC-34	Invasive Lobular Carcinoma	Primary	TBX3	p.D326Efs*12	12	115,114,237	115,114,239
MSK-ILC-225	Invasive Lobular Carcinoma	Primary	TGFBR1	p.A26del	9	101,867,538	101,867,540
MSK-ILC-225	Invasive Lobular Carcinoma	Primary	ERBB2	p.L869R	17	37,881,414	37,881,414
MSK-ILC-225	Invasive Lobular Carcinoma	Primary	CDH1	p.R733Efs*15	16	68,862,106	68,862,107
MSK-ILC-225	Invasive Lobular Carcinoma	Primary	PIK3CA	p.Y1021N	3	178,952,006	178,952,006
MSK-ILC-225	Invasive Lobular Carcinoma	Primary	MAP3K1	p.Q1007Rfs*75	5	56,178,044	56,178,044
MSK-ILC-225	Invasive Lobular Carcinoma	Primary	XIAP	p.R443C	X	123,040,864	123,040,864
MSK-ILC-208	Invasive Lobular Carcinoma	Primary	AKT1	p.E17K	14	105,246,551	105,246,551
MSK-ILC-208	Invasive Lobular Carcinoma	Primary	CDH1	p.C603Lfs*2	16	68,855,996	68,855,997
MSK-ILC-208	Invasive Lobular Carcinoma	Primary	CBFB	p.P70Sfs*13	16	67,070,578	67,070,579
MSK-ILC-203	Invasive Lobular Carcinoma	Primary	PIK3CA	p.G118D	3	178,917,478	178,917,478
MSK-ILC-203	Invasive Lobular Carcinoma	Primary	ERBB2	p.L755S	17	37,880,220	37,880,220
MSK-ILC-203	Invasive Lobular Carcinoma	Primary	CDH1	p.Q23*	16	68,772,218	68,772,218
MSK-ILC-203	Invasive Lobular Carcinoma	Primary	ARID1A	p.E2246*	1	27,107,125	27,107,125
MSK-ILC-203	Invasive Lobular Carcinoma	Primary	STK11	p.Q159*	19	1,220,382	1,220,382
MSK-ILC-203	Invasive Lobular Carcinoma	Primary	PTPRS	p.G973S	19	5,222,886	5,222,886
MSK-ILC-203	Invasive Lobular Carcinoma	Primary	SETD2	p.K2115Nfs*32	3	47,098,929	47,098,929
MSK-ILC-203	Invasive Lobular Carcinoma	Primary	PDGFRB	p.X456_splice	5	149,510,101	149,510,101
MSK-ILC-236	Invasive Lobular Carcinoma	Primary	AKT1	p.E17K	14	105,246,551	105,246,551
MSK-ILC-236	Invasive Lobular Carcinoma	Primary	RUNX1	p.L71Sfs*24	21	36,259,199	36,259,199
MSK-ILC-236	Invasive Lobular Carcinoma	Primary	CDH1	p.L585Cfs*4	16	68,855,944	68,855,944
MSK-ILC-237	Invasive Lobular Carcinoma	Primary	PIK3CA	p.Q546K	3	178,936,094	178,936,094
MSK-ILC-237	Invasive Lobular Carcinoma	Primary	FOXA1	p.R261S	14	38,061,208	38,061,208
MSK-ILC-237	Invasive Lobular Carcinoma	Primary	CDH1	p.Q503*	16	68,849,604	68,849,604
MSK-ILC-237	Invasive Lobular Carcinoma	Primary	MAP3K1	p.R1368G	5	56,181,878	56,181,878
MSK-ILC-73	Invasive Lobular Carcinoma	Metastasis	PIK3CA	p.H1047R	3	178,952,085	178,952,085
MSK-ILC-73	Invasive Lobular Carcinoma	Metastasis	ESR1	p.Y537S	6	152,419,923	152,419,923
MSK-ILC-73	Invasive Lobular Carcinoma	Metastasis	CDH1	p.Q610*	16	68,856,020	68,856,020
MSK-ILC-73	Invasive Lobular Carcinoma	Metastasis	NTRK1	p.R761Q	1	156,851,325	156,851,325
MSK-ILC-73	Invasive Lobular Carcinoma	Metastasis	RICTOR	p.G1511E	5	38,945,694	38,945,694
MSK-ILC-73	Invasive Lobular Carcinoma	Metastasis	LATS2	p.D1047H	13	21,549,137	21,549,137
MSK-ILC-216	Invasive Lobular Carcinoma	Primary	PIK3CA	p.N345K	3	178,921,553	178,921,553
MSK-ILC-216	Invasive Lobular Carcinoma	Primary	CDH1	p.P127Afs*41	16	68,835,781	68,835,782
MSK-ILC-231	Invasive Lobular Carcinoma	Primary	PIK3CA	p.H1047R	3	178,952,085	178,952,085
MSK-ILC-231	Invasive Lobular Carcinoma	Primary	CDH1	p.D514Y	16	68,849,637	68,849,637
MSK-ILC-234	Invasive Lobular Carcinoma	Primary	PIK3CA	p.E545K	3	178,936,091	178,936,091
MSK-ILC-234	Invasive Lobular Carcinoma	Primary	ERBB2	p.L755S	17	37,880,220	37,880,220
MSK-ILC-234	Invasive Lobular Carcinoma	Primary	BRAF	p.S125F	7	140,534,539	140,534,539
MSK-ILC-234	Invasive Lobular Carcinoma	Primary	RUNX1	p.Q237*	21	36,206,722	36,206,722
MSK-ILC-234	Invasive Lobular Carcinoma	Primary	CDH1	p.D288Efs*6	16	68,845,618	68,845,618
MSK-ILC-234	Invasive Lobular Carcinoma	Primary	NCOR1	p.E975K	17	15,995,270	15,995,270
MSK-ILC-234	Invasive Lobular Carcinoma	Primary	INPP4A	p.E87D	2	99,149,949	99,149,949
MSK-ILC-207	Invasive Lobular Carcinoma	Metastasis	MAP3K1	p.Q1020*	5	56,178,085	56,178,085
MSK-ILC-207	Invasive Lobular Carcinoma	Metastasis	CDH1	p.V17Gfs*17	16	68,772,199	68,772,200
MSK-ILC-198	Invasive Lobular Carcinoma	Primary	AKT1	p.E17K	14	105,246,551	105,246,551
MSK-ILC-198	Invasive Lobular Carcinoma	Primary	NOTCH1	p.R504H	9	139,411,768	139,411,768
MSK-ILC-198	Invasive Lobular Carcinoma	Primary	CDH1	p.R63*	16	68,835,596	68,835,596
MSK-ILC-198	Invasive Lobular Carcinoma	Primary	GATA3	p.C338Afs*17	10	8,111,526	8,111,526
MSK-ILC-198	Invasive Lobular Carcinoma	Primary	CBFB	p.M101V	16	67,100,603	67,100,603
MSK-ILC-2	Invasive Lobular Carcinoma	Metastasis	PIK3CA	p.E545K	3	178,936,091	178,936,091
MSK-ILC-2	Invasive Lobular Carcinoma	Metastasis	CDH1	p.R54*	16	68,772,311	68,772,311
MSK-ILC-2	Invasive Lobular Carcinoma	Metastasis	ARID1A	p.L192Pfs*208	1	27,023,462	27,023,463
MSK-ILC-2	Invasive Lobular Carcinoma	Metastasis	PTEN	p.I50T	10	89,653,851	89,653,851
MSK-ILC-2	Invasive Lobular Carcinoma	Metastasis	NCOR1	p.X1796_splice	17	15,965,417	15,965,417
MSK-ILC-2	Invasive Lobular Carcinoma	Metastasis	SF3B1	p.Q931E	2	198,265,086	198,265,086
MSK-ILC-2	Invasive Lobular Carcinoma	Metastasis	RUNX1	p.R174Efs*10	21	36,231,787	36,231,787
MSK-ILC-2	Invasive Lobular Carcinoma	Metastasis	BCOR	p.K1104*	X	39,923,781	39,923,781
MSK-ILC-178	Invasive Lobular Carcinoma	Metastasis	ERBB2	p.L755S	17	37,880,220	37,880,220
MSK-ILC-178	Invasive Lobular Carcinoma	Metastasis	NCOR1	p.R350*	17	16,049,724	16,049,724
MSK-ILC-178	Invasive Lobular Carcinoma	Metastasis	RUNX1	p.Y328Lfs*245	21	36,164,813	36,164,814
MSK-ILC-178	Invasive Lobular Carcinoma	Metastasis	TBX3	p.E122Lfs*14	12	115,120,634	115,120,643
MSK-ILC-178	Invasive Lobular Carcinoma	Metastasis	CDH1	p.Y107*	16	68,835,729	68,835,730
MSK-ILC-13	Invasive Lobular Carcinoma	Primary	CTCF	p.H477Y	16	67,660,529	67,660,529
MSK-ILC-13	Invasive Lobular Carcinoma	Primary	CDH1	p.Y523*	16	68,853,186	68,853,193
MSK-ILC-201	Invasive Lobular Carcinoma	Primary	PIK3CA	p.E542K	3	178,936,082	178,936,082
MSK-ILC-201	Invasive Lobular Carcinoma	Primary	PIK3CA	p.E726K	3	178,938,934	178,938,934
MSK-ILC-201	Invasive Lobular Carcinoma	Primary	NF1	p.Q1370*	17	29,576,135	29,576,135
MSK-ILC-182	Invasive Lobular Carcinoma	Primary	PIK3CA	p.H1047R	3	178,952,085	178,952,085
MSK-ILC-182	Invasive Lobular Carcinoma	Primary	CDH1	p.Y228*	16	68,842,748	68,842,748
MSK-ILC-182	Invasive Lobular Carcinoma	Primary	MAP3K1	p.P1206Lfs*39	5	56,178,641	56,178,641
MSK-ILC-182	Invasive Lobular Carcinoma	Primary	KDM6A	p.W1096Vfs*7	X	44,942,030	44,942,031
MSK-ILC-36	Invasive Lobular Carcinoma	Primary	ARID1A	p.R1722*	1	27,105,553	27,105,553
MSK-ILC-36	Invasive Lobular Carcinoma	Primary	CDH1	p.N570Kfs*14	16	68,853,327	68,853,327
MSK-ILC-36	Invasive Lobular Carcinoma	Primary	PIK3CA	p.H450_L455del	3	178,928,066	178,928,086
MSK-ILC-36	Invasive Lobular Carcinoma	Primary	SMO	p.N608S	7	128,851,498	128,851,498
MSK-ILC-217	Invasive Lobular Carcinoma	Metastasis	ERBB2	p.G778_S779insLPG	17	37,881,002	37,881,003
MSK-ILC-217	Invasive Lobular Carcinoma	Metastasis	CIC	p.G823E	19	42,794,788	42,794,788
MSK-ILC-217	Invasive Lobular Carcinoma	Metastasis	TBX3	p.E195Nfs*13	12	115,118,758	115,118,758
MSK-ILC-217	Invasive Lobular Carcinoma	Metastasis	CDH1	p.X722_splice	16	68,857,530	68,857,531
MSK-ILC-210	Invasive Lobular Carcinoma	Primary	CDH1	p.K491Efs*30	16	68,849,567	68,849,570
MSK-ILC-210	Invasive Lobular Carcinoma	Primary	RICTOR	p.S1016L	5	38,952,378	38,952,378
MSK-ILC-224	Invasive Lobular Carcinoma	Primary	PIK3CA	p.N107I	3	178,916,933	178,916,933
MSK-ILC-224	Invasive Lobular Carcinoma	Primary	RB1	p.T726Nfs*25	13	49,037,934	49,037,935
MSK-ILC-224	Invasive Lobular Carcinoma	Primary	NCOR1	p.L57_Q61delins*	17	16,089,929	16,089,940
MSK-ILC-20	Invasive Lobular Carcinoma	Metastasis	PIK3CA	p.E545K	3	178,936,091	178,936,091

MSK-ILC-20	Invasive Lobular Carcinoma	Metastasis	FGFR4	p.N535K	5	176,522,416	176,522,416	C	C	G	Missense_Mutation	TRUE	FALSE
MSK-ILC-20	Invasive Lobular Carcinoma	Metastasis	CCND2	p.R178C	12	4,388,046	4,388,046	C	C	T	Missense_Mutation	FALSE	FALSE
MSK-ILC-20	Invasive Lobular Carcinoma	Metastasis	ERCC2	p.A580T	19	45,856,520	45,856,520	C	C	T	Missense_Mutation	FALSE	FALSE
MSK-ILC-20	Invasive Lobular Carcinoma	Metastasis	CDH1	p.L742Tfs*6	16	68,862,135	68,862,136	-	-	A	Frame_Shift_Ins	FALSE	TRUE
MSK-ILC-228	Invasive Lobular Carcinoma	Metastasis	SPEN	p.Q1343*	1	16,256,762	16,256,762	C	C	T	Nonsense_Mutation	FALSE	FALSE
MSK-ILC-228	Invasive Lobular Carcinoma	Metastasis	CDH1	p.L658*	16	68,857,335	68,857,335	C	C	-	Frame_Shift_Del	FALSE	TRUE
MSK-ILC-64	Invasive Lobular Carcinoma	Metastasis	AKT1	p.E17K	14	105,246,551	105,246,551	C	C	T	Missense_Mutation	TRUE	FALSE
MSK-ILC-64	Invasive Lobular Carcinoma	Metastasis	FGFR4	p.N535K	5	176,522,416	176,522,416	C	C	A	Missense_Mutation	TRUE	FALSE
MSK-ILC-64	Invasive Lobular Carcinoma	Metastasis	MAP2K1	p.S123P	15	66,729,159	66,729,159	T	T	C	Missense_Mutation	FALSE	FALSE
MSK-ILC-64	Invasive Lobular Carcinoma	Metastasis	CREBBP	p.V1307M	16	3,794,958	3,794,958	C	C	T	Missense_Mutation	FALSE	FALSE
MSK-ILC-64	Invasive Lobular Carcinoma	Metastasis	CDH1	p.Y37C	16	68,772,261	68,772,261	A	A	G	Missense_Mutation	FALSE	TRUE
MSK-ILC-64	Invasive Lobular Carcinoma	Metastasis	CDH1	p.P708Lfs*14	16	68,857,487	68,857,487	C	C	-	Frame_Shift_Del	FALSE	TRUE
MSK-ILC-64	Invasive Lobular Carcinoma	Metastasis	XPO1	p.R765Q	2	61,715,319	61,715,319	C	C	T	Missense_Mutation	FALSE	FALSE
MSK-ILC-64	Invasive Lobular Carcinoma	Metastasis	BTK	p.W421*	X	100,611,858	100,611,858	C	C	-	Frame_Shift_Del	FALSE	FALSE
MSK-META-11	Metaplastic Carcinoma	Primary	TP53	p.R213*	17	7,578,212	7,578,212	G	G	A	Nonsense_Mutation	TRUE	TRUE
MSK-META-11	Metaplastic Carcinoma	Primary	BRCA2	p.L1292_Q1295del	13	32,912,363	32,912,374	CAACTGATATTA	CAACTGATATTA	-	In_Frame_Del	FALSE	FALSE
MSK-META-11	Metaplastic Carcinoma	Primary	NOTCH3	p.P2293S	19	15,271,562	15,271,562	G	G	A	Missense_Mutation	FALSE	FALSE
MSK-META-8	Metaplastic Carcinoma	Metastasis	TP53	p.C242Afs*5	17	7,577,558	7,577,558	G	G	-	Frame_Shift_Del	FALSE	TRUE
MSK-META-8	Metaplastic Carcinoma	Metastasis	NF1	p.V212L	17	29,508,487	29,508,487	G	G	C	Missense_Mutation	FALSE	FALSE
MSK-META-8	Metaplastic Carcinoma	Metastasis	ASXL1	p.M1161V	20	31,023,996	31,023,996	A	A	G	Missense_Mutation	FALSE	FALSE
MSK-META-5	Metaplastic Carcinoma	Primary	FBXW7	p.X473_splice	4	153,249,358	153,249,358	A	A	C	Splice_Site	FALSE	TRUE
MSK-META-5	Metaplastic Carcinoma	Primary	FLT4	p.V964L	5	180,045,881	180,045,881	C	C	A	Missense_Mutation	FALSE	FALSE
MSK-META-5	Metaplastic Carcinoma	Primary	RBM10	p.R230G	X	47,038,526	47,038,526	C	C	G	Missense_Mutation	FALSE	TRUE
MSK-META-5	Metaplastic Carcinoma	Primary	TP53	p.I232Sfs*15	17	7,577,587	7,577,587	T	T	-	Frame_Shift_Del	FALSE	FALSE
MSK-META-9	Metaplastic Carcinoma	Primary	CDKN2A	p.G35V	9	21,974,723	21,974,723	C	C	A	Missense_Mutation	FALSE	TRUE
MSK-META-9	Metaplastic Carcinoma	Primary	TP53	p.Y220*	17	7,578,189	7,578,189	A	A	C	Nonsense_Mutation	TRUE	TRUE
MSK-META-9	Metaplastic Carcinoma	Primary	NF1	p.D1028Y	17	29,557,369	29,557,369	G	G	T	Missense_Mutation	FALSE	FALSE
MSK-META-6	Metaplastic Carcinoma	Primary	TP53	p.R175H	17	7,578,406	7,578,406	C	C	T	Missense_Mutation	TRUE	TRUE
MSK-META-10	Metaplastic Carcinoma	Metastasis	PIK3CA	p.H1047R	3	178,952,085	178,952,085	A	A	G	Missense_Mutation	TRUE	FALSE
MSK-META-10	Metaplastic Carcinoma	Metastasis	IRS1	p.F1188L	2	227,659,891	227,659,891	G	G	T	Missense_Mutation	FALSE	FALSE
MSK-META-10	Metaplastic Carcinoma	Metastasis	TP53	p.P36Afs*7	17	7,579,585	7,579,586	-	-	G	Frame_Shift_Ins	FALSE	FALSE
MSK-META-10	Metaplastic Carcinoma	Metastasis	SOX9	p.Q500Hfs*74	17	70,120,495	70,120,505	ACAACCCGTCT	ACAACCCGTCT	-	Frame_Shift_Del	FALSE	FALSE
MSK-META-2	Metaplastic Carcinoma	Metastasis	TP53	p.C275R	17	7,577,115	7,577,115	A	A	G	Missense_Mutation	TRUE	TRUE
MSK-META-2	Metaplastic Carcinoma	Metastasis	FBXW7	p.E588*	4	153,245,429	153,245,429	C	C	A	Nonsense_Mutation	FALSE	TRUE
MSK-META-2	Metaplastic Carcinoma	Metastasis	RICTOR	p.R401H	5	38,967,279	38,967,279	C	C	T	Missense_Mutation	FALSE	FALSE
MSK-META-2	Metaplastic Carcinoma	Metastasis	BRIP1	p.I504F	17	59,861,749	59,861,749	T	T	A	Missense_Mutation	FALSE	TRUE
MSK-META-2	Metaplastic Carcinoma	Metastasis	BCOR	p.D1336H	X	39,922,166	39,922,166	C	C	G	Missense_Mutation	FALSE	TRUE
MSK-META-2	Metaplastic Carcinoma	Metastasis	PIK3CA	p.L452_G460del	3	178,928,072	178,928,098	TGGATTAGAAGATTTGCTGAACCCTAT	TGGATTAGAAGATTTGCTGAACCCTAT	-	In_Frame_Del	TRUE	FALSE
MSK-META-7	Metaplastic Carcinoma	Metastasis	TP53	p.R248Q	17	7,577,538	7,577,538	C	C	T	Missense_Mutation	TRUE	TRUE
MSK-META-7	Metaplastic Carcinoma	Metastasis	IFNGR1	p.R409S	6	137,519,411	137,519,411	T	T	G	Missense_Mutation	FALSE	TRUE
MSK-META-1	Metaplastic Carcinoma	Primary	PIK3CA	p.C420R	3	178,927,980	178,927,980	T	T	C	Missense_Mutation	TRUE	FALSE
MSK-META-1	Metaplastic Carcinoma	Primary	TP53	p.V274A	17	7,577,117	7,577,117	A	A	G	Missense_Mutation	TRUE	TRUE
MSK-META-1	Metaplastic Carcinoma	Primary	PTEN	p.X70_splice	10	89,685,316	89,685,316	T	T	C	Splice_Site	TRUE	FALSE
MSK-META-4	Metaplastic Carcinoma	Metastasis	TP53	p.K132R	17	7,578,535	7,578,535	T	T	C	Missense_Mutation	TRUE	FALSE
MSK-META-3	Metaplastic Carcinoma	Metastasis	TERT		5	1,295,228	1,295,228	G	G	A	Promoter	FALSE	FALSE
MSK-META-3	Metaplastic Carcinoma	Metastasis	PIK3CA	p.H1047R	3	178,952,085	178,952,085	A	A	G	Missense_Mutation	TRUE	FALSE
MSK-MPC-16	Micropapillary Carcinoma	Metastasis	TERT		5	1,295,228	1,295,228	G	G	A	Promoter	FALSE	FALSE
MSK-MPC-16	Micropapillary Carcinoma	Metastasis	TP53	p.E286V	17	7,577,081	7,577,081	T	T	A	Missense_Mutation	TRUE	TRUE
MSK-MPC-16	Micropapillary Carcinoma	Metastasis	ARID1B	p.L1634I	6	157,525,044	157,525,044	C	C	A	Missense_Mutation	FALSE	FALSE
MSK-MPC-16	Micropapillary Carcinoma	Metastasis	DDR2	p.E262D	1	162,729,700	162,729,700	G	G	C	Missense_Mutation	FALSE	FALSE
MSK-MPC-16	Micropapillary Carcinoma	Metastasis	KDR	p.Q67H	4	55,984,928	55,984,928	C	C	G	Missense_Mutation	FALSE	FALSE
MSK-MPC-16	Micropapillary Carcinoma	Metastasis	KDM6A	p.A12G	X	44,732,832	44,732,832	C	C	G	Missense_Mutation	FALSE	FALSE
MSK-MPC-16	Micropapillary Carcinoma	Metastasis	FOXA1	p.K264_K267del	14	38,061,188	38,061,199	CTTGAAGCGCTT	CTTGAAGCGCTT	-	In_Frame_Del	TRUE	TRUE
MSK-MPC-16	Micropapillary Carcinoma	Metastasis	PIK3CA	p.L455Wfs*6	3	178,928,083	178,928,105	ATTTGCTGAACCCTATTGGTGTT	ATTTGCTGAACCCTATTGGTGTT	-	Frame_Shift_Del	FALSE	FALSE
MSK-MPC-16	Micropapillary Carcinoma	Metastasis	PIK3CA	p.L452Qfs*5	3	178,928,076	178,928,077	TT	TT	C	Frame_Shift_Del	FALSE	FALSE
MSK-MPC-4	Micropapillary Carcinoma	Metastasis	TP53	p.C176*	17	7,578,401	7,578,402	GG	GG	T	Nonsense_Mutation	TRUE	FALSE
MSK-MPC-20	Micropapillary Carcinoma	Metastasis	TP53	p.S241F	17	7,577,559	7,577,559	G	G	A	Missense_Mutation	TRUE	FALSE
MSK-MPC-20	Micropapillary Carcinoma	Metastasis	KIT	p.L793S	4	55,599,252	55,599,252	T	T	C	Missense_Mutation	FALSE	FALSE
MSK-MPC-20	Micropapillary Carcinoma	Metastasis	ATR	p.A14S	3	142,297,507	142,297,507	C	C	A	Missense_Mutation	FALSE	TRUE
MSK-MPC-20	Micropapillary Carcinoma	Metastasis	KDR	p.E674D	4	55,968,641	55,968,641	C	C	G	Missense_Mutation	FALSE	FALSE
MSK-MPC-20	Micropapillary Carcinoma	Metastasis	NKX2-1	p.S336N	14	36,986,592	36,986,592	C	C	T	Missense_Mutation	FALSE	FALSE
MSK-MPC-20	Micropapillary Carcinoma	Metastasis	NCOR1	p.K683*	17	16,021,210	16,021,210	T	T	A	Nonsense_Mutation	FALSE	FALSE
MSK-MPC-20	Micropapillary Carcinoma	Metastasis	EPHB1	p.F404Lfs*44	3	134,851,802	134,851,802	C	C	-	Frame_Shift_Del	FALSE	FALSE
MSK-MPC-20	Micropapillary Carcinoma	Metastasis	PIK3CB	p.L591_Q592del	3	138,413,744	138,413,749	CTGAAG	CTGAAG	-	In_Frame_Del	FALSE	FALSE
MSK-MPC-20	Micropapillary Carcinoma	Metastasis	KMT2C	p.I244L	7	151,848,029	151,848,029	T	T	G	Missense_Mutation	FALSE	FALSE
MSK-MPC-7	Micropapillary Carcinoma	Primary	TP53	p.R273C	17	7,577,121	7,577,121	G	G	A	Missense_Mutation	TRUE	TRUE
MSK-MPC-7	Micropapillary Carcinoma	Primary	ARID2	p.A85V	12	46,125,067	46,125,067	C	C	T	Missense_Mutation	FALSE	FALSE
MSK-MPC-7	Micropapillary Carcinoma	Primary	PTPRS	p.D1522N	19	5,214,422	5,214,422	C	C	T	Missense_Mutation	FALSE	FALSE
MSK-MPC-7	Micropapillary Carcinoma	Primary	ALK	p.I839T	2	29,455,286	29,455,286	A	A	G	Missense_Mutation	FALSE	FALSE
MSK-MPC-7	Micropapillary Carcinoma	Primary	SETD2	p.S996*	3	47,163,139	47,163,139	G	G	C	Nonsense_Mutation	FALSE	FALSE
MSK-MPC-1	Micropapillary Carcinoma	Primary	GATA3	p.D335Gfs*17	10	8,111,514	8,111,515	-	-	G	Frame_Shift_Ins	FALSE	TRUE
MSK-MPC-1	Micropapillary Carcinoma	Primary	SOX17	p.G310S	8	55,372,238	55,372,238	G	G	A	Missense_Mutation	FALSE	FALSE
MSK-MPC-5	Micropapillary Carcinoma	Metastasis	PIK3CA	p.H1047R	3	178,952,085	178,952,085	A	A	G	Missense_Mutation	TRUE	FALSE
MSK-MPC-5	Micropapillary Carcinoma	Metastasis	NOTCH1	p.Y891H	9	139,405,174	139,405,174	A	A	G	Missense_Mutation	FALSE	TRUE
MSK-MPC-15	Micropapillary Carcinoma	Metastasis	ERBB2	p.L755S	17	37,880,220	37,880,220	T	T	C	Missense_Mutation	TRUE	FALSE
MSK-MPC-15	Micropapillary Carcinoma	Metastasis	RNF43	p.I141V	17	56,440,916	56,440,916	T	T	C	Missense_Mutation	FALSE	FALSE
MSK-MPC-8	Micropapillary Carcinoma	Metastasis	PIK3CA	p.N345K	3	178,921,553	178,921,553	T	T	A	Missense_Mutation	TRUE	FALSE
MSK-MPC-8	Micropapillary Carcinoma	Metastasis	PAK7	p.M509I	20	9,543,627	9,543,627	C	C	T	Missense_Mutation	FALSE	FALSE
MSK-MPC-8	Micropapillary Carcinoma	Metastasis	AMER1	p.R699C	X	63,411,072	63,411,072	G	G	A	Missense_Mutation	FALSE	TRUE
MSK-MPC-6	Micropapillary Carcinoma	Primary	MAP3K1	p.T657Hfs*2	5	56,174,807	56,174,807	A	A	-	Frame_Shift_Del	FALSE	FALSE
MSK-MPC-12	Micropapillary Carcinoma	Metastasis	PIK3CA	p.C420R	3	178,927,980	178,927,980	T	T	C	Missense_Mutation	TRUE	FALSE
MSK-MPC-12	Micropapillary Carcinoma	Metastasis	TP53	p.F113V	17	7,579,350	7,579,350	A	A	C	Missense_Mutation	TRUE	TRUE
MSK-MPC-12	Micropapillary Carcinoma	Metastasis	ARID1A	p.H495D	1	27,057,775	27,057,775	C	C	G	Missense_Mutation	FALSE	FALSE
MSK-MPC-12	Micropapillary Carcinoma	Metastasis	TSC1	p.L463Ffs*69	9	135,782,166	135,782,167	CT	CT	G	Frame_Shift_Del	FALSE	FALSE
MSK-MPC-12	Micropapillary Carcinoma	Metastasis	ARID1A	p.A325Rfs*51	1	27,023,863	27,023,933	GGGCGCCGGCAAGGGCCCGGCGGACATGGCCTCGCAGTGTTGGGGGGCTGCGGCGGCGGCAGCTGCGGCGG	GGGCGCCGGCAAGGGCCCGGCGGACATGGCCTCGCAGTGTTGGGGGGCTGCGGCGGCGGCAGCTGCGGCGG	-	Frame_Shift_Del	FALSE	FALSE
MSK-MPC-11	Micropapillary Carcinoma	Primary	PIK3CA	p.H1047R	3	178,952,085	178,952,085	A	A	G	Missense_Mutation	TRUE	FALSE
MSK-MPC-13	Micropapillary Carcinoma	Metastasis	PIK3CA	p.C420R	3	178,927,980	178,927,980	T	T	C	Missense_Mutation	TRUE	FALSE
MSK-MPC-13	Micropapillary Carcinoma	Metastasis	TP53	p.F113V	17	7,579,350	7,579,350	A	A	C	Missense_Mutation	TRUE	TRUE
MSK-MPC-10	Micropapillary Carcinoma	Primary	PIK3CA	p.H1047R	3	178,952,085	178,952,085	A	A	G	Missense_Mutation	TRUE	FALSE
MSK-MPC-10	Micropapillary Carcinoma	Primary	PIK3CA	p.E542A	3	178,936,083	178,936,083	A	A	C	Missense_Mutation	TRUE	FALSE
MSK-MPC-10	Micropapillary Carcinoma	Primary	FLT4	p.X329_splice	5	180,056,000	180,056,000	C	C	A	Splice_Site	FALSE	FALSE
MSK-MPC-10	Micropapillary Carcinoma	Primary	NCOR1	p.S88R	17	16,075,288	16,075,288	A	A	C	Missense_Mutation	FALSE	FALSE
MSK-MPC-17	Micropapillary Carcinoma	Primary	TP53	p.Q331*	17	7,576,855	7,576,855	G	G	A	Nonsense_Mutation	TRUE	FALSE
MSK-MPC-17	Micropapillary Carcinoma	Primary	PIK3R2	p.E193Q	19	18,271,974	18,271,974	G	G	C	Missense_Mutation	FALSE	TRUE
MSK-MPC-14	Micropapillary Carcinoma	Metastasis	PIK3CA	p.E545K	3	178,936,091	178,936,091	G	G	A	Missense_Mutation	TRUE	FALSE
MSK-MPC-14	Micropapillary Carcinoma	Metastasis	ESR1	p.L536H	6	152,419,920	152,419,920	T	T	A	Missense_Mutation	TRUE	FALSE
MSK-MPC-18	Micropapillary Carcinoma	Metastasis	PIK3CA	p.H1047R	3	178,952,085	178,952,085	A	A	G	Missense_Mutation	TRUE	FALSE
MSK-MPC-18	Micropapillary Carcinoma	Metastasis	TET1	p.E350K	10	70,333,143	70,333,143	G	G	A	Missense_Mutation	FALSE	FALSE
MSK-MPC-18	Micropapillary Carcinoma	Metastasis	SMARCA4	p.T858M	19	11,130,334	11,130,334	C	C	T	Missense_Mutation	FALSE	TRUE
MSK-MPC-3	Micropapillary Carcinoma	Metastasis	PMS1	p.C101*	2	190,660,665	190,660,665	T	T	A	Nonsense_Mutation	FALSE	FALSE
MSK-MPC-3	Micropapillary Carcinoma	Metastasis	BARD1	p.G345E	2	215,645,564	215,645,564	C	C	T	Missense_Mutation	FALSE	FALSE
MSK-MPC-3	Micropapillary Carcinoma	Metastasis	ATR	p.K338T	3	142,281,231	142,281,231	T	T	G	Missense_Mutation	FALSE	TRUE
MSK-MPC-3	Micropapillary Carcinoma	Metastasis	FAT1	p.Q1421K	4	187,554,900	187,554,900	G	G	T	Missense_Mutation	FALSE	TRUE
MSK-MPC-3	Micropapillary Carcinoma	Metastasis	ESR1	p.D538G	6	152,419,926	152,419,926	A	A	G	Missense_Mutation	TRUE	FALSE
MSK-MPC-3	Micropapillary Carcinoma	Metastasis	ARID1B	p.E1854G	6	157,527,875	157,527,875	A	A	G	Missense_Mutation	FALSE	FALSE
MSK-MPC-3	Micropapillary Carcinoma	Metastasis	PTPRD	p.X608_splice	9	8,501,060	8,501,060	C	C	G	Splice_Site	FALSE	TRUE
MSK-MPC-3	Micropapillary Carcinoma	Metastasis	ARID2	p.S1137P	12	46,245,315	46,245,315	T	T	C	Missense_Mutation	FALSE	FALSE
MSK-MPC-3	Micropapillary Carcinoma	Metastasis	HNF1A	p.R321C	12	121,434,070	121,434,070	C	C	T	Missense_Mutation	FALSE	FALSE
MSK-MPC-3	Micropapillary Carcinoma	Metastasis	BRCA2	p.Y3049C	13	32,954,172	32,954,172	A	A	G	Missense_Mutation	FALSE	FALSE
MSK-MPC-3	Micropapillary Carcinoma	Metastasis	CDK12	p.K756T	17	37,650,795	37,650,795	A	A	C	Missense_Mutation	FALSE	TRUE
MSK-MPC-3	Micropapillary Carcinoma	Metastasis	BRIP1	p.Q524L	17	59,861,688	59,861,688	T	T	A	Missense_Mutation	FALSE	TRUE
MSK-MPC-3	Micropapillary Carcinoma	Metastasis	PTPRS	p.S552I	19	5,240,259	5,240,259	C	C	A	Missense_Mutation	FALSE	FALSE
MSK-MPC-3	Micropapillary Carcinoma	Metastasis	SPOP	p.M288_S298del	17	47,679,313	47,679,345	GGACAGGTTACTGCAGAGGGCATCCTCACACAT	GGACAGGTTACTGCAGAGGGCATCCTCACACAT	-	In_Frame_Del	FALSE	TRUE
MSK-MPC-3	Micropapillary Carcinoma	Metastasis	TSC2	p.R1205fs*5	16	2,104,320	2,104,321	AG	AGCCCTCTTCTTTAAGGTCATCA	-	Frame_Shift_Del	FALSE	FALSE
MSK-MPC-3	Micropapillary Carcinoma	Metastasis	HIST1H2BD	p.G115Dfs*28	6	26,158,741	26,158,742	GC	GCCGTCACCAAG	-	Frame_Shift_Del	FALSE	FALSE
MSK-MUC-11	Mucinous Carcinoma	Primary	GATA3	p.M356_K387del	10	8,115,720	8,115,815	ATGAAGAAGGAAGGCATCCAGACCAGAAACCGAAAAATGTCTAGCAAATCCAAAAAGTGCAAAAAAGTGCATGACTCACTGGAGGACTTCCCCAAG	ATGAAGAAGGAAGGCATCCAGACCAGAAACCGAAAAATGTCTAGCAAATCCAAAAAGTGCAAAAAAGTGCATGACTCACTGGAGGACTTCCCCAAG	-	In_Frame_Del	FALSE	TRUE
MSK-MUC-2	Mucinous Carcinoma	Metastasis	PIK3CA	p.H1047R	3	178,952,085	178,952,085	A	A	G	Missense_Mutation	TRUE	FALSE
MSK-MUC-2	Mucinous Carcinoma	Metastasis	TP53	p.R213*	17	7,578,212	7,578,212	G	G	A	Nonsense_Mutation	TRUE	TRUE
MSK-MUC-2	Mucinous Carcinoma	Metastasis	CBFB	p.X55_splice	16	67,063,717	67,063,718	-	-	T	Splice_Site	FALSE	FALSE
MSK-MUC-2	Mucinous Carcinoma	Metastasis	ARID1A	p.G1062D	1	27,094,477	27,094,477	G	G	A	Missense_Mutation	FALSE	TRUE
MSK-MUC-2	Mucinous Carcinoma	Metastasis	PIK3CA	p.C407F	3	178,927,457	178,927,457	G	G	T	Missense_Mutation	FALSE	FALSE
MSK-MUC-2	Mucinous Carcinoma	Metastasis	NCOR1	p.S2335Y	17	15,938,210	15,938,210	G	G	T	Missense_Mutation	FALSE	TRUE
MSK-MUC-2	Mucinous Carcinoma	Metastasis	GATA3	p.X350_splice	10	8,115,623	8,115,708	TTCTTGGTGTGCGAGAGCCTGTGCATTTCAGAGGCAGCAAAAAAGTAAAAAAAAAAAAAAAAAATTGATCTTTGTTTAGATTAACA	TTCTTGGTGTGCGAGAGCCTGTGCATTTCAGAGGCAGCAAAAAAGTAAAAAAAAAAAAAAAAAATTGATCTTTGTTTAGATTAACA	-	Splice_Site	FALSE	FALSE
MSK-MUC-19	Mucinous Carcinoma	Primary	GATA3	p.P424Rfs*51	10	8,115,923	8,115,923	C	C	-	Frame_Shift_Del	FALSE	FALSE
MSK-MUC-18	Mucinous Carcinoma	Primary	ALK	p.W288*	2	29,917,805	29,917,805	C	C	T	Nonsense_Mutation	FALSE	FALSE
MSK-MUC-10	Mucinous Carcinoma	Primary	CDK12	p.W719*	17	37,649,052	37,649,052	G	G	A	Nonsense_Mutation	FALSE	TRUE
MSK-MUC-1	Mucinous Carcinoma	Primary	PTEN	p.Y346*	10	89,725,055	89,725,055	C	C	G	Nonsense_Mutation	TRUE	FALSE
MSK-MUC-1	Mucinous Carcinoma	Primary	MAP3K1	p.X1204_splice	5	56,178,638	56,178,707	TAGTTCCTCAGCTGCAGGTTGAAAATGGAGAAGATATCATCATTATTCAACAGGATGTAAGTATAGATTC	TAGTTCCTCAGCTGCAGGTTGAAAATGGAGAAGATATCATCATTATTCAACAGGATGTAAGTATAGATTC	-	Splice_Site	FALSE	FALSE
MSK-MUC-9	Mucinous Carcinoma	Primary	NSD1	p.Q2669R	5	176,722,375	176,722,375	A	A	G	Missense_Mutation	FALSE	FALSE
MSK-MUC-9	Mucinous Carcinoma	Primary	GATA3	p.S401Vfs*106	10	8,115,854	8,115,855	-	-	G	Frame_Shift_Ins	FALSE	TRUE
MSK-MUC-5	Mucinous Carcinoma	Metastasis	PAK7	p.Q220H	20	9,561,122	9,561,122	C	C	A	Missense_Mutation	FALSE	FALSE
MSK-MUC-5	Mucinous Carcinoma	Metastasis	GATA3	p.P408Afs*99	10	8,115,875	8,115,876	-	-	G	Frame_Shift_Ins	FALSE	TRUE
MSK-MUC-7	Mucinous Carcinoma	Metastasis	ESR1	p.Y537C	6	152,419,923	152,419,923	A	A	G	Missense_Mutation	TRUE	FALSE
MSK-MUC-7	Mucinous Carcinoma	Metastasis	EGFR	p.C636W	7	55,238,895	55,238,895	T	T	G	Missense_Mutation	FALSE	FALSE
MSK-MUC-7	Mucinous Carcinoma	Metastasis	PIK3C2G	p.I271F	12	18,443,838	18,443,838	A	A	T	Missense_Mutation	FALSE	FALSE
MSK-MUC-7	Mucinous Carcinoma	Metastasis	FLT1	p.I692S	13	28,959,063	28,959,063	A	A	C	Missense_Mutation	FALSE	FALSE
MSK-MUC-7	Mucinous Carcinoma	Metastasis	MAP2K4	p.R134W	17	11,998,898	11,998,898	C	C	T	Missense_Mutation	TRUE	TRUE
MSK-MUC-7	Mucinous Carcinoma	Metastasis	NCOR1	p.L1627V	17	15,968,871	15,968,871	A	A	C	Missense_Mutation	FALSE	TRUE
MSK-MUC-7	Mucinous Carcinoma	Metastasis	ATM	p.S2812Vfs*3	11	108,216,477	108,216,478	-	-	A	Frame_Shift_Ins	FALSE	TRUE
MSK-MUC-8	Mucinous Carcinoma	Primary	GATA3	p.P408Afs*99	10	8,115,874	8,115,875	-	-	C	Frame_Shift_Ins	FALSE	FALSE
MSK-MUC-15	Mucinous Carcinoma	Metastasis	ERBB2	p.E770_A771insGIRD	17	37,880,982	37,880,983	-	-	GCATACGTGATG	In_Frame_Ins	FALSE	FALSE
MSK-MUC-15	Mucinous Carcinoma	Metastasis	ABL1	p.S19L	9	133,710,889	133,710,889	C	C	T	Missense_Mutation	FALSE	FALSE
MSK-MUC-15	Mucinous Carcinoma	Metastasis	FANCA	p.L1038V	16	89,816,265	89,816,265	G	G	C	Missense_Mutation	FALSE	FALSE
MSK-MUC-15	Mucinous Carcinoma	Metastasis	MAP3K1	p.Y1319Lfs*6	5	56,180,625	56,180,626	-	-	T	Frame_Shift_Ins	FALSE	FALSE
MSK-MUC-15	Mucinous Carcinoma	Metastasis	MAP3K1	p.S537Qfs*3	5	56,168,751	56,168,752	AG	AG	-	Frame_Shift_Del	FALSE	FALSE
MSK-MUC-13	Mucinous Carcinoma	Primary	STK11	p.T189Qfs*75	19	1,220,471	1,220,475	CACCC	CACCC	-	Frame_Shift_Del	FALSE	TRUE
MSK-MUC-6	Mucinous Carcinoma	Primary	BRCA2	p.S278N	13	32,906,448	32,906,448	G	G	A	Missense_Mutation	FALSE	FALSE
MSK-MUC-6	Mucinous Carcinoma	Primary	RPTOR	p.T917I	17	78,897,415	78,897,415	C	C	T	Missense_Mutation	FALSE	FALSE
MSK-MUC-14	Mucinous Carcinoma	Primary	PIK3CA	p.E545K	3	178,936,091	178,936,091	G	G	A	Missense_Mutation	TRUE	FALSE
MSK-MUC-14	Mucinous Carcinoma	Primary	GATA3	p.A441Rfs*66	10	8,115,973	8,115,974	-	-	C	Frame_Shift_Ins	FALSE	FALSE
MSK-MUC-14	Mucinous Carcinoma	Primary	TET2	p.G1288V	4	106,180,835	106,180,835	G	G	T	Missense_Mutation	FALSE	FALSE
MSK-MUC-4	Mucinous Carcinoma	Metastasis	ESR1	p.D538G	6	152,419,926	152,419,926	A	A	G	Missense_Mutation	TRUE	FALSE
MSK-MUC-4	Mucinous Carcinoma	Metastasis	GATA3	p.S407Afs*99	10	8,115,871	8,115,872	TC	TC	-	Frame_Shift_Del	FALSE	FALSE
MSK-MUC-4	Mucinous Carcinoma	Metastasis	RAD51B	p.Q371*	14	69,061,276	69,061,276	C	C	T	Nonsense_Mutation	FALSE	FALSE
MSK-MUC-17	Mucinous Carcinoma	Primary	AKT1	p.E17K	14	105,246,551	105,246,551	C	C	T	Missense_Mutation	TRUE	FALSE

Supplementary Table 5. Therapy received prior to sampling of metastatic lobular carcinomas according to *ERBB2* mutational status

**Pre-biopsy therapy**	***ERBB2* L755S (*n*=7)**	**Other *ERBB2* mutations (*n* =9)**	**No *ERBB2* mutations (n=98)**	***P* value***
Chemotherapy-containing	0 (0%)	0 (0%)	4 (4.1%)	n.s.
Chemotherapy-containing/Anti-HER2	0 (0%)	0 (0%)	1 (1%)	
Chemotherapy-containing/Hormone-therapy	6 (85.7%)	7 (77.8%)	72 (73.5%)	
Chemotherapy-containing/Hormone-therapy/Anti-HER2	1 (14.3%)	0 (0%)	4 (4.1%)	
Anti-HER2	0 (0%)	0 (0%)	3 (3.1%)	
Hormone-therapy	0 (0%)	1 (11.1%)	13 (13.3%)	
Hormone-therapy/Anti-HER2	0 (0%)	1 (11.1%)	1 (1%)	
**Last line of hormone therapy prior to biopsy**	***ERBB2* L755S (*n*=7)**	**Other *ERBB2* mutations (*n* =9)**	**No *ERBB2* mutations (*n* =90)**	***P* value***
Aromatase inhibitors	5 (71.4%)	6 (66.7%)	51 (56.7%)	n.s.
Selective estrogen receptor degraders (SERD)	1 (14.3%)	0 (0%)	11 (12.2%)	
Selective estrogen receptor modulators (SERM)	1 (14.3%)	3 (33.3%)	28 (31.1%)	

*Chi square test

n.s., not significant

Supplementary Table 6. Therapy received prior to sampling of metastatic lobular carcinomas according to *ERBB2* mutational status

Pre-biopsy therapy	mILC (n=114)	mIDC (n=235)	*P* value*	mMUC (n=5)	mIDC (n=13)	*P* value*	mMPC (n=9)	mIDC (n=33)	*P* value*	mMETA (n=6)	mIDC (n=18)	*P* value*
Chemotherapy-containing	4 (3.5%)	27 (11.5%)	n.s.	0 (0%)	1 (7.7%)	n.s.	1 (11.1%)	4 (12.1%)	n.s.	3 (50%)	8 (44.4%)	n.s.
Chemotherapy-containing/Anti-HER2	1 (0.9%)	0 (0%)		0 (0%)	0 (0%)		0 (0%)	0 (0%)		0 (0%)	0 (0%)	
Chemotherapy-containing/Hormone-therapy	85 (74.6%)	158 (67.2%)		3 (60%)	9 (69.2%)		6 (66.7%)	26 (78.8%)		2 (33.3%)	6 (33.3%)	
Chemotherapy-containing/Hormone-therapy/Anti-HER2	5 (4.4%)	4 (1.7%)		2 (40%)	0 (0%)		0 (0%)	0 (0%)		0 (0%)	0 (0%)	
Anti-HER2	3 (2.6%)	5 (2.1%)		0 (0%)	0 (0%)		0 (0%)	0 (0%)		1 (16.7%)	0 (0%)	
Hormone-therapy	14 (12.3%)	30 (12.8%)		0 (0%)	3 (23.1%		2 (22.2%)	3 (9.1%)		0 (0%)	3 (16.7%)	
Hormone-therapy/Anti-HER2	2 (1.8%)	11 (4.7%)		0 (0%)	0 (0%)		0 (0%)	0 (0%)		0 (0%)	1 (5.6%)	

*Chi square test

n.s., not significant
