# Supplementary material for: The genomic landscape of metastatic histologic special types of invasive breast cancer
Source: NPJ Breast Cancer. 2020 Oct 14;6:53. doi: 10.1038/s41523-020-00195-4 (PMC7560857; doi:10.1038/s41523-020-00195-4)
Supplement: Supplementary file 2 — Reporting Summary Checklist FLAT [file 41523_2020_195_MOESM2_ESM.pdf]

nature research

Corresponding author(s): Fresia Pareja, MD PhD
Britta Weigelt, PhD
Jorge S. Reis-Filho, MD PhD FRCPath

Last updated by author(s): Sep 4, 2020

# Reporting Summary

Nature Research wishes to improve the reproducibility of the work that we publish. This form provides structure for consistency and transparency in reporting. For further information on Nature Research policies, see our Editorial Policies and the Editorial Policy Checklist.

## Statistics

For all statistical analyses, confirm that the following items are present in the figure legend, table legend, main text, or Methods section.

| n/a | Confirmed | |
|---|---|---|
| ☐ | ☒ | The exact sample size (*n*) for each experimental group/condition, given as a discrete number and unit of measurement |
| ☒ | ☐ | A statement on whether measurements were taken from distinct samples or whether the same sample was measured repeatedly |
| ☐ | ☒ | The statistical test(s) used AND whether they are one- or two-sided<br>*Only common tests should be described solely by name; describe more complex techniques in the Methods section.* |
| ☒ | ☐ | A description of all covariates tested |
| ☐ | ☒ | A description of any assumptions or corrections, such as tests of normality and adjustment for multiple comparisons |
| ☐ | ☒ | A full description of the statistical parameters including central tendency (e.g. means) or other basic estimates (e.g. regression coefficient) AND variation (e.g. standard deviation) or associated estimates of uncertainty (e.g. confidence intervals) |
| ☐ | ☒ | For null hypothesis testing, the test statistic (e.g. *F*, *t*, *r*) with confidence intervals, effect sizes, degrees of freedom and *P* value noted<br>*Give P values as exact values whenever suitable.* |
| ☒ | ☐ | For Bayesian analysis, information on the choice of priors and Markov chain Monte Carlo settings |
| ☒ | ☐ | For hierarchical and complex designs, identification of the appropriate level for tests and full reporting of outcomes |
| ☒ | ☐ | Estimates of effect sizes (e.g. Cohen's *d*, Pearson's *r*), indicating how they were calculated |

*Our web collection on statistics for biologists contains articles on many of the points above.*

## Software and code

Policy information about availability of computer code

| | |
|---|---|
| Data collection | All samples included in this study were subjected to targeted sequencing using the FDA-approved MSK Integrated Mutation Profiling of Actionable Targets (MSK-IMPACT) assay (PMID: 25801821) as part of the study by Razavi et al. (PMID: 30205045). |
| Data analysis | Non-synonymous somatic mutations, amplifications and homozygous deletions were retrieved from the original study. The raw MSK-IMPACT sequencing data (i.e., FASTQ files) were re-processed using our validated bioinformatics pipeline, for the inference of copy number gains and losses, and loss of heterozygosity (LOH) of genes targeted by somatic mutations and mutational signatures. Non-synonymous tumor mutation burden was calculated as the number of non-synonymous mutations divided by the total genomic region assessed by MSK-IMPACT, per megabase. The fraction of genome altered defined as the number of base pairs which are not copy neutral divided by the size of genome assayed, was retrieved from the original study by Razavi et al (PMID: 30205045). Mutational signatures were defined using SigMA (PMID: 30988514) using all synonymous and non-synonymous somatic mutations of cases with at least five single nucleotide variants. Tumor purity was inferred using FACETS (PMID: 27270079). |

For manuscripts utilizing custom algorithms or software that are central to the research but not yet described in published literature, software must be made available to editors and reviewers. We strongly encourage code deposition in a community repository (e.g. GitHub). See the Nature Research guidelines for submitting code & software for further information.

## Data

Policy information about availability of data

All manuscripts must include a data availability statement. This statement should provide the following information, where applicable:

- Accession codes, unique identifiers, or web links for publicly available datasets
- A list of figures that have associated raw data
- A description of any restrictions on data availability

The data generated and analysed during this study are described in the following data record:https://doi.org/10.6084/m9.figshare.12855149. Histologic images supporting Figures 1, 2, 3, 4, and Supplementary Figure 7 are not publicly available, but can be requested from the corresponding author, Dr. Fresia Pareja. MSK-IMPACT sequencing data supporting Figures 1, 2, 3, 4, 5, Supplementary Figures 1, 2, 3, 4, 5, 6, and Supplementary Table 3, 4, 5 are publicly available in cBioPortal at the following accession: https://identifiers.org/cbioportal:breast_msk_2018. Clinical data supporting supplementary Tables 1, 2, 5 and 6 are available in the original publication by Razavi et al.

# Field-specific reporting

Please select the one below that is the best fit for your research. If you are not sure, read the appropriate sections before making your selection.

☒ Life sciences ☐ Behavioural & social sciences ☐ Ecological, evolutionary & environmental sciences

For a reference copy of the document with all sections, see nature.com/documents/nr-reporting-summary-flat.pdf

# Life sciences study design

All studies must disclose on these points even when the disclosure is negative.

| | |
|---|---|
| Sample size | 309 breast cancers of special histologic type were included in this study, with following the criteria put forward by the World Health Organization, were classified as classic invasive lobular carcinomas (n=127 metastatic and n=132 primary), 19 as mixed mucinous carcinomas (n=5 metastatic and n=14 primary), 20 as pure micropapillary carcinomas (n=12 metastatic and n=8 primary) and 11 as metaplastic breast cancers (n=6 metastatic and n=5 primary). |
| Data exclusions | Pleomorphic ILCs were excluded from further analyses. |
| Replication | N/A |
| Randomization | No randomization was conducted in this study. |
| Blinding | Blinding was not relevant to this study. |

# Reporting for specific materials, systems and methods

We require information from authors about some types of materials, experimental systems and methods used in many studies. Here, indicate whether each material, system or method listed is relevant to your study. If you are not sure if a list item applies to your research, read the appropriate section before selecting a response.

### Materials & experimental systems

| n/a | Involved in the study | |
|---|---|---|
| ☐ | ☒ | Antibodies |
| ☒ | ☐ | Eukaryotic cell lines |
| ☒ | ☐ | Palaeontology and archaeology |
| ☒ | ☐ | Animals and other organisms |
| ☒ | ☐ | Human research participants |
| ☒ | ☐ | Clinical data |
| ☒ | ☐ | Dual use research of concern |

### Methods

| n/a | Involved in the study | |
|---|---|---|
| ☒ | ☐ | ChIP-seq |
| ☒ | ☐ | Flow cytometry |
| ☒ | ☐ | MRI-based neuroimaging |

## Antibodies

| | |
|---|---|
| Antibodies used | Immunohistochemical analyses for MLH1, MSH2, MSH6 and PMS2 with the following antibodies: MLH1 (clone ES05; Leica Biosystems), MSH2 (clone G219-1129; Cell Marque), MSH6 (clone EP49; Dako) or PMS2 (clone A16.4; BD Biosciences). |
| Validation | Antibodies were validated for immunohistochemistry with positive and negative controls and have been previously cited for the interested use. |
